# Male Reproductive Disorders Associated with Antiseizure Medications: Evidence from Meta-Analysis and FAERS Pharmacovigilance Study

**DOI:** 10.3390/jcm15197566

**Published:** 2026-09-29

**Authors:** Heejin Na, Boyoon Choi, Se-Hoon Oh, In-je Jo, Dae Keun Kim, Yun-Kyoung Song

**Affiliations:** 1 Program of Biopharmaceutical Science, Department of Pharmacy, College of Pharmacy, Institute of Pharmaceutical Sciences, CHA University, Pocheon 11160, Republic of Korea; naheejin@chauniv.ac.kr (H.N.); boyoon1@cha.ac.kr (B.C.); 2 College of Pharmacy, The Catholic University of Korea, Bucheon-si 14662, Republic of Korea; osh9807@catholic.ac.kr; 3 College of Pharmacy, Daegu Catholic University, Gyeongsan 38430, Republic of Korea; cyd1234567@cu.ac.kr; 4 Department of Urology, CHA Fertility Center Seoul Station, CHA University School of Medicine, Seoul 06135, Republic of Korea; dkkim@cha.ac.kr

**Keywords:** antiseizure medications, FAERS, male reproductive disorders, meta-analysis, pharmacovigilance

## Abstract

**Background/Objectives**: Antiseizure medications (ASMs) are widely used in the management of epilepsy; however, their effects on male reproductive function in humans remain incompletely characterized. This study aimed to evaluate drug-specific associations between ASMs and male reproductive disorders through a meta-analysis of observational clinical studies and FDA Adverse Event Reporting System (FAERS)-based pharmacovigilance analyses. **Methods**: Clinical studies involving men with epilepsy receiving ASMs, untreated men with epilepsy, and healthy controls were systematically reviewed and quantitatively synthesized. FAERS reports from the first quarter of 2019 to the second quarter of 2026 (2019Q1–2026Q2) were analyzed using reporting odds ratios (RORs) and information components (ICs), with disproportionality signals identified by either method. **Results**: The meta-analysis included 20 clinical studies involving 1530 participants. Valproate was associated with adverse effects on reproductive hormones, sperm parameters, and sexual function, whereas carbamazepine and phenytoin were primarily associated with alterations in reproductive hormone levels. In the pharmacovigilance analysis, valproate showed a significant disproportionality signal for male reproductive disorders (IC_025_ 1.792). **Conclusions**: The findings indicate that male reproductive effects are not uniform across ASMs but exhibit distinct drug-specific patterns. Valproate was associated with alterations across multiple reproductive domains, whereas carbamazepine and phenytoin were associated predominantly with reproductive hormone alterations. Reproductive health should be considered when selecting and monitoring long-term ASM therapy in men, and prospective studies are needed to further characterize the reproductive safety profiles of individual ASMs.

## 1. Introduction

Male infertility, defined as the failure to achieve pregnancy after 12 months or more of regular unprotected sexual intercourse accompanied by abnormal semen parameters or sperm functional assays [1], has become a growing concern in recent decades. The global prevalence of male infertility has increased substantially since 1990, reaching approximately 56.53 million cases in 2019 [2]. This trend underscores the increasing global burden of male infertility and highlights the need to better understand factors that may adversely affect male reproductive health.

The etiology of male infertility is complex and multifactorial. Despite advances in diagnostic evaluation, approximately one-third of male infertility cases remain unexplained or idiopathic, leaving a substantial proportion of affected individuals without a definitive explanation for their condition [3]. Among potentially modifiable contributors, medications have been increasingly recognized as possible factors affecting male reproductive function. However, the evidence supporting medication-related male reproductive impairment in humans remains limited, and this issue remains underinvestigated [4]. Moreover, although male infertility is a clinically recognized outcome, it represents only one aspect of male reproductive health. In clinical and pharmacological contexts, male reproductive disorders encompass a broader spectrum of abnormalities, including hormonal imbalance, impaired spermatogenesis, testicular dysfunction, and sexual dysfunction. Therefore, a comprehensive assessment of male reproductive disorders is needed to better characterize the reproductive safety profiles of medications.

Men with epilepsy have been reported to have fertility rates one-third to two-thirds lower than those of the general population [5]. Reproductive hormonal disturbances and sexual dysfunction are also common among men with epilepsy, with reported prevalence estimates ranging from 28% to 71% [6,7,8]. Antiseizure medications (ASMs), formerly referred to as antiepileptic drugs, which are essential for epilepsy management, may also affect reproductive function [6,7,8,9,10]. However, the association between ASM exposure and male reproductive impairment remains uncertain. Some studies have suggested that ASMs may be associated with alterations in sex hormones [8,9,11,12,13,14,15,16,17,18,19,20,21,22,23,24,25,26], sperm quality [11,12,13,14,15,16,24,27,28], and sexual function [11,13,15,16], but methodological limitations and small sample sizes preclude definitive conclusions.

Conducting large randomized controlled trials in this context is challenging because of ethical concerns, the multifactorial nature of infertility, the relative rarity of adverse events (AEs), and the long follow-up required to assess reproductive outcomes [29]. As an alternative, real-world data (RWD), including observational clinical studies and pharmacovigilance databases such as the Food and Drug Administration (FDA) Adverse Event Reporting System (FAERS), can provide complementary evidence on the reproductive safety of ASMs [30].

Controlled observational studies provide clinically well-characterized evidence and enable quantitative assessment of reproductive outcomes but are often limited by small sample sizes and heterogeneous designs [31]. In contrast, FAERS offers large-scale, continuously updated AE reports across diverse populations and clinical settings, facilitating the detection of rare or underrecognized safety concerns, including emerging signals associated with newly marketed or increasingly prescribed drugs, that are not readily captured in clinical studies. However, FAERS-based analyses are subject to underreporting, confounding, and limited clinical detail; therefore, identified signals should be interpreted as potential safety concerns requiring further clinical evaluation [32].

Controlled observational studies and spontaneous-report pharmacovigilance address distinct aspects of drug safety. Meta-analysis can quantify associations between ASM exposure and clinically measured reproductive parameters under defined comparator conditions, whereas FAERS disproportionality analysis identifies reporting signals for reproductive adverse events. These approaches therefore provide complementary perspectives on ASM-related male reproductive safety.

Previous studies have largely focused on individual ASMs or limited reproductive outcomes. By evaluating multiple ASMs across reproductive hormones, semen parameters, and sexual function, using both healthy controls and untreated patients with epilepsy where data permitted, and separately assessing a broader range of reproductive adverse events in FAERS, the present study aimed to characterize drug-specific patterns of male reproductive effects associated with ASMs. Accordingly, we conducted a systematic review and meta-analysis of controlled clinical studies together with a parallel FAERS-based disproportionality analysis.

## 2. Materials and Methods

### 2.1. Meta-Analysis of Observational Clinical Studies

#### 2.1.1. Search Strategy

The protocol for the meta-analysis was registered in the PROSPERO database (CRD42024602937). This systematic review and meta-analysis were reported in accordance with the Meta-analysis of Observational Studies in Epidemiology (MOOSE) guidelines [33] and the Preferred Reporting Items for Systematic Reviews and Meta-Analyses (PRISMA) 2020 statement [34]. The completed PRISMA 2020 checklist is provided as Supplementary Table S1. Two independent authors (I. Jo. and B. Choi.) systematically searched MEDLINE via PubMed, EMBASE, and the Cochrane Central Register of Controlled Trials database.

The search strategy combined controlled vocabulary terms and relevant text keywords related to ASMs and male reproductive outcomes, including sperm parameters, gonadal hormones, sexual dysfunction, and male infertility. Animal-only studies were excluded while retaining studies involving human participants. The detailed search strategies for each database are provided in Supplementary Table S2. Only English-language studies were considered, and the final search was completed on 24 August 2026.

#### 2.1.2. Study Selection

Two authors (H. Na. and B. Choi.) independently screened the titles and abstracts of all retrieved articles. The full texts of potentially eligible articles were then obtained and assessed for eligibility. The inclusion criteria were: (i) studies investigating the association between ASM exposure and male reproductive outcomes; (ii) original research articles involving human subjects; and (iii) studies with available data sufficient to calculate the SMDs and 95% CIs. Exclusion criteria were: (i) studies on irrelevant topics; (ii) publications type other than an original research article; (iii) studies with an uncontrolled study design; (iv) non-human studies; (v) studies with ASM combination therapy; (vi) publications containing overlapping datasets; (vii) non-English publication; or (viii) non-English studies. Disagreements between reviewers were resolved through discussion with a third investigator (YK. Song.). Final consensus on study inclusion was reached after eligibility assessment and quality appraisal.

#### 2.1.3. Data Extraction and Quality Assessment

The data extraction forms were first piloted by I. Jo. and B. Choi. and subsequently applied to all eligible studies. Two authors (I. Jo. and H. Na.) independently extracted data from each study. Extracted data included publication year, first author’s surname, country, study design, sample size, participant characteristics (age, sex, and underlying diseases), control group characteristics (untreated patients or healthy controls), data type (paired/independent), treatment (ASMs), and clinical endpoints. Clinical endpoints were categorized into three groups: (i) sperm parameters, including progressive motility (%), head, neck/midpiece, and tail abnormalities (%), total sperm count (×10^6^/ejaculation), normal sperm morphology (%), semen volume (mL), and sperm concentration (×10^6^/mL); (ii) sex hormones, including dehydroepiandrosterone sulfate (DHEA-S, μmol/L), estradiol (E2, pmol/L), follicle stimulating hormone (FSH, mIU/mL), luteinizing hormone (LH, mIU/mL), prolactin (mIU/L), total testosterone (T, nmol/L), free T (pmol/L), sex hormone-binding globulin (SHBG, nmol/L), and free androgen index (FAI); and (iii) sexual dysfunction assessed using the International Index of Erectile Function-5 (IIEF-5) questionnaire scores (points).

For each endpoint, mean values and corresponding standard deviations were extracted. When standard deviations were not directly available, they were derived from reported 95% CIs when possible. For paired pre–post comparisons, the pre–post correlation coefficient was assumed to be 0.59 based on previous research [35]. Two investigators (I. Jo and H. Na.) independently evaluated the methodological quality of the included studies according to study design. Cross-sectional studies were assessed using the Appraisal Tool for Cross-Sectional Studies (AXIS) [36,37]. Before–after and prospective studies were assessed using an adapted Newcastle–Ottawa Scale (NOS) based on the cohort-study version, covering the domains of selection, comparability, and outcome assessment [38]. Any discrepancies in quality assessment were resolved through discussion with a third author (YK. Song.).

#### 2.1.4. Data Analysis

Endpoints reported in at least three studies were included in quantitative synthesis. Associations between ASM exposure and male reproductive outcomes were quantified using SMDs with 95% CIs for each endpoint. Analyses were stratified by control group type, namely untreated patients with epilepsy or healthy controls, and by individual ASM. Meta-analyses were conducted separately for each ASM–endpoint–comparator combination, preventing shared controls or repeated participant data from contributing multiple dependent effect estimates to the same pooled analysis.

Statistical heterogeneity was assessed using Cochran’s Q test and the I^2^ statistic [39]. Given the anticipated clinical and methodological heterogeneity across studies, pooled estimates were calculated using a random-effects model based on the DerSimonian and Laird method [40]. Where estimable, 95% prediction intervals were calculated to describe the range of effects that might be expected in a future study. Publication bias was assessed, where applicable, using funnel plots, Begg’s rank correlation test [41], and Egger’s regression test [42]. For outcomes including fewer than 10 studies, funnel plots and formal tests for funnel plot asymmetry were interpreted cautiously due to limited statistical power. For Begg’s and Egger’s tests, statistical significance was defined as *p* < 0.05.

Sensitivity analyses were performed by systematically excluding individual studies to identify potential outliers. Additional sensitivity analyses were conducted according to data type, comparing paired and independent datasets. Sensitivity analyses were also repeated using r = 0.30 and r = 0.80 to assess the robustness of pooled estimates to the assumed pre–post correlation [43,44]. All statistical tests were two-sided. Analyses were performed using Comprehensive Meta-Analysis software, Version 4 (Biostat, Englewood, NJ, USA; CMA4701-532) [45].

### 2.2. Pharmacovigilance Study Using the FAERS Database

#### 2.2.1. Data Extraction and Processing

AE reports submitted to the FAERS database between the first quarter of 2019 and the second quarter of 2026 (2019Q1–2026Q2) were retrieved in August 2026 and analyzed. The Institutional Review Board of Daegu Catholic University exempted this study protocol from review (CUIRB-2023-E007; 9 August 2023). A total of 21 ASMs included in contemporary clinical references for epilepsy management [46] were investigated as the primary suspected (PS) drugs: brivaracetam, carbamazepine, clobazam, clonazepam, eslicarbazepine, ethosuximide, felbamate, gabapentin, lacosamide, lamotrigine, levetiracetam, oxcarbazepine, perampanel, phenobarbital, phenytoin, pregabalin, rufinamide, topiramate, valproate sodium, vigabatrin, and zonisamide. Valproic acid and valproate sodium were combined and analyzed as a single agent, referred to as valproate.

ASM exposure was identified from the DRUG file by matching both the free-text drug name (DRUGNAME) and the active-ingredient field (PROD_AI), each normalized to uppercase with collapsed whitespace, against a predefined list of ingredients and marketed brand names from each of 21 ASMs (Supplementary Table S3).

AEs were analyzed at the preferred term (PT) level using the Medical Dictionary for Regulatory Activities (MedDRA, version 27.0). Male reproductive disorders were identified using the Standardized MedDRA Query (SMQ) ‘Fertility disorders’, which includes 145 narrow-scope PTs covering a broad spectrum of reproductive abnormalities, including infertility, testicular disorders, abnormal sperm parameters, and related reproductive conditions. Three PTs representing nonspecific diagnostic findings and with unclear subject attribution (e.g., chromosome or karyotype abnormalities) were excluded. Of the remaining 142 PTs, 53 PTs are specific to female anatomy or physiology and were additionally excluded given the male-only cohort restriction, yielding 89 PTs (64 male-specific, 25 sex-neutral) for the primary case definition (Supplementary Table S4).

The analysis dataset was constructed using the DEMO file by first removing duplicate reports at the case level, then restricting to male reports. For each CASEID, the record with the highest CASE_VERSION was retained. Ties were resolved using the most recent FDA receipt date, followed by the highest PRIMARYID. This resolved both duplicate submission from case amendments and identical PRIMARYIDs republished across quarterly extracts (*n* = 204). Reports involving the predefined PS drugs and SMQ-related PTs were identified from the DRUG and REAC files, respectively. Reports meeting both criteria were defined as cases for the disproportionality analysis (Supplementary Figure S1). Reports listing more than one drug with role code ‘primary suspect’ were retained under the exposure group of every listed PS ASM, rather than being excluded or assigned to a single drug. Demographic characteristics including patient age, reporting year, and reporter region were extracted from the DEMO file, and clinical outcomes were extracted from the OUTC file.

#### 2.2.2. Data Analysis

FAERS does not provide a defined population denominator; therefore, disproportionality measures were used for signal detection rather than for estimating incidence or risk. Two disproportionality measures, the information component (IC) and reporting odds ratio (ROR), were calculated. The formulas for the IC and ROR were as follows:

(1) $$\mathrm{IC}\;=\;\log_{2}\frac{\left[\frac{A}{A+B}\right]}{\left[\frac{C}{C+D}\right]}$$

(2) $$\mathrm{ROR}=\frac{\left[\frac{A}{B}\right]}{\left[\frac{C}{D}\right]}$$

where

*A*, the number of reports involving both the ASM of interest and ‘Fertility disorders’;

*B*, the number of reports involving the ASM of interest without ‘Fertility disorders’;

*C*, the number of reports involving ‘Fertility disorders’ with all other drugs;

*D*, the number of reports involving all other drugs without ‘Fertility disorders’;

*A* + *B*, the total number of reports involving the ASM of interest;

*C* + *D*, the total number of reports involving all other drugs.

Drug–event pairs with at least three reports were considered eligible for signal detection, consistent with conventional minimum-count criteria used in spontaneous-reporting signal detection [47]. The IC was calculated using a Bayesian confidence propagation neural network with statistical shrinkage [48,49]. An IC signal was defined as IC_025_ > 0, and an ROR signal was defined when the lower bound of the 95% CI exceeded 1 [50,51].

To evaluate the robustness of the valproate signal, two sensitivity analyses were performed. First, the case definition was restricted from the 89 PTs used in the primary analysis (64 male-specific and 25 sex-neutral PTs) to the 64 male-specific PTs only (male-specific-PT sensitivity analysis). Second, an active-comparator sensitivity analysis was performed by comparing valproate with the remaining 20 ASMs rather than with all other FAERS drugs, to examine whether the valproate signal persisted when the comparator was restricted to other ASMs. Because FAERS lacks a defined population denominator, ROR and IC were used for signal detection rather than incidence or risk estimation. Clinical characteristics were summarized using descriptive statistics. All statistical analyses were performed using SAS software, version 9.4 (SAS Institute Inc., Cary, NC, USA), and Microsoft Excel 2021 (Microsoft Corp., Redmond, WA, USA).

## 3. Results

### 3.1. Meta-Analysis of Observational Clinical Studies

#### 3.1.1. Included Studies

**Figure 1:**
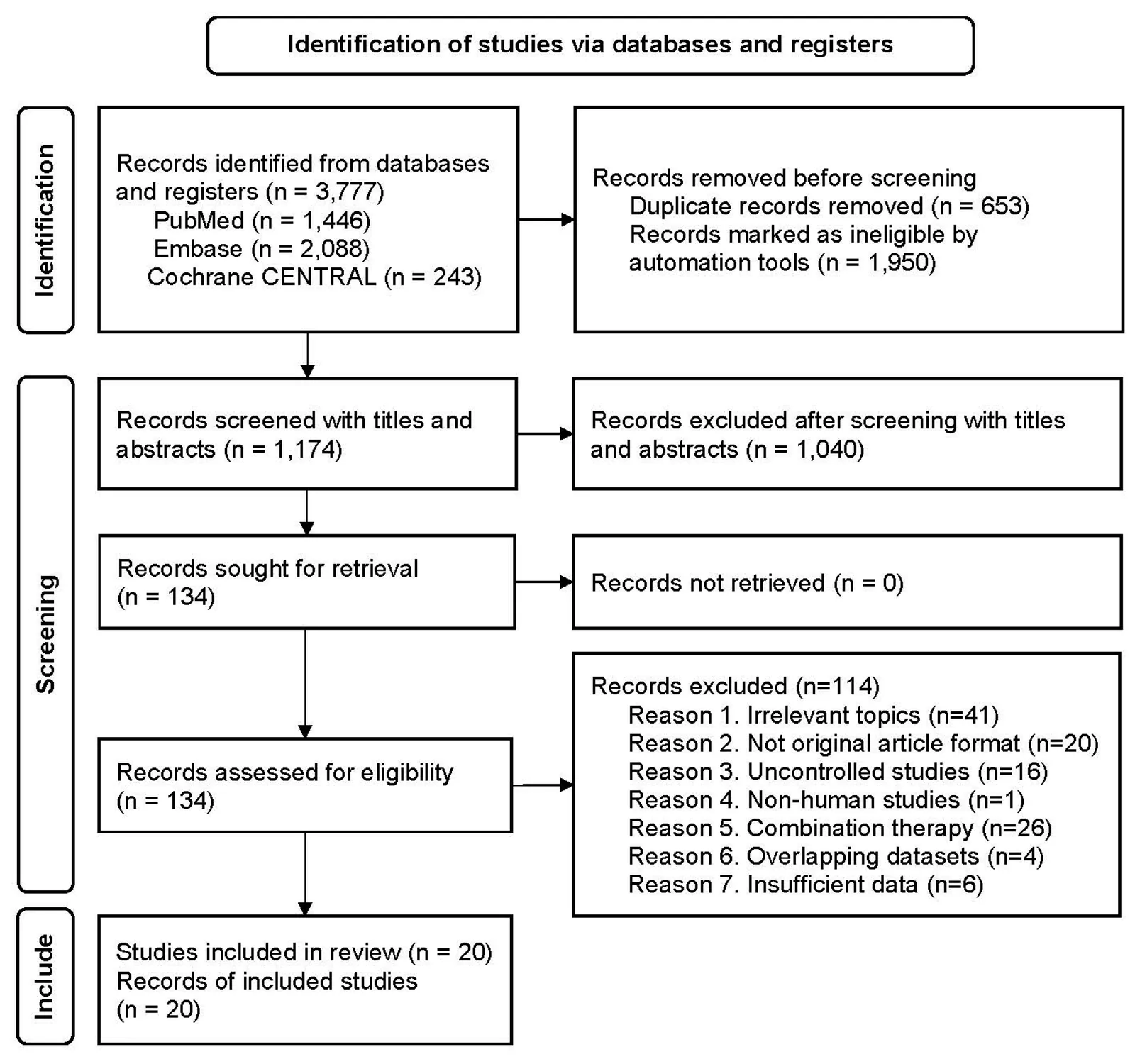
PRISMA flow chart of the study selection process (Meta-analysis).

A total of 3777 records were identified through the database search (Figure 1). After duplicate records and records marked as ineligible by automation tools were removed, 1174 records were screened based on titles and abstracts. Of the 134 full-text articles assessed for eligibility, 114 were excluded according to the predefined criteria. Consequently, 20 studies were included in the final systematic review and meta-analysis [8,9,11,12,13,14,15,16,17,18,19,20,21,22,23,24,25,26,27,28].

#### 3.1.2. Study Characteristics and Quality

The 20 included studies were published between 1990 and 2021 and comprised six before-and-after studies and 14 cross-sectional studies. One study, Isojärvi et al. (1995) [8], included both prospective and cross-sectional components with separate participant groups; these components were analyzed independently where applicable. The total sample size was 1530 participants. The detailed characteristics of the included studies are summarized in Supplementary Table S5.

The studies were conducted across Europe, Asia, the Americas, and Africa. Eighteen studies assessed sex hormones, eight assessed sperm parameters, and three assessed sexual function. All included studies were restricted to ASM monotherapy, and combination therapies were excluded. The analyzed ASMs included valproate (14 studies; 275 subjects), carbamazepine (12 studies; 339 subjects), phenytoin (5 studies; 111 subjects), and levetiracetam (4 studies; 101 subjects).

Among the included studies, 14 cross-sectional studies were assessed using the AXIS tool, with appraisal scores ranging from 14 to 16 (Supplementary Table S6), whereas six before–after studies and a prospective cohort study component were assessed using the adapted Newcastle–Ottawa Scale (NOS), with scores ranging from 5 to 6 (Supplementary Table S7).

#### 3.1.3. Pooled Results

**Figure 2:**
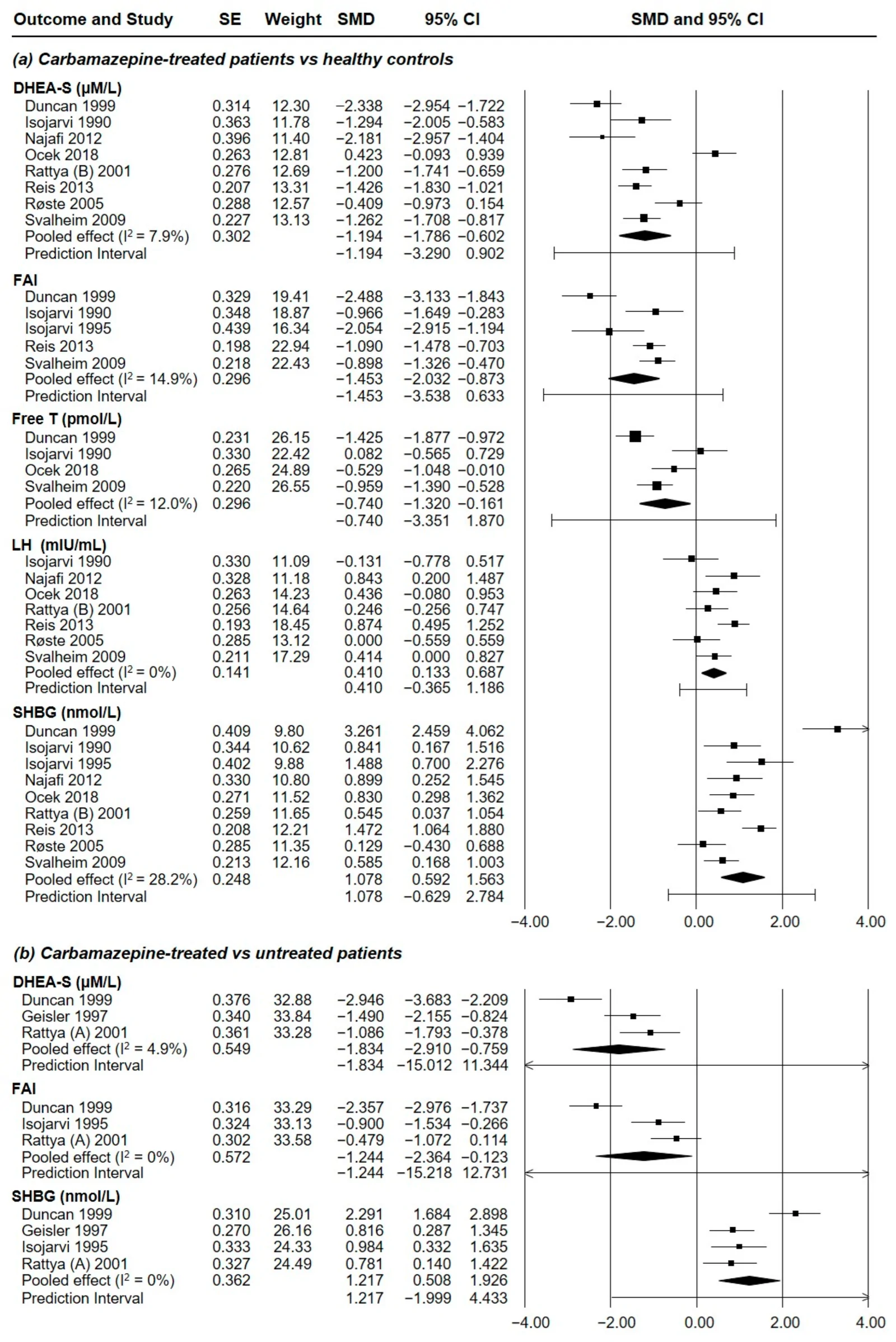
Forest plots of reproductive parameters associated with carbamazepine treatment (Meta-analysis): (**a**) comparison with healthy controls; (**b**) comparison with untreated patients with epilepsy; Duncan 1999 is [21], Isojärvi 1990 is [26], Najafi 2012 is [9], Ocek 2018 is [13], Rättyä (B) 2001 is [19], Reis 2013 is [16], Røste 2005 is [18], Svalheim 2009 is [17], Isojärvi 1995 is [8], Geisler 1997 is [22], and Rättyä (A) 2001 is [20].

**Figure 3:**
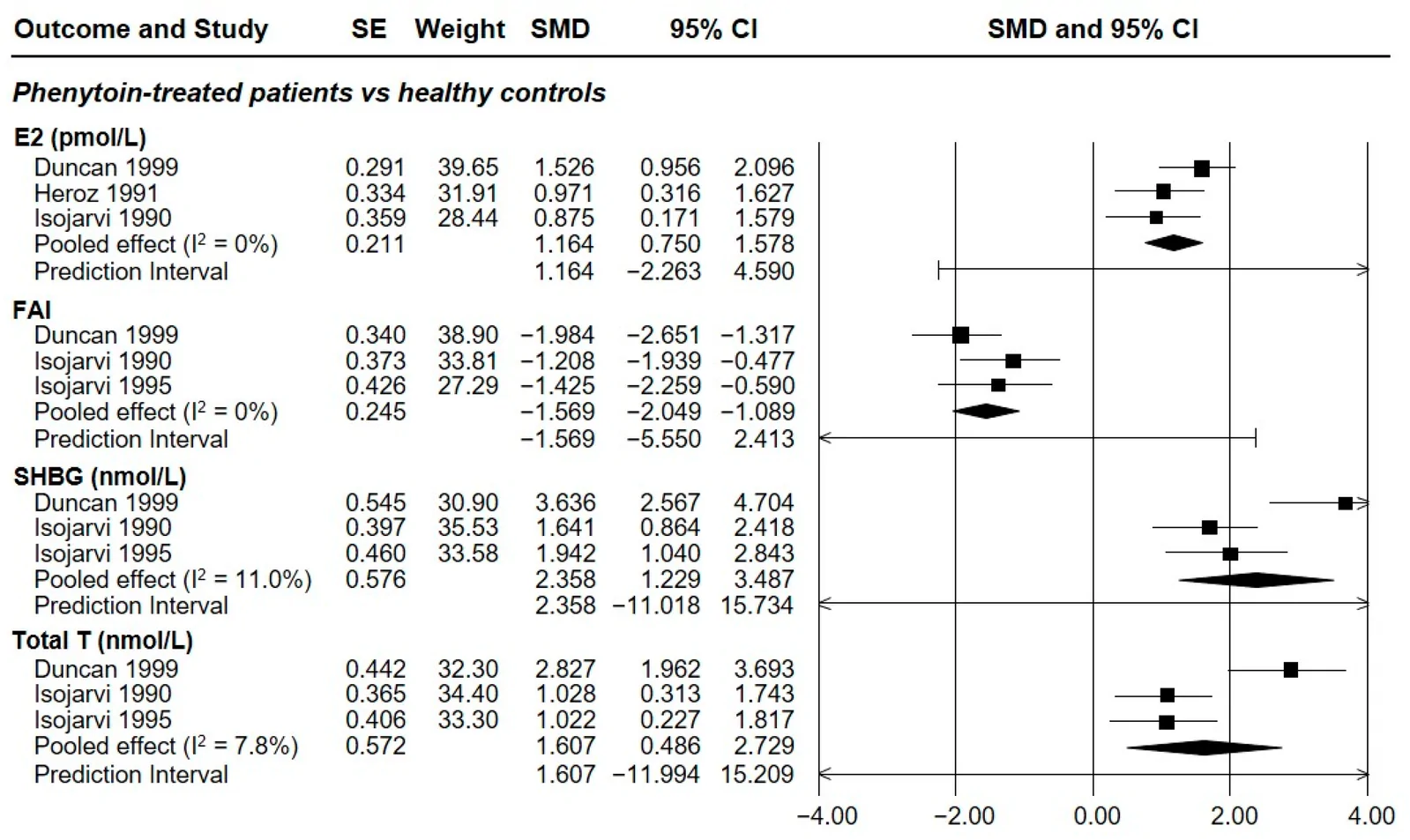
Forest plots of reproductive parameters associated with phenytoin treatment (Meta-analysis): comparison with healthy controls; Duncan 1999 is [21], Herzog 1991 is [25], Isojärvi 1990 is [26], and Isojärvi 1995 is [8].

**Figure 4:**
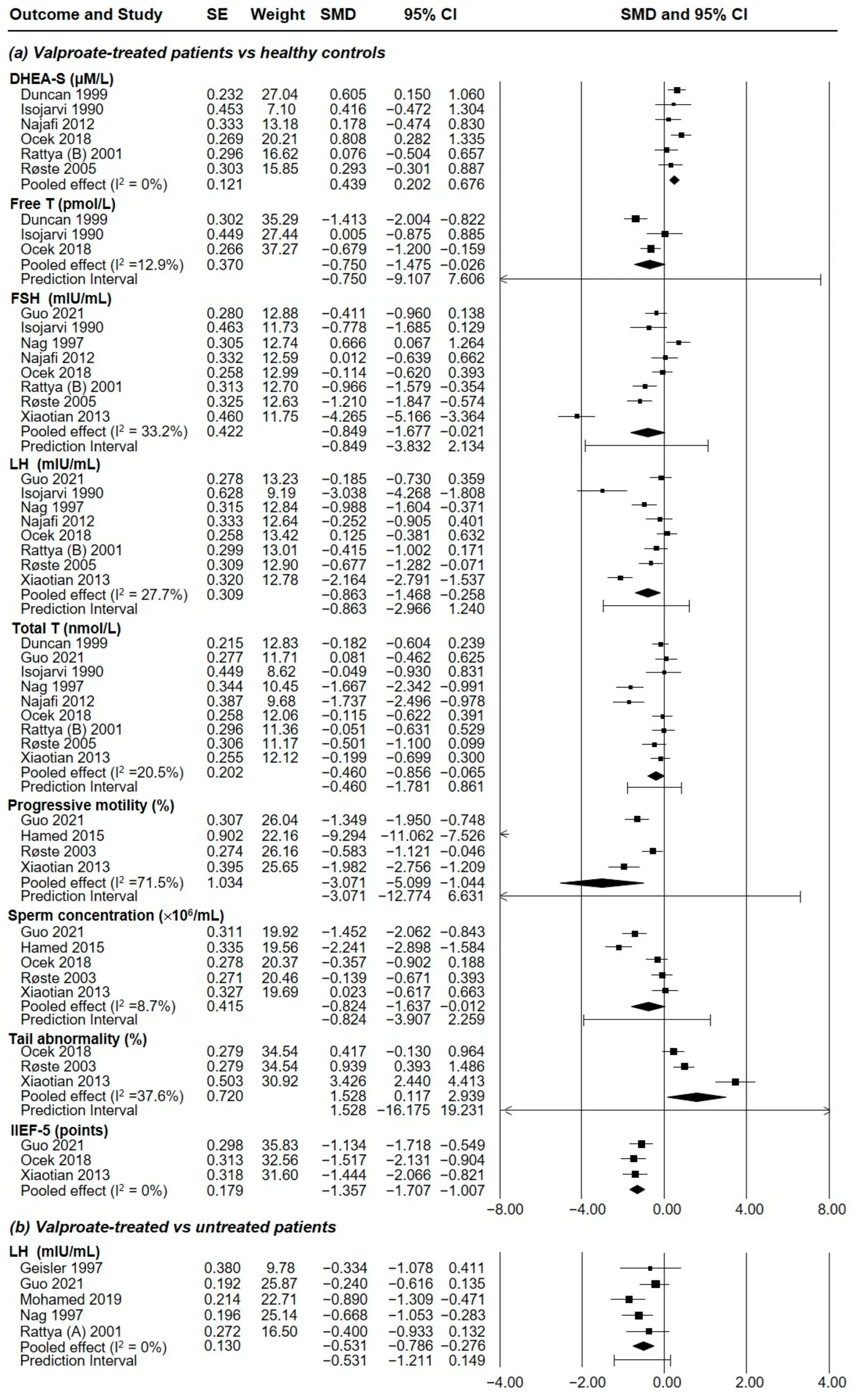
Forest plots of reproductive parameters associated with valproate treatment (Meta-analysis); Duncan 1999 is [21], Guo 2021 is [11], Isojärvi 1990 is [26], Nag 1997 is [23], Najafi 2012 is [9], Ocek 2018 is [13], Rättyä (B) 2001 is [19], Røste 2005 is [18], Xiaotian 2013 is [15], Hamed 2015 is [14], Geisler 1997 is [22], Mohamed 2019 is [12], and Rättyä (A) 2001 is [20]. (**a**) comparison with healthy controls; (**b**) comparison with untreated patients with epilepsy. SE, standard error; SMD, standardized mean difference; CI, confidence interval; DHEA-S, dehydroepiandrosterone sulfate; E2, estradiol; FAI, free androgen index; FSH, follicle-stimulating hormone; IIEF-5, International Index of Erectile Function-5; LH, luteinizing hormone; SHBG, sex hormone-binding globulin; T, testosterone.

Supplementary Tables S8 and S9 summarize the extracted outcome data used in the meta-analysis. The pooled meta-analysis findings are summarized in Supplementary Table S10 and Figure 2, Figure 3 and Figure 4. The pooled estimates indicated that valproate was associated with adverse effects on sex hormones, sperm parameters, and sexual function, whereas carbamazepine and phenytoin were associated primarily with reproductive hormone alterations.

Among patients with epilepsy treated with carbamazepine, significant differences were observed in five sex hormone parameters compared with healthy controls. DHEA-S (SMD −1.194; 95% CI −1.786 to −0.602), FAI (SMD −1.453; 95% CI −2.032 to −0.873), and free T (SMD −0.740; 95% CI −1.320 to −0.161) were lower in carbamazepine-treated patients, whereas LH (SMD 0.410; 95% CI 0.133–0.687) and SHBG (SMD 1.078; 95% CI 0.592–1.563) were higher (Figure 2a). Compared with untreated patients with epilepsy, carbamazepine-treated patients had significantly lower DHEA-S (SMD −1.834; 95% CI −2.910 to −0.759) and FAI (SMD −1.244; 95% CI −2.364 to −0.123), and higher SHBG (SMD 1.217; 95% CI 0.508–1.926) (Figure 2b). No significant differences were observed in sperm parameters between carbamazepine-treated patients and either healthy controls or untreated patients with epilepsy.

For phenytoin, pooled estimates showed significantly higher E2 (SMD 1.164; 95% CI 0.750–1.578), SHBG (SMD 2.358; 95% CI 1.229–3.487), and total T (SMD 1.607; 95% CI 0.486–2.729), and significantly lower FAI (SMD −1.569; 95% CI −2.049 to −1.089) compared with healthy controls (Figure 3). No significant differences in sperm parameters were observed.

For valproate, significant differences were observed across sex hormone, sperm, and sexual function outcomes. Compared with healthy controls, valproate-treated patients had significantly higher DHEA-S (SMD 0.439; 95% CI 0.202–0.676) and significantly lower free T (SMD −0.750; 95% CI −1.475 to −0.026), FSH (SMD −0.849; 95% CI −1.677 to −0.021), LH (SMD −0.863; 95% CI −1.468 to −0.258), and total T (SMD −0.460; 95% CI −0.856 to −0.065). Significant differences were also observed in sperm parameters, including lower progressive motility (SMD −3.071; 95% CI −5.099 to −1.044), lower sperm concentration (SMD −0.824; 95% CI −1.637 to −0.012), and higher total sperm abnormalities (SMD 1.528; 95% CI 0.117–2.939). In addition, the IIEF-5 score decreased, with an SMD of −1.357 (95% CI −1.707 to −1.007) (Figure 4a). Compared with untreated patients with epilepsy, valproate-treated patients had significantly lower LH levels (SMD −0.531; 95% CI −0.786 to −0.276) (Figure 4b).

Prediction intervals were generally wider than the corresponding confidence intervals and crossed the null for several outcomes, indicating substantial uncertainty in the effect that might be observed in a future study. In particular, substantial heterogeneity was observed for some valproate semen outcomes, including progressive motility (I^2^ = 71.5%). However, in leave-one-out sensitivity analyses, the direction and statistical significance of the progressive-motility estimate remained unchanged after exclusion of individual studies, suggesting that the finding was not driven by a single study (Supplementary Table S13). Endpoint-specific prediction intervals are presented in Supplementary Table S10. Dose-stratified analyses were not performed because daily dose and treatment duration were inconsistently reported across the included studies.

No evidence of publication bias was detected in the assessed outcomes. Sensitivity analyses for the remaining outcomes were generally consistent with the primary analyses (Supplementary Tables S11–S14).

### 3.2. Pharmacovigilance Study Using the FAERS Database

#### 3.2.1. Demographic Characteristics

The pharmacovigilance analysis followed the data-processing flow outlined in Supplementary Figure S1. After deduplication and restriction to male reports, the analysis cohort comprised 3,672,690 reports. Of these, 3,671,745 involved any drug coded as a primary suspect, including 64,126 involving one of the 21 ASMs of interest; 10,257,773 adverse-event mentions were recorded across the cohort, of which 2956 reports involved a qualifying narrow-scope SMQ ‘Fertility disorders’ PT. Ultimately, 47 reports involving both an ASM as the PS drug and a qualifying PT were included in the disproportionality analysis. The demographic characteristics of these 47 reports are summarized in Supplementary Table S15. The mean patient age was 41.7 years (among 27 reports with age recorded). Most reports originated from Europe (59.6%), followed by North America (34.0%), South America (4.3%), and Asia (2.1%), indicating limited representation of Asian populations.

#### 3.2.2. Disproportionality Analysis

Supplementary Table S16 presents the disproportionality analysis results for male reproductive disorders associated with individual ASMs. The reported PTs included infertility, testicular disorder, testicular atrophy, decreased sperm concentration, azoospermia, and other related male reproductive abnormalities (Supplementary Table S16). Valproate (*n* = 21), gabapentin (*n* = 9), levetiracetam (*n* = 8), and lamotrigine (*n* = 3) each had at least three reports.

**Figure 5:**
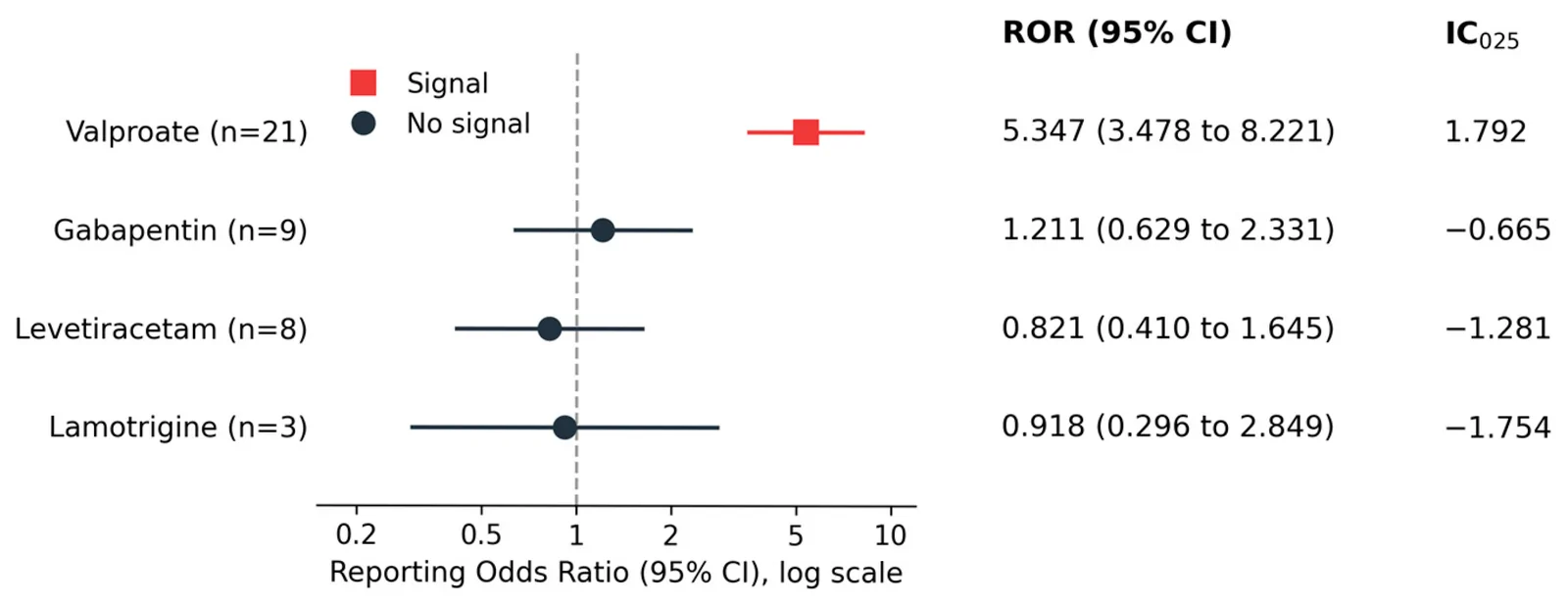
Forest plot of reporting odds ratios (RORs) with 95% confidence intervals and information component lower credibility bounds (IC_025_) for at least three male reproductive disorder reports in FAERS (2019Q1–2026Q2).

Only valproate showed a significant signal for male reproductive disorders (ROR 5.347; 95% CI 3.478–8.221; IC_025_ 1.792). A total of 21 reports were associated with valproate, most commonly involving testicular disorder (*n* = 5), testicular atrophy (*n* = 5), infertility (*n* = 4), sperm concentration decreased (*n* = 3), and azoospermia (*n* = 3), with spermatozoa morphology abnormal (*n* = 2) and spermatozoa progressive motility decreased (*n* = 2), and single reports of varicocele, oligoasthenozoospermia, hypogonadism, hypogonadism male, secondary hypogonadism, teratospermia, genital atrophy, and semen viscosity decreased. In the male-specific-PT sensitivity analysis, the valproate signal was materially unchanged (ROR 6.181, 95% CI 3.776–10.118; IC_025_ 1.911; see Supplementary Table S4 footnote). In the active-comparator sensitivity analysis using the remaining 20 ASMs as the comparator group, the valproate signal persisted (ROR 9.746, 95% CI 5.480–17.334; IC_025_ 2.083). Detailed disproportionality analysis results for all 21 ASMs are provided in Supplementary Table S16 and summarized in Figure 5. Among these, four ASMs—valproate, gabapentin, levetiracetam, and lamotrigine—met the prespecified eligibility threshold of at least three reports for signal detection, and their ROR and IC_025_ estimates are summarized in Figure 5.

## 4. Discussion

This study identified distinct patterns of male reproductive outcomes across individual ASMs by examining controlled clinical evidence alongside spontaneous pharmacovigilance reports. Previous quantitative syntheses have generally focused on a single ASM, such as valproate [52], or on specific reproductive domains, such as endogenous sex hormones [53], while previous FAERS analyses have primarily examined male infertility [4]. In contrast, the present study evaluated multiple individual ASMs across reproductive hormones, semen parameters, and sexual function, incorporated both healthy controls and untreated patients with epilepsy where data permitted, and complemented these clinical data with a broader SMQ-based FAERS assessment. In the meta-analysis, valproate was associated with alterations across multiple reproductive domains, whereas carbamazepine and phenytoin were predominantly associated with endocrine changes. In the FAERS analysis, valproate showed a disproportionate reporting signal for male reproductive disorders. Considered together, these findings provide complementary clinical and pharmacovigilance perspectives and suggest that male reproductive effects may vary across individual ASMs rather than represent a uniform class effect. This drug-specific pattern highlights the clinical relevance of considering reproductive health when selecting and monitoring long-term ASM therapy in men.

Valproate was associated with alterations across multiple reproductive domains in the meta-analysis. Compared with healthy controls, valproate-treated patients showed alterations in reproductive hormone levels, reduced progressive sperm motility and sperm concentration, increased sperm abnormalities, and lower IIEF-5 scores. These findings extend prior valproate-focused quantitative evidence by incorporating hormonal and sexual-function outcomes alongside semen parameters in multi-ASM assessment [52]. In addition, LH levels were lower in valproate-treated patients than in untreated patients with epilepsy, suggesting that this association may not be fully explained by epilepsy itself, although residual confounding cannot be excluded.

The FAERS analysis provided an additional pharmacovigilance perspective on valproate. Among the ASMs evaluated, valproate showed a disproportionate reporting signal for male reproductive disorders (ROR 5.347; 95% CI 3.478–8.221; IC_025_ 1.792). A previous FAERS-based study identified a signal for valproate using the MedDRA PT “infertility male” [4]. The present analysis used the SMQ “Fertility disorders”, encompassing a broader range of reproductive events, including male infertility, testicular disorders, and sperm-related abnormalities. This approach expanded the pharmacovigilance assessment beyond male infertility alone, although the limited number of reports and the inherent limitations of spontaneous reporting warrant cautious interpretation of the signal.

The mechanisms underlying valproate-associated reproductive effects are likely multifactorial. In addition to dysregulation of the hypothalamic–pituitary–gonadal axis, valproate may exert direct testicular effects through oxidative stress, mitochondrial dysfunction, and carnitine depletion, potentially impairing steroidogenesis, spermatogenesis, and sperm energy metabolism [18,19,52,54,55,56,57,58,59]. Valproate also acts as a histone deacetylase inhibitor, and experimental studies have demonstrated testicular histone hyperacetylation and altered sperm DNA methylation, suggesting an additional epigenetic mechanism affecting spermatogenesis [60]. Together, these endocrine, metabolic, oxidative, and epigenetic pathways may contribute to the broader pattern of hormonal, semen, and sexual-function alterations observed with valproate. However, much of the mechanistic evidence remains preclinical, and its relevance and relative contribution in men remain uncertain.

Carbamazepine and phenytoin were mainly associated with alterations in endocrine parameters. For carbamazepine, the meta-analysis showed significant differences with lower DHEA-S (SMD −1.834; 95% CI −2.910 to −0.759) and FAI (SMD −1.244; 95% CI −2.364 to −0.123), and higher SHBG (SMD 1.217; 95% CI 0.508–1.926) compared with untreated patients with epilepsy. These findings suggest that the endocrine alterations observed with carbamazepine may not be fully attributable to epilepsy itself, although residual confounding cannot be excluded [12,13]. These alterations may partly reflect the enzyme-inducing properties of carbamazepine [61], which can increase hepatic SHBG synthesis and aromatase activity—an enzyme that converts T into E2—resulting in lower free T and FAI [7,8,25]. Reduced DHEA-S levels may also reflect decreased adrenal production or accelerated hepatic and peripheral clearance induced by carbamazepine-mediated enzyme induction [22,61]. For phenytoin, pooled estimates showed a similar endocrine pattern, with higher E2 (SMD 1.164; 95% CI 0.750–1.578), SHBG (SMD 2.358; 95% CI 1.229–3.487), and total T (SMD 1.607; 95% CI 0.486–2.729), and significantly lower FAI (SMD −1.569; 95% CI −2.049 to −1.089) compared with healthy controls. These hormonal changes are also likely related to its enzyme-inducing properties [25]. This predominantly endocrine pattern is consistent with the hepatic enzyme-inducing effects of carbamazepine and phenytoin, which increase SHBG and alter sex-steroid disposition, thereby reducing androgen bioavailability despite preserved or increased total testosterone concentrations [19,20,25,61]. In contrast, valproate may influence reproductive function through a broader combination of endocrine, oxidative, mitochondrial, metabolic, and direct testicular pathways. These mechanistic differences may partly explain the broader reproductive phenotype observed with valproate in the present meta-analysis. However, this distinction should be interpreted cautiously, as sperm abnormalities have also been reported with carbamazepine and phenytoin and the available semen data remain limited.

Unlike valproate, carbamazepine and phenytoin did not show significant disproportionality signals in the FAERS analysis. The absence of corresponding FAERS signals may reflect limited reporting, declining use of these older ASMs, or differences between laboratory-detected endocrine outcomes and spontaneously reported clinical events. Accordingly, the clinical and pharmacovigilance findings should not be interpreted as directly concordant.

The findings from the meta-analysis and FAERS analysis can be further interpreted in the context of FDA-approved labeling summarized in Supplementary Table S17. For valproate, as the most widely used and studied ASM, labeling information broadly describes male reproductive toxicity, although sexual dysfunction is not specifically mentioned [62]. For carbamazepine, the FDA-approved label describes testicular atrophy and aspermatogenesis in rats but states that the relevance of these findings to humans is unclear [63]. Several clinical studies, however, have reported sperm-related abnormalities in men exposed to carbamazepine, including abnormal morphology, reduced motility, decreased sperm count, and reduced testicular volume [27,64]. For phenytoin, the FDA-approved label indicates that fertility effects have not been adequately assessed [65], whereas our meta-analysis identified significant alterations in several sex hormone parameters. These comparisons provide useful regulatory context and suggest that some male reproductive outcomes reported in clinical studies, particularly hormonal and sexual function outcomes, may not be fully reflected in current labeling language.

The absence of significant FAERS signals for carbamazepine, phenytoin, and other ASMs should be interpreted cautiously. Male reproductive disorders, including infertility and hormonal abnormalities, are often asymptomatic and may remain unrecognized until specialized evaluations are performed. Consequently, these outcomes are less likely to be spontaneously reported than acute or clinically apparent adverse events. In addition, pharmacovigilance databases are subject to underreporting, confounding, and limited clinical detail. Consistent with these limitations, only four ASMs—valproate (*n* = 21), gabapentin (*n* = 9), levetiracetam (*n* = 8), and lamotrigine (*n* = 3)—had three or more male reproductive disorder-related AE reports in the 2019Q1–2026Q2 extraction (Supplementary Table S16). This sparsity may reduce the precision and stability of ROR and IC estimates, particularly for ASMs near the minimum reporting threshold, consistent with concerns raised in recent FAERS-based pharmacovigilance studies [66] and methodological concerns regarding disproportionality analyses in small databases or subsets [67]. Older enzyme-inducing ASMs such as carbamazepine and phenytoin had fewer reports, which may partly reflect their declining clinical use [68] and may have constrained the scope of the pharmacovigilance analysis. Several ASMs, including levetiracetam, had three or more FAERS reports but did not meet the predefined criteria for significant disproportionality signals. Evidence for levetiracetam, lamotrigine, and other newer ASMs remains limited in both analyses. In the clinical dataset, levetiracetam was represented by only four studies involving 101 participants, while data for lamotrigine and other newer ASMs was insufficient for quantitative synthesis. Similarly, FAERS contained few reproductive reports for these agents, limiting the precision of drug-specific signal estimates. Accordingly, the absence of significant findings or disproportionality signals should not be interpreted as evidence of reproductive safety. Adequately powered prospective studies across individual ASMs, including newer agents for which evidence remains particularly limited, are needed in men of reproductive age. Such studies should incorporate standardized assessments of reproductive hormones, semen parameters, and sexual function, together with detailed information on daily dose, cumulative exposure, and treatment duration, to better characterize potential dose–response relationships.

This study has several limitations. First, the meta-analysis was based on observational studies with heterogeneous designs, populations, and reproductive outcome measures. Although untreated patients with epilepsy were used as comparators where available, seizure frequency, disease duration, epilepsy severity, and related neuroendocrine disturbances were inconsistently reported and could not be fully accounted for. In addition, direct quantitative comparisons between untreated patients with epilepsy and healthy controls were not feasible because of insufficient comparable data across reproductive outcomes. Therefore, the independent contribution of epilepsy itself could not be fully assessed, and residual confounding from the underlying epilepsy and other clinical factors, including comorbidities such as obesity, diabetes mellitus, or varicocele, lifestyle factors, and concomitant medications, cannot be excluded. Second, despite the overall pooled sample of 1530 participants, the number of contributing studies and participants was limited for certain ASMs and specific reproductive endpoints, restricting the precision of some pooled estimates. Moreover, prediction intervals were wide and crossed the null for several pooled outcomes, indicating substantial uncertainty regarding the effect that might be observed in future studies. Therefore, statistically significant pooled estimates should be interpreted cautiously, particularly when based on a small number of studies or accompanied by substantial between-study heterogeneity. For valproate-related semen outcomes, such heterogeneity may reflect differences across studies in geographic settings, study design, drug dose, and treatment duration; however, these potential sources could not be formally examined because relevant study-level data were insufficient. Although leave-one-out sensitivity analyses showed that the direction and statistical significance of the progressive-motility estimate remained unchanged after exclusion of individual studies, the substantial heterogeneity indicates that the magnitude of the association remains uncertain. As the meta-analysis relied on the available original studies, much of the evidence was derived from older research, highlighting the need for contemporary studies to provide more robust and current evidence. Third, FAERS is a spontaneous reporting system that cannot establish causality or provide incidence estimates and is subject to reporting bias, with clinically conspicuous reproductive events potentially more likely to be reported than mild or subclinical abnormalities. In addition, the small number of reproductive adverse event reports may limit the precision and stability of ROR and IC estimates, particularly for individual ASMs [67]. Age- and dose-stratified analyses and adjustment for concomitant medications were not performed because of incomplete reporting and the small number of qualifying cases, and potential confounding by indication, epilepsy-related characteristics, concomitant medications, and other clinical factors could not be adequately controlled. Furthermore, dose–response relationships could not be evaluated because daily dose and treatment duration were inconsistently reported in the clinical studies and incompletely documented in FAERS. Finally, the geographic distribution of FAERS reports was heavily skewed toward Europe (59.6%) and North America (34.0%), with minimal representation from Asia (2.1%) and South America (4.3%); therefore, these findings should be interpreted with caution and may not generalize to male populations with epilepsy in other geographic regions.

Despite these limitations, this study has several strengths. First, unlike previous quantitative syntheses focused on individual ASMs or specific reproductive domains, this study evaluated multiple individual ASMs across reproductive hormones, semen parameters, and sexual function within a common analytical framework. Second, where data permitted, the use of both healthy controls and untreated patients with epilepsy provided additional context for distinguishing ASM-associated alterations from reproductive dysfunction related to epilepsy itself, which may independently affect reproductive and sexual function through neuroendocrine dysregulation [69,70]. Nevertheless, residual confounding cannot be excluded. Third, the FAERS analysis broadened the scope of the safety assessment by capturing a wider range of spontaneously reported reproductive events using the SMQ “Fertility disorders,” providing a complementary perspective with outcomes and inferential limitations distinct from those of the meta-analysis. Finally, examining controlled clinical evidence and pharmacovigilance signals within the same study enabled a broader characterization of drug-specific male reproductive safety while preserving the distinct evidentiary roles of the two data sources. This approach is consistent with previous drug-safety research emphasizing the value of interpreting pharmacovigilance signals alongside independent sources of evidence [71,72].

Clinically, reproductive health should be considered when selecting or reviewing long-term ASM therapy in men of reproductive age, particularly those planning to conceive or reporting reproductive or sexual problems. When clinically indicated, targeted assessment of semen parameters, reproductive hormones, or sexual function may be considered. Limited clinical evidence suggests that some ASM-associated reproductive changes may improve after treatment withdrawal or modification, including reproductive endocrine changes after carbamazepine withdrawal and semen abnormalities after valproate discontinuation or switching [73,74]. If ASM-related reproductive effects are suspected, the treatment regimen should be reviewed while maintaining adequate seizure control.

## 5. Conclusions

In conclusion, male reproductive effects were not uniform across ASMs but exhibited distinct drug-specific patterns. Valproate was associated with alterations across multiple reproductive domains, whereas carbamazepine and phenytoin were primarily associated with endocrine alterations. These findings support consideration of reproductive health when selecting and monitoring long-term ASM therapy in men. Future prospective studies incorporating standardized assessments of semen parameters, reproductive hormones, sexual function, and detailed ASM exposure information would help clarify the causality, reversibility, and long-term clinical relevance of ASM-associated male reproductive effects.

## Data Availability

All data supporting the findings of this systematic review and meta-analysis are derived from the published studies cited in the article. The FAERS data analyzed in this study are publicly available from the U.S. Food and Drug Administration (FDA) Adverse Event Reporting System.

## Supplementary Materials

The following supporting information can be downloaded at: https://www.mdpi.com/article/10.3390/jcm15197566/s1, Figure S1: Flow diagram of pharmacovigilance assessment of male reproductive disorder associated with ASMs (FAERS analysis); Table S1. PRISMA 2020 checklist; Table S2. The detailed search strategy for each database (Meta-analysis); Table S3. Drug-name mapping for the 21 antiseizure medications (FAERS analysis); Table S4. Definition of reproductive disorders based on the MedDRA narrow-scope SMQ ‘Fertility disorders’ (version 27.0) (FAERS analysis); Table S5. Characteristics of included studies (Meta-analysis); Table S6. Quality assessment of cross-sectional studies using the AXIS tool (Meta-analysis); Table S7. Quality assessment of before–after and prospective cohort studies using an adapted NOS tool (Meta-analysis); Table S8. Data extracted: before–after studies and a prospective cohort study (Meta-analysis); Table S9. Data extracted: cross-sectional studies (Meta-analysis); Table S10. Findings on the association between antiseizure medication use and male reproductive disorders via pharmacovigilance and meta-analysis; Table S11. Sensitivity analysis of carbamazepine (Meta-analysis); Table S12. Sensitivity analysis of phenytoin (Meta-analysis); Table S13. Sensitivity analysis of valproate (Meta-analysis); Table S14. Sensitivity analysis of alternative correlation coefficients (Meta-analysis); Table S15. Demographic characteristics of patients with AED-associated male reproductive disorders (FAERS analysis); Table S16. The primary suspected antiseizure medications and disproportionality analysis results for male reproductive disorders (FAERS analysis); Table S17. Comparative overview of regulatory approvals by country.

## Abbreviations

The following abbreviations are used in this manuscript:

AE	Adverse event
ASM	Antiseizure medication
AXIS	Appraisal tool for cross-sectional studies
CIs	Confidence intervals
DHEA-S	Dehydroepiandrosterone sulfate
E2	Estradiol
FAERS	FDA Adverse Event Reporting System
FAI	Free androgen index
FDA	Food and Drug Administration
FSH	Follicle-stimulating hormone
GABA	Gamma-aminobutyric acid
IC	Information component
IIEF-5	International Index of Erectile Function-5
LH	Luteinizing hormone
MedDRA	Medical Dictionary for Regulatory Activities
PRISMA	Preferred Reporting Items for Systematic Reviews and Meta-Analyses
PRL	Prolactin
PS	Primary suspected
PTs	Preferred terms
ROR	Reporting odds ratio
RWD	Real-world data
SD	Standard deviation
SHBG	Sex hormone-binding globulin
SMD	Standardized mean difference
SMQ	Standardized MedDRA queries
T	Testosterone
