# Supplementary material for: Male Reproductive Disorders Associated with Antiseizure Medications: Evidence from Meta-Analysis and FAERS Pharmacovigilance Study
Source: J Clin Med. 2026 Sep 29;15(19):7566. doi: 10.3390/jcm15197566 (PMC13666318; doi:10.3390/jcm15197566)
Supplement: Supplementary file 1 [file jcm-15-07566-s001.zip › Supplementary tables_JCM_revised_final.pdf]

Table S1. PRISMA 2020 checklist

Section and Topic	Item #	Checklist item	Location where item is reported
**TITLE**			
Title	1	Identify the report as a systematic review.	p. 1 (title identifies the meta-analysis component); p. 3 (systematic review explicitly stated).
**ABSTRACT**			
Abstract	2	See the PRISMA 2020 for Abstracts checklist.	p. 1, Abstract.
**INTRODUCTION**			
Rationale	3	Describe the rationale for the review in the context of existing knowledge.	pp. 2–3, Introduction.
Objectives	4	Provide an explicit statement of the objective(s) or question(s) the review addresses.	p. 3, Introduction (final paragraph).
**METHODS**			
Eligibility criteria	5	Specify the inclusion and exclusion criteria for the review and how studies were grouped for the syntheses.	pp. 3–4, Sections 2.1.2 and 2.1.4.
Information sources	6	Specify all databases, registers, websites, organisations, reference lists and other sources searched or consulted to identify studies. Specify the date when each source was last searched or consulted.	p. 3, Section 2.1.1.
Search strategy	7	Present the full search strategies for all databases, registers and websites, including any filters and limits used.	p. 3, Section 2.1.1; Supplementary Table S1.
Selection process	8	Specify the methods used to decide whether a study met the inclusion criteria of the review, including how many reviewers screened each record and each report retrieved, whether they worked independently, and if applicable, details of automation tools used in the process.	p. 3, Section 2.1.2.
Data collection process	9	Specify the methods used to collect data from reports, including how many reviewers collected data from each report, whether they worked independently, any processes for obtaining or confirming data from study investigators, and if applicable, details of automation tools used in the process.	pp. 3–4, Section 2.1.3.
Data items	10a	List and define all outcomes for which data were sought. Specify whether all results that were compatible with each outcome domain in each study were sought (e.g. for all measures, time points, analyses), and if not, the methods used to decide which results to collect.	pp. 3–4, Section 2.1.3.
	10b	List and define all other variables for which data were sought (e.g. participant and intervention characteristics, funding sources). Describe any assumptions made about any missing or unclear information.	pp. 3–4, Section 2.1.3 (assumptions for unavailable SDs and the pre–post correlation).
Study risk of bias assessment	11	Specify the methods used to assess risk of bias in the included studies, including details of the tool(s) used, how many reviewers assessed each study and whether they worked independently, and if applicable, details of automation tools used in the process.	pp. 3–4, Section 2.1.3; Supplementary Table S4.
Effect measures	12	Specify for each outcome the effect measure(s) (e.g. risk ratio, mean difference) used in the synthesis or presentation of results.	p. 4, Section 2.1.4.

Synthesis methods	13a	Describe the processes used to decide which studies were eligible for each synthesis (e.g. tabulating the study intervention characteristics and comparing against the planned groups for each synthesis (item #5)).	p. 4, Section 2.1.4 (≥3 studies per endpoint; stratification by control group type and individual ASM).
	13b	Describe any methods required to prepare the data for presentation or synthesis, such as handling of missing summary statistics, or data conversions.	pp. 3–4, Section 2.1.3 (SD derivation and assumed pre–post correlation).
	13c	Describe any methods used to tabulate or visually display results of individual studies and syntheses.	pp. 5–11, Table 1 and Figure 2; Supplementary Tables S5–S6.
	13d	Describe any methods used to synthesize results and provide a rationale for the choice(s). If meta-analysis was performed, describe the model(s), method(s) to identify the presence and extent of statistical heterogeneity, and software package(s) used.	p. 4, Section 2.1.4.
	13e	Describe any methods used to explore possible causes of heterogeneity among study results (e.g. subgroup analysis, meta-regression).	p. 4, Section 2.1.4 (stratified by control group type and individual ASM).
	13f	Describe any sensitivity analyses conducted to assess robustness of the synthesized results.	p. 4, Section 2.1.4.
Reporting bias assessment	14	Describe any methods used to assess risk of bias due to missing results in a synthesis (arising from reporting biases).	p. 4, Section 2.1.4.
Certainty assessment	15	Describe any methods used to assess certainty (or confidence) in the body of evidence for an outcome.	*Not assessed in the manuscript.*
Study selection	16a	Describe the results of the search and selection process, from the number of records identified in the search to the number of studies included in the review, ideally using a flow diagram.	pp. 5–6, Section 3.1.1; Figure 1 (p. 8).
	16b	Cite studies that might appear to meet the inclusion criteria, but which were excluded, and explain why they were excluded.	Figure 1 (p. 6) gives aggregate exclusion reasons; individual near-eligible excluded reports are not listed.
Study characteristics	17	Cite each included study and present its characteristics.	p. 6, Section 3.1.2; Supplementary Table S3.
Risk of bias in studies	18	Present assessments of risk of bias for each included study.	p. 6, Section 3.1.2; Supplementary Table S4.
Results of individual studies	19	For all outcomes, present, for each study: (a) summary statistics for each group (where appropriate) and (b) an effect estimate and its precision (e.g. confidence/credible interval), ideally using structured tables or plots.	pp. 5–11, Section 3.1.3 and Figure 2; Supplementary Tables S5–S6.
Results of syntheses	20a	For each synthesis, briefly summarise the characteristics and risk of bias among contributing studies.	p. 6, Section 3.1.2; Supplementary Tables S3–S4.
	20b	Present results of all statistical syntheses conducted. If meta-analysis was done, present for each the summary estimate and its precision (e.g. confidence/credible interval) and measures of statistical heterogeneity. If comparing groups, describe the direction of the effect.	pp. 5–11, Section 3.1.3, Table 1, and Figure 2.
	20c	Present results of all investigations of possible causes of heterogeneity among study results.	pp. 5–11 (results stratified by individual ASM and control group); Figure 2.

	20d	Present results of all sensitivity analyses conducted to assess the robustness of the synthesized results.	pp. 6-7, Section 3.1.3; Supplementary Tables S7–S9.
Reporting biases	21	Present assessments of risk of bias due to missing results (arising from reporting biases) for each synthesis assessed.	pp. 6-7, Section 3.1.3.
Certainty of evidence	22	Present assessments of certainty (or confidence) in the body of evidence for each outcome assessed.	*Not assessed in the manuscript.*
**DISCUSSION**			
Discussion	23a	Provide a general interpretation of the results in the context of other evidence.	pp. 11–14, Discussion.
	23b	Discuss any limitations of the evidence included in the review.	pp. 13–14, Discussion (limitations of the included evidence).
	23c	Discuss any limitations of the review processes used.	pp. 13–14, Discussion (review-process-specific limitations are not separately reported).
	23d	Discuss implications of the results for practice, policy, and future research.	p. 14, Discussion and Section 5, Conclusions.
**OTHER INFORMA TION**			
Registration and protocol	24a	Provide registration information for the review, including register name and registration number, or state that the review was not registered.	p. 3, Section 2.1.1 (PROSPERO CRD42024602937).
	24b	Indicate where the review protocol can be accessed, or state that a protocol was not prepared.	p. 3, Section 2.1.1 (PROSPERO record CRD42024602937).
	24c	Describe and explain any amendments to information provided at registration or in the protocol.	*Not applicable.*
Support	25	Describe sources of financial or non-financial support for the review, and the role of the funders or sponsors in the review.	p. 15, Funding.
Competing interests	26	Declare any competing interests of review authors.	p. 15, Conflicts of Interest.
Availability of data, code and other materials	27	Report which of the following are publicly available and where they can be found: template data collection forms; data extracted from included studies; data used for all analyses; analytic code; any other materials used in the review.	p. 15, Data Availability Statement; Supplementary Tables S5–S6.

From: Page MJ, McKenzie JE, Bossuyt PM, Boutron I, Hoffmann TC, Mulrow CD, et al. The PRISMA 2020 statement: an updated guideline for reporting systematic reviews. BMJ 2021;372:n71. doi: 10.1136/bmj.n71 For more information, visit: http://www.prisma-statement.org/

Table S2. The detailed search strategy for each database (Meta-analysis)

Database	Search strategy
**Pubmed**	(((((((((((((((((((((((((((((((((carbamazepine[MeSH Terms]) OR (anticonvulsants[MeSH Terms])) OR (anticonvulsant*[Title/Abstract])) OR (antiepilep*[Title/Abstract])) OR (epilepsy[Title/Abstract])) OR (epilep*[Title/Abstract])) OR (AED*[Title/Abstract])) OR (epilepsy[MeSH Terms])) OR (phenytoin[MeSH Terms])) OR (gabapentin[MeSH Terms])) OR (valproic acid[MeSH Terms])) OR (valproate[Title/Abstract])) OR (valproic acid[Title/Abstract])) OR (Sodium valproate[Title/Abstract])) OR (oxcarbazepine[MeSH Terms])) OR (lamotrigine[MeSH Terms])) OR (topiramate[MeSH Terms])) OR (levetiracetam[MeSH Terms])) OR (pregabalin[MeSH Terms])) OR (clonazepam[MeSH Terms])) OR (ethosuximide[MeSH Terms])) OR (phenobarbital[MeSH Terms])) OR (felbamate[MeSH Terms])) OR (zonisamide[MeSH Terms])) OR (brivaracetam[Title/Abstract])) OR (eslicarbazepine[Title/Abstract])) OR (clobazam[MeSH Terms])) OR (lacosamide[MeSH Terms])) OR (vigabatrin[MeSH Terms])) OR (perampanel[Title/Abstract])) OR (rufinamide[Title/Abstract])) AND (((((((((((((((((((((((((((sperm count[MeSH Terms]) OR (sperm motility[MeSH Terms])) OR (spermatozoa[MeSH Terms])) OR (sperm head[MeSH Terms])) OR (sperm midpiece[MeSH Terms])) OR (sperm tail[MeSH Terms])) OR (sperm morphology[Title/Abstract])) OR (sperm form[Title/Abstract])) OR (sperm concentration[Title/Abstract])) OR (semen[Title/Abstract])) OR (semen pH[Title/Abstract])) OR (oligospermia[MeSH Terms])) OR (semen analysis[MeSH Terms])) OR (spermatogenesis[MeSH Terms])) OR (aspermia[MeSH Terms])) OR (asthenozoospermia[MeSH Terms])) OR (azoospermia[MeSH Terms])) OR (sertoli cell-only syndrome[MeSH Terms])) OR (teratozoospermia[MeSH Terms])) OR (semen quality[Title/Abstract])) OR (semen volume[Title/Abstract])) OR (sperm quality[Title/Abstract])) OR (sperm parameter[Title/Abstract])) OR (semen parameter[Title/Abstract])) OR (sperm damage[Title/Abstract])) OR (testicular[Title/Abstract])) OR ((((((((((((((((((reproductive endocrine[Title/Abstract]) OR (hyperandrogenism[MeSH Terms])) OR (gonadotropins[MeSH Terms])) OR (GnRH[Title/Abstract])) OR (goserelin[MeSH Terms])) OR (hypogonadism[MeSH Terms])) OR (hypothalamic-pituitary-gonadal axis[MeSH Terms])) OR (follicle stimulating hormone[MeSH Terms])) OR (dehydroepiandrosterone[MeSH Terms])) OR (prolactin[MeSH Terms])) OR (luteinizing hormone[MeSH Terms])) OR (testosterone[MeSH Terms])) OR (estradiol[MeSH Terms])) OR (sex hormone-binding globulin[MeSH Terms])) OR (Free Androgen Index[Title/Abstract])) OR (FAI[Title/Abstract])) OR (sex hormone[Title/Abstract])) OR (androstenedione[MeSH Terms])) OR (((((((((((((((((((sexual behavior[MeSH Terms]) OR (sexual dysfunctions, psychological[MeSH Terms])) OR (erectile dysfunction[MeSH Terms])) OR (premature ejaculation[MeSH Terms])) OR (erectile disorder[Title/Abstract])) OR (ejaculation dysfunction[Title/Abstract])) OR (early ejaculation[Title/Abstract])) OR (delayed ejaculation[Title/Abstract])) OR (erectile difficulty[Title/Abstract])) OR (sexual and gender disorders[MeSH Terms])) OR (Sexual Function[Title/Abstract])) OR (International Index of

	Erectile Function[Title/Abstract])) OR (IIEF[Title/Abstract])) OR (intravaginal ejaculatory latency time[Title/Abstract])) OR (IELT[Title/Abstract])) OR (ASEX[Title/Abstract])) OR (Arizona Sexual Experience Scale[Title/Abstract])) OR (CSFQ[Title/Abstract])) OR (Changes in Sexual Functioning Questionnaire[Title/Abstract])) OR (((((((((infertility, male[MeSH Terms]) OR (Male Infertility[Title/Abstract])) OR (Sterility, Male[Title/Abstract])) OR (Male Sterility[Title/Abstract])) OR (Subfertility, Male[Title/Abstract])) OR (Male Subfertility[Title/Abstract])) OR (Sub-Fertility, Male[Title/Abstract])) OR (Male Sub-Fertility[Title/Abstract])) OR (Sub Fertility, Male[Title/Abstract]))))) NOT ("animals"[MeSH Terms] NOT "humans"[MeSH Terms])) AND ((((((((((((((((((((((((((((((carbamazepine[Title/Abstract]) OR (anticonvulsants[Title/Abstract])) OR (anticonvulsant*[Title/Abstract])) OR (antiepilep*[Title/Abstract])) OR (epilepsy[Title/Abstract])) OR (epilepsy[Title/Abstract])) OR (epilep*[Title/Abstract])) OR (AED*[Title/Abstract])) OR (phenytoin[Title/Abstract])) OR (gabapentin[Title/Abstract])) OR (valproic acid[Title/Abstract])) OR (valproate[Title/Abstract])) OR (Sodium valproate[Title/Abstract])) OR (oxcarbazepine[Title/Abstract])) OR (lamotrigine[Title/Abstract])) OR (topiramate[Title/Abstract])) OR (levetiracetam[Title/Abstract])) OR (pregabalin[Title/Abstract])) OR (clonazepam[Title/Abstract])) OR (ethosuximide[Title/Abstract])) OR (phenobarbital[Title/Abstract])) OR (felbamate[Title/Abstract])) OR (zonisamide[Title/Abstract])) OR (brivaracetam[Title/Abstract])) OR (clobazam[Title/Abstract])) OR (eslicarbazepine[Title/Abstract])) OR (lacosamide[Title/Abstract])) OR (perampanel[Title/Abstract])) OR (rufinamide[Title/Abstract])) OR (vigabatrin[Title/Abstract]))
**Embase**	('male'/exp/mj OR 'male' OR 'males'/exp/mj OR 'males' OR 'man'/exp/mj OR 'man' OR 'men'/exp/mj OR 'men') AND ('carbamazepine':ab,ti OR 'agent, anticonvulsive':ab,ti OR 'anti convulsant agent':ab,ti OR 'anti epileptic agent':ab,ti OR 'anti epileptic drug':ab,ti OR 'anticonvulsant':ab,ti OR 'anticonvulsant agent':ab,ti OR 'anticonvulsant drug':ab,ti OR 'anticonvulsants':ab,ti OR 'anticonvulsive agent':ab,ti OR 'anticonvulsive drug':ab,ti OR 'anticonvulsivum':ab,ti OR 'antiepileptic agent':ab,ti OR 'antiepileptic barbiturate':ab,ti OR 'antiepileptic drug':ab,ti OR 'antiepileptiform drug':ab,ti OR 'acute epilepsy':ab,ti OR 'attack, epileptic':ab,ti OR 'cerebral seizure, epileptic':ab,ti OR 'chronic epilepsy':ab,ti OR 'comitial disease':ab,ti OR 'convulsion, epileptic':ab,ti OR 'convulsive epilepsy':ab,ti OR 'epilepsia':ab,ti OR 'epilepsy':ab,ti OR 'epilepsy, convulsive':ab,ti OR 'epileptic':ab,ti OR 'epileptic attack':ab,ti OR 'epileptic convulsion':ab,ti OR 'epileptic disorder':ab,ti OR 'epileptic fit':ab,ti OR 'epileptic insult':ab,ti OR 'epileptic seizure':ab,ti OR 'epileptic seizure, cerebral':ab,ti OR 'epileptic syndrome':ab,ti OR 'epileptic syndromes':ab,ti OR 'falling sickness':ab,ti OR 'fit, epileptic':ab,ti OR 'seizure, epileptic':ab,ti OR 'sickness, falling':ab,ti OR 'tardy epilepsy':ab,ti OR aed:ab,ti OR 'phenytoin':ab,ti OR 'gabapentin':ab,ti OR '2 propylpentanoate':ab,ti OR '2 propylpentanoic acid':ab,ti OR '2 propylvalerate sodium':ab,ti OR '2 propylvaleric acid':ab,ti OR '2 propylvaleric acid sodium':ab,ti OR '2, 2 dipropyl acetic acid':ab,ti OR '4 heptanecarboxylic acid':ab,ti OR 'abbott

44090':ab,ti OR 'absenor':ab,ti OR 'absenor depot':ab,ti OR 'alpha propylvalerate':ab,ti OR 'alpha propylvaleric acid':ab,ti OR 'apilepsin':ab,ti OR 'atemperator':ab,ti OR 'convival chrono':ab,ti OR 'convulex':ab,ti OR 'convulex chrono':ab,ti OR 'convulex cr':ab,ti OR 'convulex retard':ab,ti OR 'cs 1':ab,ti OR 'cs1':ab,ti OR 'ct 010':ab,ti OR 'ct010':ab,ti OR 'delepsine':ab,ti OR 'delepsine retard':ab,ti OR 'depacon':ab,ti OR 'depakene':ab,ti OR 'depakene chrono':ab,ti OR 'depakin':ab,ti OR 'depakin chrono':ab,ti OR 'depakine':ab,ti OR 'depakine chrono':ab,ti OR 'depakine chrono retard':ab,ti OR 'depakine chronosphere':ab,ti OR 'depakine crono':ab,ti OR 'depakine druppels':ab,ti OR 'depakine enteric':ab,ti OR 'depakine iv':ab,ti OR 'depalept':ab,ti OR 'depalept chrono':ab,ti OR 'deprakine':ab,ti OR 'deprakine retard':ab,ti OR 'di n propylacetate':ab,ti OR 'di n propylacetate sodium':ab,ti OR 'di n propylacetic acid':ab,ti OR 'diplexil':ab,ti OR 'dipropyl acetic acid':ab,ti OR 'dipropylacetate':ab,ti OR 'dipropylacetate sodium':ab,ti OR 'dipropylacetatic acid':ab,ti OR 'dipropylacetic acid':ab,ti OR 'diprosin':ab,ti OR 'dyzantil':ab,ti OR 'epilam':ab,ti OR 'epilex':ab,ti OR 'epilim':ab,ti OR 'epilim chrono':ab,ti OR 'epilim chrono 500':ab,ti OR 'epilim chronosphere':ab,ti OR 'epilim chronosphere mr':ab,ti OR 'epilim enteric':ab,ti OR 'epilim liquid':ab,ti OR 'episenta':ab,ti OR 'epival cr':ab,ti OR 'ergenyl':ab,ti OR 'ergenyl chrono':ab,ti OR 'ergenyl chronosphere':ab,ti OR 'ergenyl retard':ab,ti OR 'espa valept':ab,ti OR 'espa-valept':ab,ti OR 'everiden':ab,ti OR 'goilim':ab,ti OR 'hexaquin':ab,ti OR 'k 828':ab,ti OR 'k 828ab':ab,ti OR 'k 828sp':ab,ti OR 'k828':ab,ti OR 'k828ab':ab,ti OR 'k828sp':ab,ti OR 'kw 6066 n':ab,ti OR 'labazene':ab,ti OR 'leptilan':ab,ti OR 'leptilanil':ab,ti OR 'micropakine':ab,ti OR 'micropakine lp':ab,ti OR 'mylproin':ab,ti OR 'myproic acid':ab,ti OR 'n dipropylacetic acid':ab,ti OR 'npl 2005':ab,ti OR 'npl2005':ab,ti OR 'orfil':ab,ti OR 'orfiril':ab,ti OR 'orfiril cr':ab,ti OR 'orfiril iv':ab,ti OR 'orfiril long':ab,ti OR 'orfiril retard':ab,ti OR 'orlept':ab,ti OR 'petilin':ab,ti OR 'propymal':ab,ti OR 'propymal enteric':ab,ti OR 'sodium 2 propylpentanoate':ab,ti OR 'sodium 2 propylvalerate':ab,ti OR 'sodium di n propyl acetate':ab,ti OR 'sodium di n propylacetate':ab,ti OR 'sodium dipropyl acetate':ab,ti OR 'sodium dipropylacetate':ab,ti OR 'sodium n dipropylacetate':ab,ti OR 'sodium valproate':ab,ti OR 'sodium valproate plus valproic acid':ab,ti OR 'sodium valproate/valproic acid':ab,ti OR 'stavzor':ab,ti OR 'syonell':ab,ti OR 'val 001 (anticonvulsive agent)':ab,ti OR 'val001 (anticonvulsive agent)':ab,ti OR 'valberg pr':ab,ti OR 'valcote':ab,ti OR 'valepil':ab,ti OR 'valeptol':ab,ti OR 'valerin':ab,ti OR 'valhel pr':ab,ti OR 'valoin':ab,ti OR 'valpakine':ab,ti OR 'valparin':ab,ti OR 'valporal':ab,ti OR 'valprax':ab,ti OR 'valpro':ab,ti OR 'valpro al':ab,ti OR 'valpro beta':ab,ti OR 'valproat':ab,ti OR 'valproat chrono':ab,ti OR 'valproate':ab,ti OR 'valproate retard':ab,ti OR 'valproate sodium':ab,ti OR 'valproate sodium plus valproic acid':ab,ti OR 'valproate sodium/valproic acid':ab,ti OR 'valprodura':ab,ti OR 'valproic acid':ab,ti OR 'valproic acid plus sodium valproate':ab,ti OR 'valproic acid plus valproate sodium':ab,ti OR 'valproic acid/sodium valproate':ab,ti OR 'valproic acid/valproate sodium':ab,ti OR 'valprosid':ab,ti OR 'valprotek':ab,ti OR 'valprotek cr':ab,ti OR 'valsup':ab,ti OR 'vupral':ab,ti OR 'oxcarbazepine':ab,ti OR 'lamotrigine':ab,ti OR 'topiramate':ab,ti OR 'levetiracetam':ab,ti OR 'pregabalin':ab,ti OR 'clonazepam':ab,ti OR 'ethosuximide':ab,ti OR 'phenobarbital':ab,ti OR 'felbamate':ab,ti OR

'zonisamide':ab,ti OR 'brivaracetam':ab,ti OR 'clobazam':ab,ti OR 'eslicarbazepine':ab,ti OR 'lacosamide':ab,ti OR 'perampanel':ab,ti OR 'rufinamide':ab,ti OR 'vigabatrin':ab,ti) AND ('semen count'/exp/mj OR 'semen count' OR 'semen number'/exp/mj OR 'semen number' OR 'seminal count'/exp/mj OR 'seminal count' OR 'sperm count'/exp/mj OR 'sperm count' OR 'sperm number'/exp/mj OR 'sperm number' OR 'spermatid count'/exp/mj OR 'spermatid count' OR 'spermatid number'/exp/mj OR 'spermatid number' OR 'spermatozoa count'/exp/mj OR 'spermatozoa count' OR 'spermatozoa number'/exp/mj OR 'spermatozoa number' OR 'spermatozoal count'/exp/mj OR 'spermatozoal count' OR 'spermatozoal number'/exp/mj OR 'spermatozoal number' OR 'spermatozoan count'/exp/mj OR 'spermatozoan count' OR 'spermatozoan number'/exp/mj OR 'spermatozoan number' OR 'spermatozoid count'/exp/mj OR 'spermatozoid count' OR 'spermatozoid number'/exp/mj OR 'spermatozoid number' OR 'spermatozoon count'/exp/mj OR 'spermatozoon count' OR 'spermatozoon number'/exp/mj OR 'spermatozoon number' OR 'spermium count'/exp/mj OR 'spermium count' OR 'spermatozoon motility'/exp/mj OR 'spermatozoon motility' OR 'spermatic cell'/exp/mj OR 'spermatic cell' OR 'spermatozoa'/exp/mj OR 'spermatozoa' OR 'spermatozoid'/exp/mj OR 'spermatozoid' OR 'spermatozoon'/exp/mj OR 'spermatozoon' OR 'sperm morphology'/exp/mj OR 'sperm morphology' OR 'spermatozoon head'/exp/mj OR 'spermatozoon head' OR 'spermatozoon midpiece'/exp/mj OR 'spermatozoon midpiece' OR 'spermatozoon tail'/exp/mj OR 'spermatozoon tail' OR 'sperm form' OR 'hypospermia'/exp/mj OR 'hypospermia' OR 'oligoasthenospermia'/exp/mj OR 'oligoasthenospermia' OR 'oligoasthenospermy'/exp/mj OR 'oligoasthenospermy' OR 'oligospermia'/exp/mj OR 'oligospermia' OR 'oligospermy'/exp/mj OR 'oligospermy' OR 'oligozoospermia'/exp/mj OR 'oligozoospermia' OR 'semen density'/exp/mj OR 'semen density' OR 'seminal density'/exp/mj OR 'seminal density' OR 'sperm concentration'/exp/mj OR 'sperm concentration' OR 'sperm density'/exp/mj OR 'sperm density' OR 'spermatozoal density'/exp/mj OR 'spermatozoal density' OR 'spermatozoon density'/exp/mj OR 'spermatozoon density' OR 'ejaculate'/exp/mj OR 'ejaculate' OR 'semen'/exp/mj OR 'semen' OR 'sperm'/exp/mj OR 'sperm' OR 'sperm utilization'/exp/mj OR 'sperm utilization' OR 'sperma'/exp/mj OR 'sperma' OR 'quality, sperm'/exp/mj OR 'quality, sperm' OR 'semen quality'/exp/mj OR 'semen quality' OR 'seminal quality'/exp/mj OR 'seminal quality' OR 'sperm quality'/exp/mj OR 'sperm quality' OR 'spermatic quality'/exp/mj OR 'spermatic quality' OR 'spermatid quality'/exp/mj OR 'spermatid quality' OR 'spermatozoa quality'/exp/mj OR 'spermatozoa quality' OR 'spermatozoal quality'/exp/mj OR 'spermatozoal quality' OR 'spermatozoan quality'/exp/mj OR 'spermatozoan quality' OR 'spermatozoid quality'/exp/mj OR 'spermatozoid quality' OR 'spermatozoon quality'/exp/mj OR 'spermatozoon quality' OR 'semen volume'/exp/mj OR 'semen volume' OR 'sperm production'/exp/mj OR 'sperm production' OR 'spermatidogenesis'/exp/mj OR 'spermatidogenesis' OR 'spermatocytogenesis'/exp/mj OR 'spermatocytogenesis' OR 'spermatogenesis'/exp/mj OR 'spermatogenesis' OR 'spermatogenic cycle'/exp/mj OR 'spermatogenic cycle' OR 'spermatogenic process'/exp/mj OR 'spermatogenic process' OR 'spermiogenesis'/exp/mj OR 'spermiogenesis' OR 'aspermatism'/exp/mj OR 'aspermatism' OR

'aspermia'/exp/mj OR 'aspermia' OR 'asthenazoospermia'/exp/mj OR 'asthenazoospermia' OR 'asthenospermia'/exp/mj OR 'asthenospermia' OR 'asthenozoospermia'/exp/mj OR 'asthenozoospermia' OR 'azoospermia'/exp/mj OR 'azoospermia' OR 'sco syndrome'/exp/mj OR 'sco syndrome' OR 'sertoli cell only syndrome'/exp/mj OR 'sertoli cell only syndrome' OR 'sertoli cell-only syndrome'/exp/mj OR 'sertoli cell-only syndrome' OR 'spermatozoon abnormality'/exp/mj OR 'spermatozoon abnormality' OR 'spermatozoon anomaly'/exp/mj OR 'spermatozoon anomaly' OR 'teratozoospermia'/exp/mj OR 'teratozoospermia' OR 'semen parameters'/exp/mj OR 'semen parameters' OR 'seminal parameters'/exp/mj OR 'seminal parameters' OR 'sperm parameters'/exp/mj OR 'sperm parameters' OR 'spermatic parameters'/exp/mj OR 'spermatic parameters' OR 'spermatid parameters'/exp/mj OR 'spermatid parameters' OR 'spermatozoa parameters'/exp/mj OR 'spermatozoa parameters' OR 'spermatozoal parameters'/exp/mj OR 'spermatozoal parameters' OR 'spermatozoan parameters'/exp/mj OR 'spermatozoan parameters' OR 'spermatozoid parameters'/exp/mj OR 'spermatozoid parameters' OR 'spermatozoon parameters'/exp/mj OR 'spermatozoon parameters' OR 'sperm damage' OR 'haematocele'/exp/mj OR 'haematocele' OR 'hematocele'/exp/mj OR 'hematocele' OR 'orchidopathy'/exp/mj OR 'orchidopathy' OR 'orchiopathy'/exp/mj OR 'orchiopathy' OR 'testicular disease'/exp/mj OR 'testicular disease' OR 'testicular diseases'/exp/mj OR 'testicular diseases' OR 'testis disease'/exp/mj OR 'testis disease' OR 'testis disorder'/exp/mj OR 'testis disorder' OR 'reproductive endocrine' OR 'hyperandro-genism'/exp/mj OR 'hyperandro-genism' OR 'hyperandrogenemia'/exp/mj OR 'hyperandrogenemia' OR 'hyperandrogenism'/exp/mj OR 'hyperandrogenism' OR 'ambinon'/exp/mj OR 'ambinon' OR 'anthrogon'/exp/mj OR 'anthrogon' OR 'carp pituitary gonadotropin'/exp/mj OR 'carp pituitary gonadotropin' OR 'folistiman'/exp/mj OR 'folistiman' OR 'gonad stimulating hormone'/exp/mj OR 'gonad stimulating hormone' OR 'gonad stimulatory hormone'/exp/mj OR 'gonad stimulatory hormone' OR 'gonadotrophic hormone'/exp/mj OR 'gonadotrophic hormone' OR 'gonadotrophin'/exp/mj OR 'gonadotrophin' OR 'gonadotropic hormone'/exp/mj OR 'gonadotropic hormone' OR 'gonadotropin'/exp/mj OR 'gonadotropin' OR 'gonadotropin, pituitary'/exp/mj OR 'gonadotropin, pituitary' OR 'gonadotropine'/exp/mj OR 'gonadotropine' OR 'gonadotropins'/exp/mj OR 'gonadotropins' OR 'gonadotropins, pituitary'/exp/mj OR 'gonadotropins, pituitary' OR 'gonan'/exp/mj OR 'gonan' OR 'human gonadotropin'/exp/mj OR 'human gonadotropin' OR 'hypophyseal gonadotropin'/exp/mj OR 'hypophyseal gonadotropin' OR 'hypophysis gonadotropin'/exp/mj OR 'hypophysis gonadotropin' OR 'pituitary gonadotrophin'/exp/mj OR 'pituitary gonadotrophin' OR 'pituitary gonadotropin'/exp/mj OR 'pituitary gonadotropin' OR 'pregonadotropin'/exp/mj OR 'pregonadotropin' OR '6 leucine 10 deglycine gonadotropin releasing factor ethylamide'/exp/mj OR '6 leucine 10 deglycine gonadotropin releasing factor ethylamide' OR 'lhrh'/exp/mj OR 'lhrh' OR '[6 leucine 10 deglycine] gonadotropin releasing factor ethylamide'/exp/mj OR '[6 leucine 10 deglycine] gonadotropin releasing factor ethylamide' OR 'ay 24, 031'/exp/mj OR 'ay 24, 031' OR 'ay 24031'/exp/mj OR 'ay 24031' OR 'ay24, 031'/exp/mj OR 'ay24, 031' OR 'ay24031'/exp/mj OR 'ay24031' OR

'cryptocur'/exp/mj OR 'cryptocur' OR 'factrel'/exp/mj OR 'factrel' OR 'fertagyl'/exp/mj OR 'fertagyl' OR 'fertiral'/exp/mj OR 'fertiral' OR 'gn rh'/exp/mj OR 'gn rh' OR 'gnrh'/exp/mj OR 'gnrh' OR 'gnrh serono'/exp/mj OR 'gnrh serono' OR 'gonadoliberin'/exp/mj OR 'gonadoliberin' OR 'gonadorelin'/exp/mj OR 'gonadorelin' OR 'gonadorelin hydrochloride'/exp/mj OR 'gonadorelin hydrochloride' OR 'gonadotrophin releasing factor'/exp/mj OR 'gonadotrophin releasing factor' OR 'gonadotrophin releasing hormone'/exp/mj OR 'gonadotrophin releasing hormone' OR 'gonadotropin release factor'/exp/mj OR 'gonadotropin release factor' OR 'gonadotropin releasing factor'/exp/mj OR 'gonadotropin releasing factor' OR 'gonadotropin releasing hormone'/exp/mj OR 'gonadotropin releasing hormone' OR 'gonadotropin-releasing hormone'/exp/mj OR 'gonadotropin-releasing hormone' OR 'hoe 471'/exp/mj OR 'hoe 471' OR 'hoe471'/exp/mj OR 'hoe471' OR 'hoechst 471'/exp/mj OR 'hoechst 471' OR 'ici 88262'/exp/mj OR 'ici 88262' OR 'ici88262'/exp/mj OR 'ici88262' OR 'kryptocur'/exp/mj OR 'kryptocur' OR 'lh releasing hormone'/exp/mj OR 'lh releasing hormone' OR 'lhrf'/exp/mj OR 'lhrf' OR 'lrh'/exp/mj OR 'lrh' OR 'lrh luteinising hormone releasing factor'/exp/mj OR 'lrh luteinising hormone releasing factor' OR 'lrh luteinizing hormone releasing factor'/exp/mj OR 'lrh luteinizing hormone releasing factor' OR 'luforan'/exp/mj OR 'luforan' OR 'luliberin'/exp/mj OR 'luliberin' OR 'luliberine'/exp/mj OR 'luliberine' OR 'lutal'/exp/mj OR 'lutal' OR 'lutamin'/exp/mj OR 'lutamin' OR 'luteinising hormone release factor'/exp/mj OR 'luteinising hormone release factor' OR 'luteinising hormone releasing factor'/exp/mj OR 'luteinising hormone releasing factor' OR 'luteinising hormone releasing hormone'/exp/mj OR 'luteinising hormone releasing hormone' OR 'luteinizing hormone release factor'/exp/mj OR 'luteinizing hormone release factor' OR 'luteinizing hormone releasing factor'/exp/mj OR 'luteinizing hormone releasing factor' OR 'luteinizing hormone releasing hormone'/exp/mj OR 'luteinizing hormone releasing hormone' OR 'pulstim'/exp/mj OR 'pulstim' OR 'relisorm'/exp/mj OR 'relisorm' OR '1 (5 oxoprolylhistidyltryptophylseryltyrosyl o tertbutyl dextro serylleucylarginylprolyl) semicarbazide'/exp/mj OR '1 (5 oxoprolylhistidyltryptophylseryltyrosyl o tertbutyl dextro serylleucylarginylprolyl) semicarbazide' OR 'buserelin carbazamide'/exp/mj OR 'buserelin carbazamide' OR 'gonadorelin [1-9] [6 (o tert butyl dextro serine)] carbazamide'/exp/mj OR 'gonadorelin [1-9] [6 (o tert butyl dextro serine)] carbazamide' OR 'gonadorelin [1-9] carbazamide [6 (o tert butyl dextro serine)]'/exp/mj OR 'gonadorelin [1-9] carbazamide [6 (o tert butyl dextro serine)]' OR 'gonadorelin [6 dextro o tert butylserine 10 glycine azide]'/exp/mj OR 'gonadorelin [6 dextro o tert butylserine 10 glycine azide]' OR 'goserelin'/exp/mj OR 'goserelin' OR 'goserelin acetate'/exp/mj OR 'goserelin acetate' OR 'ici 118 630'/exp/mj OR 'ici 118 630' OR 'ici 118630'/exp/mj OR 'ici 118630' OR 'ici 118630 depot'/exp/mj OR 'ici 118630 depot' OR 'ici118630'/exp/mj OR 'ici118630' OR 'lhrh [6 dextro o tert butylserine 10 glycine azide]'/exp/mj OR 'lhrh [6 dextro o tert butylserine 10 glycine azide]' OR 'ly 01005'/exp/mj OR 'ly 01005' OR 'ly01005'/exp/mj OR 'ly01005' OR 'n [21 [(1h indol 3 yl) methyl] 1, 1 diamino 12 (tert butoxymethyl) 6 (2 (2 carbamoylhydrazinecarbonyl) cyclopentanecarbonyl) 15 (4 hydroxybenzyl) 18 (hydroxymethyl) 25 (1h imidazol 5 yl) 9 isobutyl 8,

11, 14, 17, 20, 23 hexaoxo 2, 7, 10, 13, 16, 19, 22 heptaazapentacos 1 en 24 yl] 5 oxopyrrolidine 2 carboxamide'/exp/mj OR 'n [21 [(1h indol 3 yl) methyl] 1, 1 diamino 12 (tert butoxymethyl) 6 (2 (2 carbamoylhydrazinecarbonyl) cyclopentanecarbonyl) 15 (4 hydroxybenzyl) 18 (hydroxymethyl) 25 (1h imidazol 5 yl) 9 isobutyl 8, 11, 14, 17, 20, 23 hexaoxo 2, 7, 10, 13, 16, 19, 22 heptaazapentacos 1 en 24 yl] 5 oxopyrrolidine 2 carboxamide' OR 'novimp'/exp/mj OR 'novimp' OR 'prozoladex'/exp/mj OR 'prozoladex' OR 'pyroglutamylhistidyltryptophylseryltyrosyl (o tert butyl dextro seryl) leucylarginylprolylcarbazamide'/exp/mj OR 'pyroglutamylhistidyltryptophylseryltyrosyl (o tert butyl dextro seryl) leucylarginylprolylcarbazamide' OR 'reseligo'/exp/mj OR 'reseligo' OR 'zd 9393'/exp/mj OR 'zd 9393' OR 'zd9393'/exp/mj OR 'zd9393' OR 'zoladex'/exp/mj OR 'zoladex' OR 'zoladex depot'/exp/mj OR 'zoladex depot' OR 'zoladex inj.'/exp/mj OR 'zoladex inj.' OR 'zoladex la'/exp/mj OR 'zoladex la' OR 'zoreline'/exp/mj OR 'zoreline' OR 'hypogonadism, hypogonadotropic'/exp/mj OR 'hypogonadism, hypogonadotropic' OR 'hypogonadotrophic hypogonadism'/exp/mj OR 'hypogonadotrophic hypogonadism' OR 'hypogonadotrophism'/exp/mj OR 'hypogonadotrophism' OR 'hypogonadotropic hypogonadism'/exp/mj OR 'hypogonadotropic hypogonadism' OR 'hypogonadotropism'/exp/mj OR 'hypogonadotropism' OR 'male hypogonadotrophic hypogonadism'/exp/mj OR 'male hypogonadotrophic hypogonadism' OR 'hypothalamus hypophysis gonad system'/exp/mj OR 'hypothalamus hypophysis gonad system' OR 'fsh'/exp/mj OR 'fsh' OR 'fertiline'/exp/mj OR 'fertiline' OR 'fertinom p'/exp/mj OR 'fertinom p' OR 'follicle stimulating hormone'/exp/mj OR 'follicle stimulating hormone' OR 'follicle stimulating hormone, human'/exp/mj OR 'follicle stimulating hormone, human' OR 'follicotropin'/exp/mj OR 'follicotropin' OR 'folliculostimulating hormone'/exp/mj OR 'folliculostimulating hormone' OR 'follitrophin'/exp/mj OR 'follitrophin' OR 'follitropin'/exp/mj OR 'follitropin' OR 'follitropine'/exp/mj OR 'follitropine' OR 'folltropin'/exp/mj OR 'folltropin' OR 'fsh nosticon'/exp/mj OR 'fsh nosticon' OR 'human follicle stimulating hormone'/exp/mj OR 'human follicle stimulating hormone' OR 'human follitropin'/exp/mj OR 'human follitropin' OR 'human fsh'/exp/mj OR 'human fsh' OR 'ovagen'/exp/mj OR 'ovagen' OR 'ovine follicle stimulating hormone'/exp/mj OR 'ovine follicle stimulating hormone' OR 'ovine fsh'/exp/mj OR 'ovine fsh' OR 'rat follitropin'/exp/mj OR 'rat follitropin' OR 'rat fsh'/exp/mj OR 'rat fsh' OR 'super ov'/exp/mj OR 'super ov' OR '17 chetovis'/exp/mj OR '17 chetovis' OR '17 hormoforin'/exp/mj OR '17 hormoforin' OR '17 oxo 5 androsten 3beta ol'/exp/mj OR '17 oxo 5 androsten 3beta ol' OR '3 beta hydroxy 5 androsten 17 one'/exp/mj OR '3 beta hydroxy 5 androsten 17 one' OR '3 beta hydroxyandrost 5 en 17 one'/exp/mj OR '3 beta hydroxyandrost 5 en 17 one' OR '3 hydroxyetioallocholan 17 one'/exp/mj OR '3 hydroxyetioallocholan 17 one' OR '3beta hydroxy 17 androstanone'/exp/mj OR '3beta hydroxy 17 androstanone' OR '3beta hydroxy 5 androsten 17 one'/exp/mj OR '3beta hydroxy 5 androsten 17 one' OR '3beta hydroxyandrost 5 en 17 one'/exp/mj OR '3beta hydroxyandrost 5 en 17 one' OR '3beta hydroxyandrost 5 ene 17 one'/exp/mj OR '3beta hydroxyandrost 5 ene 17 one' OR '3beta hydroxyetioallocholan 17 one'/exp/mj OR '3beta hydroxyetioallocholan 17 one' OR '5 alpha androsten 3 beta ol 17 one'/exp/mj

OR '5 alpha androsten 3 beta ol 17 one' OR '5 androsten 3 beta 17 one'/exp/mj OR '5 androsten 3 beta 17 one' OR '5 androsten 3 beta ol 17 one'/exp/mj OR '5 androsten 3 beta ol 17 one' OR '5 androsten 3 ol 17 one'/exp/mj OR '5 androsten 3 ol 17 one' OR '5 androsten 3beta ol 17 one'/exp/mj OR '5 androsten 3beta ol 17 one' OR '5 androstene 3 beta ol 17 one'/exp/mj OR '5 androstene 3 beta ol 17 one' OR '5 androstene 3beta ol 17 one'/exp/mj OR '5 androstene 3beta ol 17 one' OR '5alpha androsten 3 beta ol 17 one'/exp/mj OR '5alpha androsten 3 beta ol 17 one' OR '5alpha androsten 3beta ol 17 one'/exp/mj OR '5alpha androsten 3beta ol 17 one' OR '7alpha (3h) dehydroepiandrosterone'/exp/mj OR '7alpha (3h) dehydroepiandrosterone' OR '7alpha dehydro epiandrosterone'/exp/mj OR '7alpha dehydro epiandrosterone' OR '7alpha dehydroepiandrosterone'/exp/mj OR '7alpha dehydroepiandrosterone' OR 'dhea'/exp/mj OR 'dhea' OR 'anastar'/exp/mj OR 'anastar' OR 'androst 5 en 3 beta ol 17 one'/exp/mj OR 'androst 5 en 3 beta ol 17 one' OR 'androst 5 en 3beta ol 17 one'/exp/mj OR 'androst 5 en 3beta ol 17 one' OR 'androst 5 ene 3beta ol 17 one'/exp/mj OR 'androst 5 ene 3beta ol 17 one' OR 'androstane 17 one'/exp/mj OR 'androstane 17 one' OR 'androstene 5 3b ol 17 one'/exp/mj OR 'androstene 5 3b ol 17 one' OR 'aslera'/exp/mj OR 'aslera' OR 'astenile'/exp/mj OR 'astenile' OR 'chetovis'/exp/mj OR 'chetovis' OR 'dehydro epiandrosterone'/exp/mj OR 'dehydro epiandrosterone' OR 'dehydroepiandrosteron'/exp/mj OR 'dehydroepiandrosteron' OR 'dehydroepiandrosterone'/exp/mj OR 'dehydroepiandrosterone' OR 'dehydroisoandrosterone'/exp/mj OR 'dehydroisoandrosterone' OR 'dehyroepiandrosterone'/exp/mj OR 'dehyroepiandrosterone' OR 'delta 5 androsten 3 beta ol 17 one'/exp/mj OR 'delta 5 androsten 3 beta ol 17 one' OR 'delta 5 androsten 3beta ol 17 one'/exp/mj OR 'delta 5 androsten 3beta ol 17 one' OR 'delta5 androsten 3 beta 17 one'/exp/mj OR 'delta5 androsten 3 beta 17 one' OR 'delta5 androsten 3beta 17 one'/exp/mj OR 'delta5 androsten 3beta 17 one' OR 'delta5 androsten 3beta ol 17 one'/exp/mj OR 'delta5 androsten 3beta ol 17 one' OR 'diandron'/exp/mj OR 'diandron' OR 'diandrone'/exp/mj OR 'diandrone' OR 'dihydroepiandrosterone'/exp/mj OR 'dihydroepiandrosterone' OR 'dihydroisoandrosterone'/exp/mj OR 'dihydroisoandrosterone' OR 'el 10'/exp/mj OR 'el 10' OR 'el10'/exp/mj OR 'el10' OR 'em 760'/exp/mj OR 'em 760' OR 'em760'/exp/mj OR 'em760' OR 'fidelin'/exp/mj OR 'fidelin' OR 'gl 701'/exp/mj OR 'gl 701' OR 'gl701'/exp/mj OR 'gl701' OR 'hormoformin'/exp/mj OR 'hormoformin' OR 'intrarosa'/exp/mj OR 'intrarosa' OR 'pb 005'/exp/mj OR 'pb 005' OR 'pb005'/exp/mj OR 'pb005' OR 'prasterone'/exp/mj OR 'prasterone' OR 'prestara'/exp/mj OR 'prestara' OR 'psicosterone'/exp/mj OR 'psicosterone' OR 'trans dehydroandrosterone'/exp/mj OR 'trans dehydroandrosterone' OR 'prolactin'/exp/mj OR 'prolactin' OR 'luteinizing hormone'/exp/mj OR 'luteinizing hormone' OR 'testosterone'/exp/mj OR 'testosterone' OR 'estradiol'/exp/mj OR 'estradiol' OR 'sex hormone binding globulin'/exp/mj OR 'sex hormone binding globulin' OR 'free androgen index'/exp/mj OR 'free androgen index' OR 'androstenedione'/exp/mj OR 'androstenedione' OR 'gonadal hormone'/exp/mj OR 'gonadal hormone' OR 'gonadal hormones'/exp/mj OR 'gonadal hormones' OR 'gonadal steroid hormone'/exp/mj OR 'gonadal steroid hormone' OR 'gonadal steroid hormones'/exp/mj OR 'gonadal steroid hormones' OR 'hormone, sex'/exp/mj

OR 'hormone, sex' OR 'sex hormone'/exp/mj OR 'sex hormone' OR 'sex hormones'/exp/mj OR 'sex hormones' OR 'sex steroid'/exp/mj OR 'sex steroid' OR 'sex steroid hormone'/exp/mj OR 'sex steroid hormone' OR 'sexual hormone'/exp/mj OR 'sexual hormone' OR 'steroid, sex'/exp/mj OR 'steroid, sex' OR 'animal sexual behavior'/exp/mj OR 'animal sexual behavior' OR 'behavior, sexual'/exp/mj OR 'behavior, sexual' OR 'behaviour, sexual'/exp/mj OR 'behaviour, sexual' OR 'sex behavior'/exp/mj OR 'sex behavior' OR 'sex behavior, animal'/exp/mj OR 'sex behavior, animal' OR 'sex behaviour'/exp/mj OR 'sex behaviour' OR 'sex behaviour, animal'/exp/mj OR 'sex behaviour, animal' OR 'sex life'/exp/mj OR 'sex life' OR 'sexual action'/exp/mj OR 'sexual action' OR 'sexual activity'/exp/mj OR 'sexual activity' OR 'sexual behavior'/exp/mj OR 'sexual behavior' OR 'sexual behavior, animal'/exp/mj OR 'sexual behavior, animal' OR 'sexual behaviour'/exp/mj OR 'sexual behaviour' OR 'sexual behaviour, animal'/exp/mj OR 'sexual behaviour, animal' OR 'sexual life'/exp/mj OR 'sexual life' OR 'sexual receptivity'/exp/mj OR 'sexual receptivity' OR 'sexual response'/exp/mj OR 'sexual response' OR 'ego-dystonic homosexuality'/exp/mj OR 'ego-dystonic homosexuality' OR 'ego-dystonic sexual orientation'/exp/mj OR 'ego-dystonic sexual orientation' OR 'egodystonic homosexuality'/exp/mj OR 'egodystonic homosexuality' OR 'egodystonic sexual orientation'/exp/mj OR 'egodystonic sexual orientation' OR 'homosexuality, ego-dystonic'/exp/mj OR 'homosexuality, ego-dystonic' OR 'psycho-sexual disorder'/exp/mj OR 'psycho-sexual disorder' OR 'psycho-sexual dysfunction'/exp/mj OR 'psycho-sexual dysfunction' OR 'psychogenic sexual disorder'/exp/mj OR 'psychogenic sexual disorder' OR 'psychogenic sexual dysfunction'/exp/mj OR 'psychogenic sexual dysfunction' OR 'psychological sexual disease'/exp/mj OR 'psychological sexual disease' OR 'psychological sexual disorder'/exp/mj OR 'psychological sexual disorder' OR 'psychological sexual dysfunction'/exp/mj OR 'psychological sexual dysfunction' OR 'psychological sexual dysfunctions'/exp/mj OR 'psychological sexual dysfunctions' OR 'psychosexual disorder'/exp/mj OR 'psychosexual disorder' OR 'psychosexual disorders'/exp/mj OR 'psychosexual disorders' OR 'psychosexual disturbance'/exp/mj OR 'psychosexual disturbance' OR 'psychosexual dysfunction'/exp/mj OR 'psychosexual dysfunction' OR 'psychosexual dysfunctions'/exp/mj OR 'psychosexual dysfunctions' OR 'sexual dysfunctions, psychological'/exp/mj OR 'sexual dysfunctions, psychological' OR 'dysfunction, erectile'/exp/mj OR 'dysfunction, erectile' OR 'erectile disability'/exp/mj OR 'erectile disability' OR 'erectile dysfunction'/exp/mj OR 'erectile dysfunction' OR 'erectile failure'/exp/mj OR 'erectile failure' OR 'erectile impotence'/exp/mj OR 'erectile impotence' OR 'erectile disorder' OR 'ejaculatio praecox'/exp/mj OR 'ejaculatio praecox' OR 'ejaculatio precox'/exp/mj OR 'ejaculatio precox' OR 'premature ejaculation'/exp/mj OR 'premature ejaculation' OR 'abnormal ejaculation'/exp/mj OR 'abnormal ejaculation' OR 'ejaculation disorder'/exp/mj OR 'ejaculation disorder' OR 'ejaculation dysfunction'/exp/mj OR 'ejaculation dysfunction' OR 'ejaculatory disease'/exp/mj OR 'ejaculatory disease' OR 'ejaculatory disorder'/exp/mj OR 'ejaculatory disorder' OR 'ejaculatory dysfunction'/exp/mj OR 'ejaculatory dysfunction' OR 'early ejaculation' OR 'delayed ejaculation'/exp/mj OR 'delayed ejaculation' OR 'erectile difficulty' OR 'coital disorder'/exp/mj OR 'coital disorder' OR 'coital

	dysfunction'/exp/mj OR 'coital dysfunction' OR 'dysfunction, sexual'/exp/mj OR 'dysfunction, sexual' OR 'physiological sexual dysfunction'/exp/mj OR 'physiological sexual dysfunction' OR 'sex abnormality'/exp/mj OR 'sex abnormality' OR 'sex disorders'/exp/mj OR 'sex disorders' OR 'sex dysfunction'/exp/mj OR 'sex dysfunction' OR 'sex insufficiency'/exp/mj OR 'sex insufficiency' OR 'sex problem'/exp/mj OR 'sex problem' OR 'sexual and gender disorders'/exp/mj OR 'sexual and gender disorders' OR 'sexual asthenia'/exp/mj OR 'sexual asthenia' OR 'sexual disability'/exp/mj OR 'sexual disability' OR 'sexual disorder'/exp/mj OR 'sexual disorder' OR 'sexual disturbance'/exp/mj OR 'sexual disturbance' OR 'sexual dysfunction'/exp/mj OR 'sexual dysfunction' OR 'sexual dysfunction, physiological'/exp/mj OR 'sexual dysfunction, physiological' OR 'sexual malfunction'/exp/mj OR 'sexual malfunction' OR 'sexual problem'/exp/mj OR 'sexual problem' OR 'sexual function'/exp/mj OR 'sexual function' OR 'iief'/exp/mj OR 'iief' OR 'iief (international index of erectile function)'/exp/mj OR 'iief (international index of erectile function)' OR 'international index of erectile function'/exp/mj OR 'international index of erectile function' OR 'intravaginal ejaculatory latency time'/exp/mj OR 'intravaginal ejaculatory latency time' OR ielt OR 'international index of erectile function 5'/exp/mj OR 'international index of erectile function 5' OR 'international index of erectile function questionnaire'/exp/mj OR 'international index of erectile function questionnaire' OR 'arizona sexual experience scale'/exp/mj OR 'arizona sexual experience scale' OR asex OR 'changes in sexual functioning questionnaire'/exp/mj OR 'changes in sexual functioning questionnaire' OR csfq OR 'infertility, male'/exp/mj OR 'infertility, male' OR 'male infertility'/exp/mj OR 'male infertility' OR 'male sterility'/exp/mj OR 'male sterility' OR 'sterility, male'/exp/mj OR 'sterility, male' OR 'subfertility, male' OR 'male subfertility'/exp/mj OR 'male subfertility' OR 'sub-fertility, male' OR 'male sub-fertility' OR 'sub fertility, male') AND [humans]/lim
**Cochrane CENTRAL**	#1 (("carbamazepine" OR "agent anticonvulsive" OR "anti convulsant agent" OR "anti epileptic agent" OR "anti epileptic drug" OR "anticonvulsant" OR "anticonvulsant agent" OR "anticonvulsant drug" OR "anticonvulsants" OR "anticonvulsive agent" OR "anticonvulsive drug" OR "anticonvulsivum" OR "antiepileptic agent" OR "antiepileptic barbiturate" OR "antiepileptic drug" OR "antiepileptiform drug" OR "acute epilepsy" OR "attack epileptic" OR "cerebral seizure epileptic" OR "chronic epilepsy" OR "comitial disease" OR "convulsion epileptic" OR "convulsive epilepsy" OR "epilepsia" OR "epilepsy" OR "epilepsy convulsive" OR "epileptic" OR "epileptic attack" OR "epileptic convulsion" OR "epileptic disorder" OR "epileptic fit" OR "epileptic insult" OR "epileptic seizure" OR "epileptic seizure cerebral" OR "epileptic syndrome" OR "epileptic syndromes" OR "falling sickness" OR "fit epileptic" OR "seizure epileptic" OR "sickness falling" OR "tardy epilepsy" OR "aed" OR "phenytoin" OR "gabapentin" OR "2 propylpentanoate" OR "2 propylpentanoic acid" OR "2 propylvalerate sodium" OR "2 propylvaleric acid" OR "2 propylvaleric acid sodium" OR "2 2 dipropyl acetic acid" OR "4 heptanecarboxylic acid" OR "abbott 44090" OR "absenor" OR "absenor depot" OR "alpha propylvalerate" OR "alpha propylvaleric acid" OR "apilepsin" OR "atemperator" OR "convival chrono" OR "convulex" OR "convulex chrono" OR "convulex cr" OR "convulex

retard" OR "cs 1" OR "cs1" OR "ct 010" OR "ct010" OR "delepsine" OR "delepsine retard" OR "depacon" OR "depakene" OR "depakene chrono" OR "depakin" OR "depakin chrono" OR "depakine" OR "depakine chrono" OR "depakine chrono retard" OR "depakine chronosphere" OR "depakine crono" OR "depakine druppels" OR "depakine enteric" OR "depakine iv" OR "depalept" OR "depalept chrono" OR "deprakine" OR "deprakine retard" OR "di n propylacetate" OR "di n propylacetate sodium" OR "di n propylacetic acid" OR "diplexil" OR "dipropyl acetic acid" OR "dipropylacetate" OR "dipropylacetate sodium" OR "dipropylacetatic acid" OR "dipropylacetic acid" OR "diprosin" OR "dyzantil" OR "epilam" OR "epilex" OR "epilim" OR "epilim chrono" OR "epilim chrono 500" OR "epilim chronosphere" OR "epilim chronosphere mr" OR "epilim enteric" OR "epilim liquid" OR "episenta" OR "epival cr" OR "ergenyl" OR "ergenyl chrono" OR "ergenyl chronosphere" OR "ergenyl retard" OR "espa valept" OR "espa-valept" OR "everiden" OR "goilim" OR "hexaquin" OR "k 828" OR "k 828ab" OR "k 828sp" OR "k828" OR "k828ab" OR "k828sp" OR "kw 6066 n" OR "labazene" OR "leptilan" OR "leptilanil" OR "micropakine" OR "micropakine lp" OR "mylproin" OR "myproic acid" OR "n dipropylacetic acid" OR "npl 2005" OR "npl2005" OR "orfil" OR "orfiril" OR "orfiril cr" OR "orfiril iv" OR "orfiril long" OR "orfiril retard" OR "orlept" OR "petilin" OR "propymal" OR "propymal enteric" OR "sodium 2 propylpentanoate" OR "sodium 2 propylvalerate" OR "sodium di n propyl acetate" OR "sodium di n propylacetate" OR "sodium dipropyl acetate" OR "sodium dipropylacetate" OR "sodium n dipropylacetate" OR "sodium valproate" OR "sodium valproate plus valproic acid" OR "sodium valproate/valproic acid" OR "stavzor" OR "syonell" OR "val 001 anticonvulsive agent" OR "val001 anticonvulsive agent" OR "valberg pr" OR "valcote" OR "valepil" OR "valeptol" OR "valerin" OR "valhel pr" OR "valoin" OR "valpakine" OR "valparin" OR "valporal" OR "valprax" OR "valpro" OR "valpro al" OR "valpro beta" OR "valproat" OR "valproat chrono" OR "valproate" OR "valproate retard" OR "valproate sodium" OR "valproate sodium plus valproic acid" OR "valproate sodium/valproic acid" OR "valprodura" OR "valproic acid" OR "valproic acid plus sodium valproate" OR "valproic acid plus valproate sodium" OR "valproic acid/sodium valproate" OR "valproic acid/valproate sodium" OR "valprosid" OR "valprotek" OR "valprotek cr" OR "valsup" OR "vupral" OR "oxcarbazepine" OR "lamotrigine" OR "topiramate" OR "levetiracetam" OR "pregabalin" OR "clonazepam" OR "ethosuximide" OR "phenobarbital" OR "felbamate" OR "zonisamide" OR "brivaracetam" OR "clobazam" OR "eslicarbazepine" OR "lacosamide" OR "perampanel" OR "rufinamide" OR "vigabatrin")):ti,ab,kw

#2 MeSH descriptor: [Anticonvulsants] explode all trees and with qualifier(s): [adverse effects - AE, therapeutic use - TU]

#3 MeSH descriptor: [Infertility, Male] explode all trees

#4 "sperm count" OR "sperm motility" OR "spermatozoa" OR "sperm head" OR "sperm midpiece" OR "sperm tail" OR "sperm morphology" OR "sperm form" OR "sperm concentration" OR "semen" OR "semen pH" OR

"oligospermia" OR "semen analysis" OR "spermatogenesis" OR "aspermia" OR "asthenozoospermia" OR "azoospermia" OR "sertoli cell-only syndrome" OR "teratozoospermia" OR "semen quality" OR "semen volume" OR "sperm quality" OR "sperm parameter" OR "semen parameter" OR "sperm damage" OR "testicular"

#5 "reproductive endocrine" OR "hyperandrogenism" OR "gonadotropins" OR "GnRH" OR "goserelin" OR "hypogonadism" OR "hypothalamic-pituitary-gonadal axis" OR "follicle stimulating hormone" OR "dehydroepiandrosterone" OR "prolactin" OR "luteinizing hormone" OR "testosterone" OR "estradiol" OR "sex hormone-binding globulin" OR "Free Androgen Index" OR "FAI" OR "sex hormone" OR "androstenedione"

#6 "sexual behavior" OR "sexual dysfunctions, psychological" OR "erectile dysfunction" OR "premature ejaculation" OR "erectile disorder" OR "ejaculation dysfunction" OR "early ejaculation" OR "delayed ejaculation" OR "erectile difficulty" OR "sexual and gender disorders" OR "Sexual Function" OR "International Index of Erectile Function" OR "IIEF" OR "intravaginal ejaculatory latency time" OR "IELT" OR "ASEX" OR "Arizona Sexual Experience Scale" OR "CSFQ" OR "Changes in Sexual Functioning Questionnaire"

#7 "infertility, male" OR "Male Infertility" OR "Sterility, Male" OR "Male Sterility" OR "Subfertility, Male" OR "Male Subfertility" OR "Sub-Fertility, Male" OR "Male Sub-Fertility" OR "Sub Fertility, Male"

#8 (#4 OR #5 OR #6 OR #7) OR #3

#9 #1 OR #2

#10 #9 AND #8

#11 #10 NOT ("animal" OR "animal cell line" OR "animal experiment" OR "rat")

Table S3. Drug-name mapping for the 21 antiseizure medications (FAERS analysis)

Standardized ASM	Search term	Term type
**Brivaracetam**	BRIVARACETAM	Ingredient
	BRIVIACT	Brand
**Carbamazepine**	CARBAMAZEPINE	Ingredient
	CARBAGEN	Brand
	CARBATROL	Brand
	EPITOL	Brand
	EQUETRO	Brand
	TEGRETOL	Brand
	TERIL	Brand
	TIMONIL	Brand
	TRIMONIL	Brand
**Clobazam**	CLOBAZAM	Ingredient
	FRISIUM	Brand
	ONFI	Brand
	SYMPAZAN	Brand
	URBANOL	Brand
**Clonazepam**	CLONAZEPAM	Ingredient
	ANTELEPSIN	Brand
	KLONOPIN	Brand
	PAXAM	Brand
	RIVOTRIL	Brand
**Eslicarbazepine**	ESLICARBAZEPINE	Ingredient
	ESLICARBAZEPINE ACETATE	Salt
	APTIOM	Brand
	EXALIEF	Brand
	ZEBINIX	Brand
**Ethosuximide**	ETHOSUXIMIDE	Ingredient
	EMESIDE	Brand
	PETNIDAN	Brand
	SUXINUTIN	Brand
	ZARONTIN	Brand
**Felbamate**	FELBAMATE	Ingredient
	FELBATOL	Brand
	TALOXA	Brand
**Gabapentin**	GABAPENTIN	Ingredient
	GABAPENTIN ENACARBIL	Prodrug
	GABARONE	Brand
	GRALISE	Brand
	HORIZANT	Brand
	NEURONTIN	Brand
	NEUROSTIL	Brand
**Lacosamide**	LACOSAMIDE	Ingredient
	MOTPOLY	Brand
	VIMPAT	Brand
**Lamotrigine**	LAMOTRIGINE	Ingredient
	LAMEPTIL	Brand
	LAMICTAL	Brand

	LAMICTIN	Brand
	SUBVENITE	Brand
**Levetiracetam**	LEVETIRACETAM	Ingredient
	DESITREND	Brand
	ELEPSIA	Brand
	KEPPRA	Brand
	MATEVER	Brand
	ROWEEPRA	Brand
	SPRITAM	Brand
**Oxcarbazepine**	OXCARBAZEPINE	Ingredient
	APYDAN	Brand
	OXTELLAR	Brand
	TIMOX	Brand
	TRILEPTAL	Brand
**Perampanel**	PERAMPANEL	Ingredient
	FYCOMPA	Brand
**Phenobarbital**	PHENOBARB	Ingredient
	PHENOBARBITAL	Ingredient
	PHENOBARBITONE	Ingredient
	PHENOBARBITAL SODIUM	Salt
	GARDENAL	Brand
	LUMINAL	Brand
	SOLFOTON	Brand
**Phenytoin**	DIPHENYLHYDANTOIN	Ingredient
	PHENYTOIN	Ingredient
	PHENYTOIN SODIUM	Salt
	CEREBYX	Prodrug
	FOSPHENYTOIN	Prodrug
	SESQUIENT	Prodrug
	DILANTIN	Brand
	EPANUTIN	Brand
	EPTOIN	Brand
	INFATABS	Brand
	PHENYTEK	Brand
**Pregabalin**	PREGABALIN	Ingredient
	ALZAIN	Brand
	AXALID	Brand
	LYRICA	Brand
	REWISCA	Brand
**Rufinamide**	RUFINAMIDE	Ingredient
	BANZEL	Brand
	INOVELON	Brand
**Topiramate**	TOPIRAMATE	Ingredient
	EPRONTIA	Brand
	QUDEXY	Brand
	TOPAMAX	Brand
	TOPIMAX	Brand
	TROKENDI	Brand
	QSYMIA	Combo_Brand

**Valproate**	VALPROATE	Ingredient
	VALPROIC ACID	Ingredient
	DIVALPROEX	Salt
	DIVALPROEX SODIUM	Salt
	MAGNESIUM VALPROATE	Salt
	SEMISODIUM VALPROATE	Salt
	SODIUM VALPROATE	Salt
	VALPROATE CALCIUM	Salt
	VALPROATE MAGNESIUM	Salt
	VALPROATE SEMISODIUM	Salt
	VALPROATE SODIUM	Salt
	VALPROMIDE	Prodrug
	CONVULEX	Brand
	DEPAKENE	Brand
	DEPAKINE	Brand
	DEPAKOTE	Brand
	DEPAMIDE	Brand
	EPILIM	Brand
	EPIVAL	Brand
	ERGENYL	Brand
	MICROPAKINE	Brand
	ORFIRIL	Brand
	STAVZOR	Brand
	VALPARIN	Brand
**Vigabatrin**	VIGABATRIN	Ingredient
	SABRIL	Brand
	SABRILEX	Brand
	VIGADRONE	Brand
	VIGAFYDE	Brand
**Zonisamide**	ZONISAMIDE	Ingredient
	EXCEGRAN	Brand
	ZONEGRAN	Brand
	ZONISADE	Brand

ASM, antiseizure medication.

Search terms were matched against both the free-text drug name (DRUGNAME) and the active-ingredient field (PROD_AI), each normalized to uppercase with collapsed whitespace.

Table S4. Definition of reproductive disorders based on the MedDRA narrow-scope SMQ 'Fertility disorders' (version 27.0) (FAERS analysis)

SMQ	Narrow-scope PTs
**Fertility disorders**	Adiposogenital dystrophy (N), Adnexa uteri calcification (F), Androgens decreased (N), Anti-Muellerian hormone level decreased (F), Anti-sperm antibody positive (M), Antral follicle count low (F), Artificial insemination (N), Aspermia (M), Assisted fertilisation (N), Assisted reproductive technology (N), Asthenospermia (M), Autoimmune oophoritis (F), Azoospermia (M), Blood testosterone abnormal (N), Cervix stent placement (F), Controlled ovarian stimulation (F), Decreased embryo viability (F), Delayed follicular ripening (F), Diminished ovarian reserve (F), Ejaculatory duct cyst (M), Ejaculatory duct dilation procedure (M), Endometrial scratching (F), Endometrial thinning (F), Failed artificial insemination (N), Failed in vitro fertilisation (N), Fallopian tube adhesion (F), Fallopian tube congestion (F), Fallopian tube disorder (F), Fallopian tube diverticulosis (F), Fallopian tube obstruction (F), Fallopian tube spasm (F), Fallopian tube stenosis (F), Fallopian tube stent insertion (F), Follicle stimulating hormone deficiency (N), Free androgen index decreased (N), Fructose semen decreased (M), Fructose semen increased (M), Gamete intrafallopian transfer (F), Genital atrophy (N), Gonadotrophin deficiency (N), Habitual abortion (F), Haematospermia (M), High response to ovarian stimulation (F), Human chorionic gonadotropin abnormal (N), Human chorionic gonadotropin decreased (N), Human seminal plasma hypersensitivity (M), Hydrotubation (F), Hypergonadism (N), Hyperspermia (M), Hypogonadism (N), Hypogonadism female (F), Hypogonadism male (M), Hypospermia (M), In vitro fertilisation (N), Increased follicular atresia (F), Infertile partner (N), Infertility (N), Infertility female (F), Infertility male (M), Infertility tests abnormal (N), Low response to ovarian stimulation (F), Luteal phase deficiency (F), Luteinising hormone deficiency (N), Luteinising unruptured follicle syndrome (F), Macroorchidism (M), Microorchidism (M), Necrospermia (M), OHVIRA syndrome (F), Oestrogen deficiency (F), Oligoasthenoteratozoospermia (M), Oligoasthenozoospermia (M), Oligospermia (M), Ovarian atrophy (F), Ovarian drilling (F), Ovarian dysfunction (F), Ovarian failure (F), Ovarian failure postoperative (F), Ovarian hyperstimulation syndrome (F), Ovarioleukodystrophy (F), Ovulation delayed (F), Ovulation disorder (F), Ovulation induction (F), Polycystic ovarian syndrome (F), Polycystic ovaries (F), Poor response to ovulation induction (F), Premature follicular ripening (F), Premature menopause (F), Premature ovulation (F), Prevention of premature ovulation (F), Primary hypogonadism (N), Progesterone deficiency (F), Prostatic fluid leukocytes increased (M), Pyospermia (M), Radiation menopause (F), Red blood cells semen positive (M), Reproductive toxicity (N), Salpingo-ovariolysis (F), Secondary hypogonadism (N), Semen analysis abnormal (M), Semen discolouration (M), Semen liquefaction abnormal (M), Semen liquefaction prolonged (M), Semen liquefaction shortened (M), Semen viscosity abnormal (M), Semen viscosity decreased (M), Semen viscosity increased (M), Semen volume abnormal (M), Semen volume decreased (M), Semen volume increased (M), Seminal vesicle atrophy (M), Sperm analysis abnormal (M), Sperm aspiration (M), Sperm concentration abnormal (M), Sperm

	concentration decreased (M), Sperm concentration increased (M), Sperm concentration zero (M), Spermatogenesis abnormal (M), Spermatorrhoea (M), Spermatozoa abnormal (M), Spermatozoa morphology abnormal (M), Spermatozoa progressive motility abnormal (M), Spermatozoa progressive motility decreased (M), Stimulation of spermatogenesis (M), Surrogate mother (F), Teratospermia (M), Testicular atrophy (M), Testicular disorder (M), Testicular failure (M), Testicular failure postoperative (M), Testicular failure primary (M), Testicular infarction (M), Testicular injury (M), Testicular necrosis (M), Testicular rupture (M), Testosterone deficiency syndrome (N), Total sperm count decreased (M), Uterine peristalsis absent (F), Varicocele (M), White blood cells semen positive (M), pH semen abnormal (M), pH semen decreased (M), pH semen increased (M)

SMQ, Standardised MedDRA Query; PT, preferred term.

Among the 145 narrow-scope PTs comprising the SMQ 'Fertility disorders' (MedDRA v27.0), three PTs (Chromosome analysis abnormal, Karyotype analysis abnormal, and Philadelphia chromosome positive) were excluded as nonspecific findings with unclear subject attribution; thus, 142 PTs were ultimately included. Of these, 64 are male-specific (M), 53 are female-specific (F), and 25 are sex-neutral (N). The primary case definition for this study comprised the 89 male-specific and sex-neutral PTs (M+N), excluding the 53 female-specific PTs given the male-only cohort restriction; a sensitivity analysis restricted to the 64 male-specific PTs only yielded a stronger valproate signal (ROR 6.181, 95% CI 3.776 to 10.118; IC_{025} 1.911), consistent with the primary analysis.

Table S5. Characteristics of included studies (Meta-analysis)

Study (year)	Country	Study design	Total (n)	Case (n)	Control (n)	Age (mean)	Data type	AED treatment (n)	Outcome
Ceylan et al. (2016)	Turkey	before-after	26	26	U (26)	28.6	paired	levetiracetam (26)	PRL, FSH, LH, total T, E2, total sperm count, normal morphology sperm (%), sperm concentration
Duncan et al. (1999)	England	cross-sectional	135	70	U (32), H (33)	30.8	independent	carbamazepine (31), phenytoin (21), valproate (18)	total T, free T, E2, SHBG, FAI, DHEA-S
Geisler et al. (1997)	Norway	before-after	21	21	U (21)	19.1	paired	carbamazepine (15), valproate (6)	FSH, LH, total T, SHBG, DHEA-S
Guo et al. (2021)	China	controlled before-after	74	44	U (44), H (30)	27.3	independent, paired	oxcarbazepine (21), valproate (23)	PRL, FSH, LH, total T, E2, progressive motility (%), total sperm count, normal morphology sperm (%), sperm concentration, IIEF-5
Hamed et al. (2015)	Egypt	cross-sectional	58	28	H (30)	31.3	independent	valproate (28)	free T, progressive motility (%), total sperm count, semen volume, sperm concentration
Heroz et al. (1991)	USA	cross-sectional	61	20	U (21), H (20)	33.1	independent	phenytoin (20)	E2
Isojärvi et al. (1990)	Finland	cross-sectional	61	44	H (17)	33.4	independent	carbamazepine (20), phenytoin (17), valproate (7)	PRL, FSH, LH, total T, free T, E2, SHBG, FAI, DHEA-S
Isojärvi et al. (1995)	Finland	prospective cohort, cross-sectional	54	36	H (18)	33.7	independent, paired	carbamazepine (25), phenytoin (11)	total T, SHBG, FAI

Mohamed et al. (2019)	Egypt	before-after	50	50	U (50)	31.2	independent	levetiracetam (25), valproate (25)	PRL, FSH, LH, total T, E2, total sperm count, normal morphology sperm (%), sperm concentration
Nag et al. (1997)	India	controlled before-after	46	26	H (20)	27.3	independent, paired	valproate (26)	PRL, FSH, LH, total T
Najafi et al. (2012)	Iran	cross-sectional	73	50	H (23)	23.9	independent	carbamazepine (18), lamotrigine (17), valproate (15)	PRL, FSH, LH, total T, E2, SHBG, DHEA-S
Ocek et al. (2018)	Turkey	cross-sectional	89	59	H (30)	30.2	independent	carbamazepine (29), valproate (30)	PRL, FSH, LH, total T, free T, E2, SHBG, DHEA-S, head, neck/midpiece, and tail abnormalities (%), normal morphology sperm (%), semen volume, sperm concentration, IIEF-5
Rättyä et al. (2001) (A)	Finland	before-after	22	22	U (22)	31.6	paired	carbamazepine (10), valproate (12)	FSH, LH, total T, SHBG, FAI, DHEA-S
Rättyä et al. (2001) (B)	Finland	cross-sectional	115	90	H (25)	32.9	independent	carbamazepine (40), oxcarbazepine (29), valproate (21)	FSH, LH, total T, SHBG, DHEA-S
Reis et al. (2013)	Brazil	cross-sectional	118	63	H (55)	31.6	independent	carbamazepine (63)	PRL, LH, total T, SHBG, FAI, DHEA-S
Røste et al. (2003)	Norway	cross-sectional	126	36	H (90)	31.6	independent	carbamazepine (20), valproate (16)	progressive motility (%), semen volume, sperm concentration, head, neck/midpiece, and tail abnormalities (%)
Røste et al. (2005)	Norway	cross-sectional	70	35	H (35)	29.7	independent	carbamazepine (19), valproate (16)	FSH, LH, total T, SHBG, DHEA-S

Svalheim et al. (2009)	Norway	cross-sectional	166	130	H (36)	31.6	independent	carbamazepine (63), lamotrigine (37), levetiracetam (30)	FSH, LH, total T, free T, E2, SHBG, FAI, DHEA-S
Taneja et al. (1994)	India	cross-sectional	83	42	U (13), H (28)	28.3	independent	phenytoin (42)	head, neck/midpiece abnormalities (%), total sperm count, semen volume, sperm concentration
Xiaotian et al. (2013)	China	cross-sectional	82	52	H (30)	29	independent	levetiracetam (20), valproate (32)	PRL, FSH, LH, total T, E2, progressive motility (%), head, neck/midpiece, and tail abnormalities (%), sperm concentration, IIEF-5

AED, antiepileptic drugs; H, healthy controls, U, untreated patients

Clinical endpoints were categorized into three groups: **(i) sperm parameters:** progressive motility (%), head, neck/midpiece, and tail abnormalities (%), total sperm count ($\times 10^6$/ejaculation), normal morphology sperm (%), semen volume (ml), and sperm concentration ($\times 10^6$/ml); **(ii) sex hormones and related indicators:** dehydroepiandrosterone sulfate (DHEA-S, µl/dL), estradiol (E2, pmol/L), follicle-stimulating hormone (FSH, mIU/mL), luteinizing hormone (LH, mIU/mL), prolactin (PRL, mIU/L), total testosterone (T, nmol/L), Free T (pmol/L), sex hormone-binding globulin (SHBG, nmol/L), free androgen index (FAI); **(iii) sexual dysfunction** assessed via the International Index of Erectile Function-5 (IIEF-5) questionnaire score (points).

Table S6. Quality assessment of cross-sectional studies using the AXIS tool (Meta-analysis)

Items	Study numbers*													
	1	2	3	4	5	6	7	8	9	10	11	12	13	14
**Introduction**														
1. Were the aims/objectives of this study clear?	Y	Y	Y	Y	Y	Y	Y	Y	Y	Y	Y	Y	Y	Y
**Methods**														
2. Was the study design appropriate for the stated aims?	Y	Y	Y	Y	Y	Y	Y	Y	Y	Y	Y	Y	Y	Y
3. Was the sample size justified?	N	N	N	N	N	N	N	N	N	N	N	N	Y	N
4. Was the target/reference population clearly defined? (Is it clear who the research was about?)	Y	Y	Y	Y	Y	Y	Y	Y	Y	Y	Y	Y	Y	Y
5. Was the sample frame taken from an appropriate base so that it closely represented the target/reference population under investigation?	Y	Y	Y	Y	Y	Y	Y	Y	Y	Y	Y	Y	Y	Y
6. Was the selection process likely to select subjects/participants that were representative of the target/reference population under investigation?	Y	Y	Y	Y	Y	Y	Y	Y	Y	Y	Y	Y	Y	Y
7. Were measures undertaken to address and categorise non-responders?	N/A	N/A	N/A	N/A	N/A	N/A	N/A	N/A	N/A	N/A	N/A	N/A	N/A	N/A
8. Were the risk factor and outcome variables measured appropriate to the aims of the study?	Y	Y	Y	Y	Y	Y	Y	Y	Y	Y	Y	Y	Y	Y
9. Were the risk factor and outcome varables measured correctly using instruments/measurements that had been trialed, piloted or published previously?	Y	Y	Y	Y	Y	Y	Y	Y	Y	Y	Y	Y	Y	Y

10. Is it clear what was used to determine statistical significance and/or precision estimates? (e.g. p-values, confidence, intervals)	Y	Y	Y	Y	Y	Y	Y	Y	Y	Y	Y	Y	Y	Y
11. Were the methods (including statistical methods) sufficiently described to enable them to be repeated?	Y	Y	Y	Y	Y	Y	Y	Y	Y	Y	Y	Y	Y	Y
**Results**														
12. Were the basic data adequately described?	Y	Y	Y	Y	Y	Y	Y	Y	Y	Y	Y	Y	Y	Y
13. Does the response rate raise concerns about non-response bias?	N/A	N/A	N/A	N/A	N/A	N/A	N/A	N/A	N/A	N/A	N/A	N/A	N/A	N/A
14. If appropriate, was information about non-responders described?	N/A	N/A	N/A	N/A	N/A	N/A	N/A	N/A	N/A	N/A	N/A	N/A	N/A	N/A
15. Were the results internally consistent?	Y	Y	Y	Y	Y	Y	Y	Y	Y	Y	Y	Y	Y	Y
16. Were the results presented for all analyes described in the methods?	Y	Y	Y	Y	Y	Y	Y	Y	Y	Y	Y	Y	Y	Y
**Discussion**														
17. Were the authors' discussions and conclusions justified by the results?	Y	Y	Y	Y	Y	Y	Y	Y	Y	Y	Y	Y	Y	Y
18. Were the limitations of the study discussed?	Y	Y	Y	Y	Y	N	Y	Y	Y	Y	Y	Y	Y	Y
**Other**														
19. Were there any funding sources or conflicts of interest that may affect the authors' interpretation of the results?	N/A	N	N/A	N/A	N/A	N/A	N	N/A	N	N	N	N	N	N
20. Was ethical approval or consent of participants attained?	N	Y	N	Y	Y	Y	Y	Y	Y	Y	Y	Y	Y	Y
**Total appraisal score**	14	15	14	15	15	14	15	15	15	15	15	15	16	15
**Study quality**	F	F	F	F	F	F	F	F	F	F	F	F	F	F

F, fair; N, no; N/A, not available; Y, yes

* Study number, author, and year of publication: 1 Duncan 1999; 2 Hamed 2015; 3 Herzog 1991; 4 Isojärvi 1990; 5 Isojärvi 1995; 6 Najafi 2012; 7 Ocek 2018; 8 Rättyä (B) 2001; 9 Reis 2013;10 Røste (A) 2003; 11 Røste (B) 2005; 12 Svalheim 2009; 13 Taneja 1994; 14 Xiaotian 2013

Table S7. Quality assessment of before–after and prospective cohort studies using an adapted NOS tool (Meta-analysis)

Study Author (year)	Selection			Comparability	Outcome			No. of 'A's
	Representativeness of the exposed cohort	Selection of the non-exposed cohort	Ascertainment of exposure	Comparability of cohorts	Ascertainment of outcome	Was follow-up long enough	Adequacy of follow up of cohorts	
	A. truly representative (all eligible exposed cohort within specific organization and time period) B. somewhat representative C. selected group of users D. no description	A. from the same population as the exposed cohort B. from a different source C. no description	A. secure record B. structured interview C. written self report D. no description	A. study controls for sex B. study controls for comorbidity	A. medical record B. record linkage C. self report D. no description	A. yes B. no	A. complete follow up B. small number lost C. low follow up rate and no description of those lost D. no statement	
Ceylan et al. (2016)	B	A	A	AB	A	A	B	5
Geisler et al. (1997)	B	A	A	A	A	A	A	6
Guo et al. (2021)	B	A	A	AB	A	A	A	6
Isojärvi et al. (1995)	B	A	A	A	A	A	C	5
Mohamed et al. (2019)	A	A	A	AB	A	B	A	6
Nag et al. (1997)	D	A	A	A	A	A	D	5
Rättyä et al. (2001) (A)	B	A	A	AB	A	A	A	6

Table S8. Data extracted: before-after studies and a prospective cohort study (Meta-analysis)

Study (year)	ASM	Clinical endpoint	Case (after)			Control (before)			Control (H/U)			P value
			Mean	SD or 95% CI	N	Mean	SD or 95% CI	N	Mean	SD	N	
Ceylan et al. (2016)	LEV	PRL	206.4	168.1–242.6	26	208.5	178.7–240.4	26	N/A	N/A	N/A	0.726
		FSH	8.5	6.8–10.1	26	8.7	6.9–10.4	26	N/A	N/A	N/A	0.841
		LH	15.4	12.3–18.6	26	13.9	11.2–16.6	26	N/A	N/A	N/A	0.262
		total T	16.4	13.6–19.2	26	15.2	12.7–17.6	26	N/A	N/A	N/A	0.491
		E2	88.9	76.7–101.0	26	83.3	72.0–95.1	26	N/A	N/A	N/A	0.625
		total sperm count	266.5	222.5–310.4	26	304.5	257.1–351.8	26	N/A	N/A	N/A	<0.001
		normal morphology sperm (%)	26.5	23.6–29.3	26	28.9	25.9–31.8	26	N/A	N/A	N/A	<0.001
		sperm concentration	77.0	66.6–87.3	26	85.7	74.1–97.4	26	N/A	N/A	N/A	<0.001
Geisler et al. (1997)	CBZ	FSH	3.8	2.9–5.0	15	3.8	2.9–4.8	15	N/A	N/A	N/A	N/A
		LH	4	3.3–4.9	15	4.2	3.1–5.7	15	N/A	N/A	N/A	N/A
		total T	15.9	12.5–20.3	15	17.6	13.8–22.4	15	N/A	N/A	N/A	N/A
		SHBG	31.3	25–39.3	15	27.7	24.7–31.1	15	N/A	N/A	N/A	N/A
		DHEA-S	3	2.1–4.4	15	4.9	3.3–7.2	15	N/A	N/A	N/A	<0.001
	VPA	FSH	2.7	1.5–4.8	6	3.3	2.1–5.2	6	N/A	N/A	N/A	<0.05
		LH	3.2	2.1–4.9	6	3.6	2.4–5.6	6	N/A	N/A	N/A	N/A
		total T	18	14.2–22.9	6	17.9	14.2–22.6	6	N/A	N/A	N/A	N/A
		SHBG	26.8	23.1–31.1	6	28.5	23.4–34.6	6	N/A	N/A	N/A	N/A
		DHEA-S	5.5	3.5–8.7	6	5.3	3.5–8.2	6	N/A	N/A	N/A	N/A
Guo et al. (2021)	VPA	PRL	282.2	103.7	23	226.3	57.5	23	U 213.6	47.0	30	0.029
		FSH	2.8	0.8	23	3.0	0.9	23	U 3.2	1.1	30	N/A
		LH	2.6	0.9	23	2.8	0.9	23	U 2.8	1.3	30	N/A
		total T	18.7	5.6	23	18.8	6.2	23	U 18.3	6.1	30	N/A
		E2	114.9	26.6	23	109.6	30.2	23	U 109.8	33.1	30	N/A

		progressive motility (%)	34.7	8.2	23	42.0	4.6	23	U 44.0	5.7	30	0.001
		total sperm count	97.3	42.3	23	130.4	43.8	23	U 157.9	41.2	30	0.012
		normal morphology sperm (%)	2.3	0.8	23	2.6	0.7	23	U 2.6	0.9	30	N/A
		sperm concentration	30	9.2	23	37.7	7.6	23	U 41.77	7.19	30	0.003
		IIEF-5	18.1	2.6	23	19.1	2.8	23	U 21.7	3.64	30	N/A
	OXC	PRL	209.5	73.1	21	232.6	67.4	21	U 213.6	47.0	30	N/A
		FSH	4.2	1.7	21	3.1	1.0	21	U 3.2	1.1	30	0.014
		LH	3.5	1.7	21	2.6	1.3	21	U 2.8	1.3	30	N/A
		total T	20.0	7.1	21	18.1	4.9	21	U 18.3	6.1	30	N/A
		E2	113.6	21.5	21	112.9	33.6	21	U 109.8	33.1	30	N/A
		progressive motility (%)	44.7	4.8	21	42.7	3.0	21	U 44.0	5.7	30	N/A
		total sperm count	125.6	35.5	21	121.0	39.5	21	U 157.9	41.2	30	N/A
		normal morphology sperm (%)	2.9	0.6	21	2.5	0.9	21	U 2.6	0.9	30	N/A
		sperm concentration	37.6	0.6	21	36.4	5.2	21	U 41.77	7.19	30	N/A
		IIEF-5	20.0	2.1	21	19.0	2.6	21	U 21.7	3.64	30	N/A
Isojärvi et al. (1995)	CBZ	total T	25.7	6.6	11	24.6	6.6	11	N/A	N/A	N/A	N/A
		SHBG	47.9	22.2	11	28.0	9.3	11	N/A	N/A	N/A	<0.01
		FAI	58.7	16.9	11	98.3	47.4	11	N/A	N/A	N/A	<0.01
Mohamed et al. (2019)	VPA	PRL	312.8	178.7	25	180.9	85.1	25	N/A	N/A	N/A	<0.001
		FSH	4.6	2.8	25	6.0	2.6	25	N/A	N/A	N/A	0.01
		LH	3.4	1.37	25	4.9	1.8	25	N/A	N/A	N/A	0.01
		total T	18.7	6.24	25	20.1	6.9	25	N/A	N/A	N/A	0.1
		E2	90.3	40.0	25	93.3	41.5	25	N/A	N/A	N/A	0.9

		total sperm count	37.6	9.1	25	45.6	9.0	25	N/A	N/A	N/A	0.01
		normal morphology sperm (%)	2.9	1.1	25	3.4	1.4	25	N/A	N/A	N/A	0.029
		sperm concentration	25.0	12.1	25	29.9	12.8	25	N/A	N/A	N/A	0.01
	LEV	PRL	170.2	76.6	25	184.0	89.4	25	N/A	N/A	N/A	0.12
		FSH	5.1	3.3	25	6.4	3.2	25	N/A	N/A	N/A	0.11
		LH	3.7	1.9	25	4.7	2	25	N/A	N/A	N/A	0.07
		total T	17.3	6.8	25	19.8	5.6	25	N/A	N/A	N/A	0.07
		E2	90.3	42.2	25	92.2	43.0	25	N/A	N/A	N/A	0.6
		total sperm count	38.8	8.3	25	45.6	9.1	25	N/A	N/A	N/A	0.01
		normal morphology sperm (%)	5.1	1.5	25	6.3	1.8	25	N/A	N/A	N/A	0.01
		sperm concentration	23.2	10.3	25	29.1	12.6	25	N/A	N/A	N/A	0.01
Nag et al. (1997)	VPA	PRL	384.1	101.5	26	389.8	143.0	26	H 416.0	138.6	20	N/A
		FSH	5.0	0.9	26	4.4	1.1	26	H 4.2	1.3	20	N/A
		LH	6.5	1.9	26	7.8	1.9	26	H 8.3	1.6	20	N/A
		total T	20.3	3.7	26	24.7	7.3	26	H 29.1	6.7	20	N/A
Rättyä et al. (2001) (A)	VPA	FSH	3.2	1.5	12	4.0	1.9	12	N/A	N/A	N/A	<0.005
		LH	4.1	1.7	12	5.1	2.8	12	N/A	N/A	N/A	<0.1
		total T	29.3	10.6	12	27.2	11.3	12	N/A	N/A	N/A	N/A
		SHBG	44.7	25.3	12	41.3	28.5	12	N/A	N/A	N/A	N/A
		FAI	71.7	17.3	12	73.0	21.4	12	N/A	N/A	N/A	N/A
		DHEA-S	6.5	3.1	12	6.9	2.0	12	N/A	N/A	N/A	N/A
	CBZ	FSH	4.8	3.3	10	4.6	3.8	10	N/A	N/A	N/A	N/A
		LH	5.0	1.7	10	4.7	1.6	10	N/A	N/A	N/A	N/A
		total T	25.9	8.8	10	23.6	7.5	10	N/A	N/A	N/A	N/A
		SHBG	39.5	11.7	10	30.9	10.1	10	N/A	N/A	N/A	<0.001
		FAI	67.7	20.6	10	81.6	32.5	10	N/A	N/A	N/A	<0.1

	DHEA-S	3.7	2.4	10	7.2	3.6	10	N/A	N/A	N/A	<0.005

ASM, antiseizure medication; CBZ, carbamazepine; CI, confidence interval; H, healthy control; LEV, levetiracetam; LTG, lamotrigine; N, number of participants; N/A, not available; OXC, oxcarbazepine; PHT, phenytoin; SD, standard deviation; U, untreated patients; VPA, valproate

Clinical endpoints were categorized into three groups: **(i) sperm parameters:** progressive motility (%), head, neck/midpiece, and tail abnormalities (%), total sperm count ($\times 10^6$/ejaculation), normal morphology sperm (%), semen volume (ml), and sperm concentration ($\times 10^6$/ml); **(ii) sex hormones and related indicators:** dehydroepiandrosterone sulfate (DHEA-S, μl/dL), estradiol (E2, pmol/L), follicle-stimulating hormone (FSH, mIU/mL), luteinizing hormone (LH, mIU/mL), prolactin (PRL, mIU/L), total testosterone (T, nmol/L), Free T (pmol/L), sex hormone-binding globulin (SHBG, nmol/L), free androgen index (FAI); **(iii) sexual dysfunction**, assessed via the International Index of Erectile Function-5 (IIEF-5) questionnaire score (points).

Values are reported to no more than three decimal places, based on the precision of the original publications.

Table S9. Data extracted: cross-sectional studies (Meta-analysis)

Study (year)	ASM	Clinical endpoint	Case			Control (H/U)			P value
			Mean	SD or 95% CI	N	Mean	SD or 95% CI	N	
Duncan et al. (1999)	VPA	total T	16.2	12.7–19.6	18	H 16.6	14.8–18.4	33	N/A
						U 19.3	17.4–21.1	32	N/A
		free T	395.9	323.9–467.9	18	H 467.7	413.4–522.0	33	N/A
						U 488.6	446.9–530.4	32	N/A
		E2	155.8	121.6–190.1	18	H 154.8	133.8–175.8	33	N/A
						U 180.5	137.9–223.2	32	N/A
		SHBG	35.6	26.5–44.6	18	H 26.2	21.8–30.4	33	N/A
						U 32.8	28.0–37.5	32	N/A
		FAI	16.4	7.0–25.9	18	H 24.9	15.7–34.0	33	N/A
						U 18.2	14.0–22.3	32	N/A
		DHEA-S	10.2	7.5–13.0	18	H 9	6.8–11.2	33	N/A
						U 9.4	7.5–11.2	32	N/A
	CBZ	total T	18.7	16.5–20.9	31	H 16.6	14.8–18.4	33	N/A
						U 19.3	17.4–21.1	32	N/A
		free T	408.2	357.7–458.6	31	H 467.7	413.4–522.0	33	N/A
						U 488.6	446.9–530.4	32	N/A
		E2	153.6	125.1–182.3	31	H 154.8	133.8–175.8	33	N/A
						U 180.5	137.9–223.2	32	N/A
		SHBG	49.1	37.4–60.8	31	H 26.2	21.8–30.4	33	<0.01
						U 32.8	28.0–37.5	32	N/A
		FAI	11.4	8.4–14.4	31	H 24.9	15.7–34.0	33	<0.01
						U 18.2	14.0–22.3	32	N/A
		DHEA-S	5.6	4.2–6.9	31	H 9	6.8–11.2	33	<0.01
						U 9.4	7.5–11.2	32	<0.01
	PHT	total T	24.1	19.5–28.2	21	H 16.6	14.8–18.4	33	<0.01
						U 19.3	17.4–21.1	32	N/A
		free T	471.4	411.9–530.9	21	H 467.7	413.4–522.0	33	N/A
						U 488.6	446.9–530.4	32	N/A
		E2	198	151.2–242.5	21	H 154.8	133.8–175.8	33	N/A
						U 180.5	137.9–223.2	32	N/A

		SHBG	56.2	42.2–70.2	21	H 26.2	21.8–30.4	33	<0.01
						U 32.8	28.0–37.5	32	<0.01
		FAI	12.7	6.8–18.7	21	H 24.9	15.7–34.0	33	<0.01
						U 18.2	14.0–22.3	32	<0.01
		DHEA-S	4.4	2.8–6.0	21	H 9	6.8–11.2	33	<0.01
						U 9.4	7.5–11.2	32	<0.01
Hamed et al. (2015)	VPA	progressive motility (%)	25.9	5.7	28	H 75.6	5.05	30	<0.001
		total sperm count	45.6	15.2	28	H 88.8	16.0	30	<0.001
		semen volume	2.6	0.4	28	H 2.4	0.5	30	N/A
		sperm concentration	15.3	5.5	28	H 25.6	3.45	30	<0.001
Herzog et al. (1991)	PHT	E2	206.7	107.9	20	H 125.9	46.6	20	<0.05
						U 119.0	100.6	21	<0.01
Isojärvi et al. (1990)	CBZ	PRL	172.3	80.8	20	H 202.1	85.1	17	N/A
		FSH	4.6	2.1	20	H 6.2	5.7	17	N/A
		LH	7.8	1.7	20	H 8.0	1.3	17	N/A
		total T	23.5	7.3	20	H 23.3	8.6	17	N/A
		free T	93.0	19.3	20	H 91.3	22.3	17	N/A
		E2	200.0	70.0	20	H 150.0	80.0	17	N/A
		SHBG	36.7	13.5	20	H 27.2	7.9	17	N/A
		FAI	67.7	16.8	20	H 86.6	22.4	17	<0.05
		DHEA-S	4.3	1.5	20	H 8.0	3.9	17	<0.001
	PHT	PRL	108.5	63.8	17	H 202.1	85.1	17	<0.01
		FSH	4.8	1.9	17	H 6.2	5.7	17	N/A
		LH	6.6	2.4	17	H 8.0	1.3	17	<0.05
		total T	32.4	9.1	17	H 23.3	8.6	17	<0.01
		free T	91.0	20.4	17	H 91.3	22.3	17	N/A
		E2	220.0	80.0	17	H 150.0	80.0	17	<0.05
		SHBG	57.4	24.8	17	H 27.2	7.9	17	<0.001
		FAI	61.4	19.2	17	H 86.6	22.4	17	<0.01
		DHEA-S	3.4	1.6	17	H 8.0	3.9	17	<0.001
	VPA	PRL	183.0	66.0	7	H 202.1	85.1	17	N/A
		FSH	2.4	0.9	7	H 6.2	5.7	17	<0.01

		LH	4.5	0.6	7	H 8.0	1.3	17	<0.001
		total T	22.9	6.6	7	H 23.3	8.6	17	N/A
		free T	91.4	15.9	7	H 91.3	22.3	17	N/A
		E2	250.0	100.0	7	H 150.0	80.0	17	<0.01
		SHBG	24.2	11.1	7	H 27.2	7.9	17	N/A
		FAI	108	42.1	7	H 86.6	22.4	17	<0.05
		DHEA-S	9.8	5.3	7	H 8.0	3.9	17	N/A
Isojärvi et al. (1995)	CBZ	total T	23.2	6.6	14	H 23.2	8.3	18	N/A
		SHBG	53.7	25.7	14	H 26.9	8.2	18	<0.001
		FAI	47.9	13.9	14	H 86.5	21.8	18	<0.001
	PHT	total T	33.3	12.1	11	H 23.2	8.3	18	N/A
		SHBG	57.3	23.4	11	H 26.9	8.2	18	<0.001
		FAI	60.6	9.2	11	H 86.5	21.8	18	<0.01
Najafi et al. (2012)	VPA	PRL	192.9	87.5	15	H 220.8	77	23	N/A
		FSH	4.3	1.9	15	H 4.3	1.53	23	N/A
		LH	2.6	0.9	15	H 2.8	0.9	23	N/A
		total T	3.3	1.9	15	H 6.6	1.9	23	N/A
		E2	157.0	18	15	H 155.3	10.4	23	N/A
		SHBG	39.7	10.3	15	H 26.7	9.9	23	N/A
		DHEA-S	4.9	1.4	15	H 4.7	1.1	23	N/A
	CBZ	PRL	229.0	67.2	18	H 220.8	77.0	23	N/A
		FSH	4.5	1.2	18	H 4.3	1.5	23	N/A
		LH	3.6	0.9	18	H 2.8	0.9	23	N/A
		total T	3.6	1.7	18	H 6.6	1.9	23	N/A
		E2	150.6	11.6	18	H 155.3	10.4	23	N/A
		SHBG	41.1	21.5	18	H 26.7	9.9	23	N/A
		DHEA-S	2.3	1.1	18	H 4.7	1.1	23	N/A
	LTG	PRL	189.6	105.7	17	H 220.8	77.0	23	N/A
		FSH	3.1	1.0	17	H 4.3	1.5	23	<0.05
		LH	2.9	0.9	17	H 2.8	0.9	23	N/A
		total T	3.3	1.8	17	H 6.6	1.9	23	<0.05
		E2	153.2	17.1	17	H 155.3	10.4	23	N/A
		SHBG	20.3	11.2	17	H 26.7	9.9	23	N/A
		DHEA-S	4.1	1.1	17	H 4.7	1.1	23	N/A

Ocek et al. (2018)	VPA	PRL	243.2	110.6	30	H 213.6	75.5	30	0.825
		FSH	3.2	2.2	30	H 3.4	1.5	30	0.986
		LH	4.7	2.9	30	H 4.4	1.4	30	0.943
		total T	112.2	49.6	30	H 117.0	33.2	30	0.911
		free T	30095.0	11996.4	30	H 38242.8	11996.4	30	0.038
		E2	118.3	41.8	30	H 57.2	26.5	30	<0.001
		SHBG	34.4	27.4	30	H 26.5	5.2	30	0.274
		DHEA-S	9.0	5.0	30	H 5.8	2.2	30	0.014
		Head abnormalities (%)	77.0	8.2	24	H 77.9	3.2	29	0.918
		neck/midpiece abnormalities (%)	11.5	5.0	24	H 10.2	2.2	29	0.599
		tail abnormalities (%)	8.1	4.8	24	H 6.5	2.6	29	0.242
		normal morphology sperm (%)	3.6	1.9	24	H 5.4	1.5	29	0.003
		semen volume	2.0	1.4	24	H 3.7	1.4	29	<0.001
		sperm concentration	47.3	35.0	24	H 61.0	40.8	29	0.452
		IIEF-5	18.2	3.8	24	H 22.7	2.0	29	<0.001
	CBZ	PRL	332.3	295.7	29	H 213.6	75.5	30	0.05
		FSH	5.5	8.3	29	H 3.4	1.5	30	0.271
		LH	6.0	4.8	29	H 4.4	1.4	30	0.192
		total T	117.7	50.7	29	H 117.0	33.2	30	0.999
		free T	31481.9	13556.6	29	H 38242.8	11996.4	30	0.103
		E2	104.6	36.5	29	H 57.2	26.5	30	<0.001
		SHBG	37.5	18.2	29	H 26.5	5.2	30	0.086
		DHEA-S	7.2	3.9	29	H 5.8	2.2	30	0.440
		Head abnormalities (%)	75.8	11.6	20	H 77.9	3.2	29	0.632
		neck/midpiece abnormalities (%)	12.3	7.0	20	H 10.2	2.2	29	0.303
		tail abnormalities (%)	7.4	2.7	20	H 6.5	2.6	29	0.692

		normal morphology sperm (%)	3.1	2.4	20	H 5.41	1.52	29	<0.001
		semen volume	1.9	0.9	20	H 3.72	1.41	29	<0.001
		sperm concentration	50.0	52.6	20	H 61.00	40.79	29	0.628
		IIEF-5	18.6	2.9	20	H 22.72	2.01	29	<0.001
Rättyä et al. (2001) (B)	VPA	FSH	2.7	1.0	21	H 4.4	2.2	25	N/A
		LH	3.4	1.6	21	H 4.0	1.3	25	N/A
		total T	19.4	9.4	21	H 19.8	6.2	25	N/A
		SHBG	33.7	14.7	21	H 33.7	15.8	25	N/A
		DHEA-S	7.9	4.34	21	H 7.6	2.71	25	N/A
	CBZ	FSH	5.5	4.1	40	H 4.4	2.2	25	N/A
		LH	4.4	1.8	40	H 4.0	1.3	25	N/A
		total T	22.9	10.4	40	H 19.8	6.2	25	N/A
		SHBG	44.2	21.1	40	H 33.7	15.8	25	<0.05
		DHEA-S	4.3	2.7	40	H 7.6	2.7	25	<0.001
	OXC	FSH	6.5	5.9	29	H 4.4	2.2	25	N/A
		LH	5.2	3.9	29	H 4.0	1.3	25	N/A
		total T	24.3	7.3	29	H 19.8	6.2	25	N/A
		SHBG	41.1	16.8	29	H 33.7	15.8	25	N/A
		DHEA-S	7.3	2.2	29	H 7.6	2.7	25	N/A
Reis et al. (2013)	CBZ	PRL	240.4	153.2	63	H 206.4	100.0	55	0.9
		LH	5.4	4.4	63	H 2.5	1.2	55	<0.01
		total T	20.8	7.0	63	H 15.0	0.4	55	<0.01
		SHBG	48.3	22.6	63	H 22.1	9.7	55	<0.01
		FAI	48.3	19.3	63	H 78.1	34.3	55	<0.01
		DHEA-S	2.1	49.7	63	H 4.5	2.1	55	<0.01
Røste et al. (2003)	VPA	progressive motility (%)	48.5	10.9	16	H 53.4	7.9	90	N/A
		semen volume	3.9	1.2	16	H 4.2	2.0	90	N/A
		sperm concentration	78.9	80.4	16	H 88.7	68.3	90	N/A
		head abnormalities (%)	98.0	3.3	16	H 95.5	3.1	90	<0.001
		neck/midpiece abnormalities (%)	53.9	11.7	16	H 63.4	9.4	90	<0.001

		tail abnormalities (%)	11.3	5.2	16	H 7.1	4.4	90	<0.006
	CBZ	progressive motility (%)	49.9	10.8	20	H 53.4	7.9	90	N/A
		semen volume	4.6	1.9	20	H 4.2	2.0	90	N/A
		sperm concentration	64.5	41.3	20	H 88.7	68.3	90	N/A
		head abnormalities (%)	98.0	1.4	20	H 95.5	3.1	90	<0.001
		neck/midpiece abnormalities (%)	53.3	9.2	20	H 63.4	9.4	90	<0.001
		tail abnormalities (%)	9.5	7.1	20	H 7.1	4.4	90	N/A
Røste et al. (2005)	VPA	FSH	2.1	1.5	16	H 4.0	1.6	35	<0.01
		LH	2.4	1.1	16	H 3.6	2.0	35	<0.01
		total T	16.5	9.4	16	H 20.8	8.2	35	N/A
		SHBG	23.0	16.3	16	H 30.8	16.6	35	N/A
		DHEA-S	10.1	7.9	16	H 8.6	3.2	35	<0.01
	CBZ	FSH	3.1	1.8	19	H 4.0	1.6	35	N/A
		LH	3.6	2.3	19	H 3.6	2.0	35	N/A
		total T	15.4	5.1	19	H 20.8	8.2	35	N/A
		SHBG	33.1	19.9	19	H 30.8	16.6	35	N/A
		DHEA-S	7.2	3.8	19	H 8.6	3.2	35	N/A
Svalheim et al. (2009)	LEV	FSH	3.2	1.7	30	H 3.2	2.2	36	N/A
		LH	4.0	3.5	30	H 4.3	2.1	36	N/A
		total T	19.4	15.3	30	H 25.3	9.7	36	N/A
		free T	408.6	219.9	30	H 524.4	223.6	36	0.025
		E2	110	50	30	H 100.0	40.0	36	N/A
		SHBG	31.0	13.0	30	H 29.0	13.0	36	N/A
		FAI	64.0	24.8	30	H 76.0	34.5	36	N/A
		DHEA-S	7.2	5.2	30	H 7.3	2.8	36	N/A
	CBZ	FSH	4.9	5.2	63	H 3.2	2.2	36	N/A
		LH	5.6	3.6	63	H 4.3	2.1	36	<0.001
		total T	20.4	7.9	63	H 25.3	9.7	36	<0.001
		free T	346.5	160.0	63	H 524.4	223.6	36	<0.001

		E2	100.0	50.0	63	H 100.0	40.0	36	N/A
		SHBG	42.0	26.0	63	H 29.0	13.0	36	<0.001
		FAI	47.8	29.5	63	H 76.0	34.5	36	<0.001
		DHEA-S	4.3	2.1	63	H 7.3	2.8	36	<0.001
	LTG	FSH	3.9	3.4	37	H 3.2	2.2	36	N/A
		LH	4.1	2.0	37	H 4.3	2.1	36	N/A
		total T	21.0	8.5	37	H 25.3	9.7	36	N/A
		free T	424.3	185.4	37	H 524.4	223.6	36	<0.001
		E2	100.0	30.0	37	H 100.0	40.0	36	N/A
		SHBG	30.0	17.0	37	H 29.0	13.0	36	N/A
		FAI	65.9	28.2	37	H 76.0	34.5	36	N/A
		DHEA-S	8.5	5.2	37	H 7.3	2.8	36	N/A
Taneja et al. (1994)	PHT	head abnormalities (%)	7.3	5.1	41	H 6.7<br>U 9.4	2.9<br>5.5	28<br>12	N/A
		neck/midpiece abnormalities (%)	1.5	1.9	41	H 0.9<br>U 0.9	0.8<br>0.6	28<br>12	N/A
		total sperm count	52.1	50.2	42	H 239.6<br>U 84.2	113.6<br>91.2	28<br>13	N/A
Xiaotian et al. (2013)	VPA	PRL	12.0	5.0	32	H 5.2	2.0	30	<0.01
		FSH	3.5	1.3	32	H 13.5	3.1	30	<0.01
		LH	3.8	1.6	32	H 6.6	0.8	30	<0.01
		total T	14.1	3.8	32	H 14.8	3.6	30	N/A
		E2	1.7	74.8	32	H 1.6	57.6	30	N/A
		progressive motility (%)	40.9	20.5	15	H 72.7	12.8	25	<0.01
		head abnormalities (%)	61.5	13.7	15	H 11.8	9.0	25	<0.01
		neck/midpiece abnormalities (%)	13.1	3.1	15	H 4.2	4.0	25	<0.01
		tail abnormalities (%)	12.1	3.4	15	H 2.7	2.4	25	<0.01
		sperm concentration	84.6	50.9	15	H 83.6	39.5	25	N/A
		IIEF-5	17.9	5.6	25	H 23.8	1.4	25	<0.01
	LEV	PRL	5.9	2.2	20	H 5.2	2.0	30	N/A

FSH	13.2	4.0	20	H 13.5	3.1	30	N/A
LH	6.5	0.8	20	H 6.6	0.8	30	N/A
total T	15.3	4.1	20	H 14.8	3.6	30	N/A
E2	1.7	48.2	20	H 1.6	57.6	30	N/A
progressive motility (%)	48.7	24.4	10	H 72.7	12.8	25	<0.01
head abnormalities (%)	12.6	9.5	10	H 11.8	9.0	25	N/A
neck/midpiece abnormalities (%)	3.8	4.0	10	H 4.2	4.0	25	N/A
tail abnormalities (%)	3.2	2.7	10	H 2.7	2.4	25	N/A
sperm concentration	73.8	15.6	10	H 83.6	39.5	25	N/A
IIEF-5	19.3	7.5	10	H 23.8	1.4	25	<0.01

ASM, antiseizure medication; CBZ, carbamazepine; LEV, levetiracetam; LTG, lamotrigine; OXC, oxcarbazepine; PHT, phenytoin; VPA, valproate; SD, standard deviation; CI, confidence interval; N, number of participants; N/A, not available; H, healthy control; U, untreated patients

Clinical endpoints were categorized into three groups: **(i) sperm parameters:** progressive motility (%), head, neck/midpiece, and tail abnormalities (%), total sperm count ($\times 10^6$/ejaculation), normal morphology sperm (%), semen volume (ml), and sperm concentration ($\times 10^6$/ml); **(ii) sex hormones and related indicators:** dehydroepiandrosterone sulfate (DHEA-S, µl/dL), estradiol (E2, pmol/L), follicle-stimulating hormone (FSH, mIU/mL), luteinizing hormone (LH, mIU/mL), prolactin (PRL, mIU/L), total testosterone (T, nmol/L), Free T (pmol/L), sex hormone-binding globulin (SHBG, nmol/L), free androgen index (FAI); **(iii) sexual dysfunction** assessed via the International Index of Erectile Function-5 (IIEF-5) questionnaire score (points).

Values are reported to no more than three decimal places, based on the precision of the original publications.

Table S10. Findings on the association between antiseizure medication use and male reproductive disorders via pharmacovigilance and meta-analysis

ASM	Comparator	Endpoint	N of studies (k)	N of subjects (n)	SMD (95% CI)	I² (%)	Prediction interval
Valproate							
		Sex hormone					
	H	DHEA-S	6	270	0.439 (0.202 to 0.676)	0.0	N/E
	U	DHEA-S	3	68	0.158 (−0.211 to 0.526)	0.0	-3.119 to 3.434
	H	E2	6	288	0.504 (−0.061 to 1.068)	7.3	-1.426 to 2.433
	U	E2	3	98	−0.209 (−0.732 to 0.315)	14.8	-6.474 to 6.056
	H	Free testosterone	3	135	−0.750 (−1.475 to −0.026)	12.9	−9.107 to 7.606
	H	FSH	8	380	−0.849 (−1.677 to −0.021)	33.2	−3.832 to 2.134
	U	FSH	5	92	−0.204 (−0.676 to 0.268)	0.0	-1.907 to 1.500
	H	LH	8	380	−0.863 (−1.468 to −0.258)	27.7	−2.966 to 1.240
	U	LH	5	92	−0.531 (−0.786 to −0.276)	0.0	−1.211 to 0.149
	H	PRL	6	283	0.373 (−0.298 to 1.044)	0.8	-1.974 to 2.720
	U	PRL	3	74	0.451 (−0.078 to 0.979)	0.0	-5.925 to 6.827
	H	SHBG	6	270	0.429 (−0.244 to 1.102)	7.2	-1.914 to 2.772
	U	SHBG	3	68	0.135 (−0.341 to 0.611)	8.3	-4.864 to 5.134

	H	Total testosterone	9	431	−0.460 (−0.856 to −0.065)	20.5	−1.781 to 0.861
	U	Total testosterone	6	142	−0.340 (−0.753 to 0.073)	20.4	-1.724 to 1.043
		Sperm parameter					
	H	Head abnormality (%)	3	221	1.637 (−0.323 to 3.597)	49.1	-23.244 to 26.519
	H	Neck/midpiece abnormality (%)	3	221	0.572 (−1.146 to 2.290)	20.4	-21.297 to 22.441
	H	% Progressive motility	4	257	−3.071 (−5.099 to −1.044)	71.5	−12.774 to 6.631
	H	Semen volume	3	217	−0.330 (−1.273 to 0.612)	4.5	-12.018 to 11.357
	H	Sperm concentration	5	310	−0.824 (−1.637 to −0.012)	8.7	−3.907 to 2.259
	H	% Tail abnormality	3	199	1.528 (0.117 to 2.939)	37.6	−16.175 to 19.231
		Sexual dysfunction					
	H	IIEF-5*	3	156	−1.357 (−1.707 to −1.007)	0.0	N/E
Carbamazepine							
		Sex hormone					
	H	DHEA-S*	8	537	−1.194 (−1.786 to −0.602)	7.9	−3.290 to 0.902
	U	DHEA-S*	3	88	−1.834 (−2.910 to −0.759)	4.9	−15.012 to 11.344
	H	E2	5	300	0.317 (−0.267 to 0.901)	20.6	−1.843 to 2.476
	H	FAI*	5	350	−1.453 (−2.032 to −0.873)	14.9	−3.538 to 0.633
	U	FAI*	3	84	−1.244 (−2.364 to −0.123)	0.0	−15.218 to 12.731
	H	Free testosterone*	4	259	−0.740 (−1.320 to −0.161)	12.0	−3.351 to 1.870

	H	FSH	6	355	0.080 (−0.236 to 0.396)	2.2	−0.824 to 0.984
	H	LH*	7	473	0.410 (0.133 to 0.687)	0.0	−0.365 to 1.186
	H	PRL	4	255	0.190 (−0.138 to 0.518)	8.3	−0.947 to 1.327
	H	SHBG*	9	569	1.078 (0.592 to 1.563)	28.2	−0.629 to 2.784
	U	SHBG*	4	99	1.217 (0.508 to 1.926)	0.0	−1.999 to 4.433
	H	Total testosterone	9	569	0.004 (−0.596 to 0.604)	0.0	−2.188 to 2.196
	U	Total testosterone	4	99	−0.151 (−0.514 to 0.213)	6.2	−1.597 to 1.296
Phenytoin							
	Sex hormone						
	H	E2*	3	128	1.164 (0.750 to 1.578)	0.0	−2.263 to 4.590
	H	FAI*	3	117	−1.569 (−2.049 to −1.089)	0.0	−5.550 to 2.413
	H	SHBG*	3	117	2.358 (1.229 to 3.487)	11.0	−11.018 to 15.734
	H	Total testosterone	3	117	1.607 (0.486 to 2.729)	7.8	−11.994 to 15.209

*Not mentioned in the FDA-approved labeling for human males.

ASM, antiseizure medication; CI, confidence interval; DHEA-S, dehydroepiandrosterone sulfate; E2, estradiol; FAI, free androgen index; H, healthy control; IC_{025}, the lower bound of the 95% CI for the information component; IIEF-5, International Index of Erectile Function-5; LH, luteinizing hormone; NS, not significant; N/A, not applicable; N/E, not estimable; PI, prediction interval; PRL, prolactin; ROR, reporting odds ratio; SHBG, sex hormone-binding globulin; SMD, standardized mean difference; U, untreated patients with epilepsy

Table S11. Sensitivity analysis of carbamazepine (Meta-analysis)

Control	Endpoint	Study removed	SMD	SE	95% CI
Healthy control	DHEA-S (μl/L)	Duncan et al. 1999	−1.031	0.298	−1.615 to −0.447
		Isojarvi et al. 1990	−1.182	0.337	−1.843 to −0.521
		Najafi et al. 2012	−1.067	0.315	−1.685 to −0.449
		Ocek et al. 2018	−1.418	0.221	−1.852 to −0.984
		Rattya (B) et al. 2001	−1.196	0.350	−1.881 to −0.511
		Reis et al. 2013	−1.162	0.360	−1.868 to −0.457
		Røste et al. 2005	−1.307	0.329	−1.952 to −0.663
		Svalheim et al. 2009	−1.187	0.360	−1.893 to −0.482
		Pooled effect	−1.194	0.302	−1.786 to −0.602
		Prediction Interval	−1.194	0	−3.290 to 0.902
	FAI	Duncan et al. 1999	−1.141	0.192	−1.517 to −0.765
		Isojarvi et al. 1990	−1.577	0.359	−2.279 to −0.874
		Isojarvi et al. 1995	−1.335	0.321	−1.964 to −0.706
		Reis et al. 2013	−1.575	0.416	−2.390 to −0.761
		Svalheim et al. 2009	−1.621	0.376	−2.358 to −0.884
		Pooled effect	−1.453	0.296	−2.032 to −0.873
		Prediction Interval	−1.453	0	−3.538 to 0.633
	Free T (pmol/L)	Duncan et al. 1999	−0.509	0.290	−1.079 to 0.060
		Isojarvi et al. 1990	−0.983	0.249	−1.472 to −0.495
		Ocek et al. 2018	−0.801	0.391	−1.566 to −0.035
		Svalheim et al. 2009	−0.647	0.438	−1.506 to 0.212
		Pooled effect	−0.740	0.296	−1.320 to −0.161
		Prediction Interval	−0.740	0	−3.351 to 1.870
	LH (mIU/mL)	Isojarvi et al. 1990	0.481	0.138	0.211 to 0.751
		Najafi et al. 2012	0.355	0.151	0.059 to 0.651
		Ocek et al. 2018	0.400	0.167	0.073 to 0.728
		Rattya (B) et al. 2001	0.435	0.163	0.115 to 0.754
		Reis et al. 2013	0.310	0.123	0.069 to 0.551
		Røste et al. 2005	0.475	0.145	0.191 to 0.759
		Svalheim et al. 2009	0.402	0.174	0.062 to 0.743
		Pooled effect	0.410	0.141	0.133 to 0.687
		Prediction Interval	0.410	0	−0.365 to 1.186
	SHBG (nmol/L)	Duncan et al. 1999	0.834	0.169	0.502 to 1.166
		Isojarvi et al. 1990	1.110	0.275	0.571 to 1.648
		Isojarvi et al. 1995	1.034	0.267	0.511 to 1.557
		Najafi et al. 2012	1.103	0.276	0.562 to 1.645
		Ocek et al. 2018	1.115	0.282	0.562 to 0.668
		Rattya (B) et al. 2001	1.151	0.277	0.609 to 1.694
		Reis et al. 2013	1.026	0.275	0.488 to 1.564

		Røste et al. 2005	1.197	0.255	0.697 to 1.697
		Svalheim et al. 2009	1.150	0.283	0.596 to 1.705
		Pooled effect	1.078	0.248	0.592 to 1.563
		Prediction Interval	1.078	0	−0.629 to 2.784
Untreated patients	DHEA-S (μl/L)	Duncan et al. 1999	-1.300	0.247	−1.785 to −0.815
		Geisler et al. 1997	-2.013	0.930	−3.836 to −0.190
		Rattya (A) et al. 2001	-2.209	0.728	−3.636 to −0.782
		Pooled effect	-1.834	0.549	−2.910 to −0.759
		Prediction Interval	-1.834	0	−15.012 to 11.344
	FAI	Duncan et al. 1999	-0.675	0.221	−1.108 to −0.242
		Isojarvi et al. 1995	-1.416	0.939	−3.255 to 0.424
		Rattya (A) et al. 2001	-1.630	0.728	−3.057 to −0.203
		Pooled effect	-1.244	0.572	−2.364 to −0.123
		Prediction Interval	-1.244	0	−15.218 to 12.731
	SHBG (nmol/L)	Duncan et al. 1999	0.853	0.176	0.507 to 1.199
		Geisler et al. 1997	1.357	0.483	0.410 to 2.304
		Isojarvi et al. 1995	1.293	0.491	0.330 to 2.256
		Rattya (A) et al. 2001	1.360	0.469	0.441 to 2.279
		Pooled effect	1.217	0.362	0.508 to 1.926
		Prediction Interval	1.217	0	−1.999 to 4.433

CI, confidence interval; DHEA-S, dehydroepiandrosterone sulfate; FAI, free androgen index; LH, luteinizing hormone; N/A, not available; SE, standard error; SHBG, sex hormone-binding globulin; SMD, standardized mean difference; T, testosterone

Table S12. Sensitivity analysis of phenytoin (Meta-analysis)

Control	Endpoint	Study removed	SMD	SE	95% CI
**Healthy control**	E2 (pmol/L)	Duncan et al. 1999	0.927	0.245	0.447 to 1.406
		Heroz et al. 1991	1.235	0.324	0.600 to 1.869
		Isojarvi et al. 1990	1.273	0.276	0.732 to 1.814
		Pooled effect	1.164	0.211	0.750 to 1.578
		Prediction Interval	1.164	N/A	−2.263 to 4.590
	FAI	Duncan et al. 1999	-1.302	0.281	−1.852 to −0.752
		Isojarvi et al. 1990	-1.763	0.273	−2.299 to −1.227
		Isojarvi et al. 1995	-1.611	0.388	−2.371 to – 0.851
		Pooled effect	-1.569	0.245	−2.049 to −1.089
		Prediction Interval	-1.569	N/A	−5.550 to 2.413
	SHBG (nmol/L)	Duncan et al. 1999	1.769	0.300	1.181 to 2.358
		Isojarvi et al. 1990	2.763	0.847	1.104 to 4.423
		Isojarvi et al. 1995	2.603	0.997	0.650 to 4.557
		Pooled effect	2.358	0.576	1.229 to 3.487
		Prediction Interval	2.358	N/A	−11.018 to 15.734
	total T (nmol/L)	Duncan et al. 1999	1.025	0.271	0.494 to 1.557
		Isojarvi et al. 1990	1.916	0.903	0.148 to 3.685
		Isojarvi et al. 1995	1.910	0.900	0.147 to 3.674
		Pooled effect	1.607	0.572	0.486 to 2.729
		Prediction Interval	1.607	N/A	−11.994 to 15.209

CI, confidence interval; E2, estradiol; FAI, free androgen index; N/A, not available; SE, standard error; SHBG, sex hormone-binding globulin; SMD, standardized mean difference;

Table S13. Sensitivity analysis of valproate (Meta-analysis)

Control	Endpoint	Study removed	SMD	SE	95% CI
**Healthy control**	DHEA-S (µl/L)	Duncan et al. 1999	0.377	0.142	0.098 to 0.656
		Isojarvi et al. 1990	0.433	0.137	0.164 to 0.702
		Najafi et al. 2012	0.478	0.130	0.223 to 0.734
		Ocek et al. 2018	0.345	0.135	0.081 to 0.610
		Rattya (B) et al. 2001	0.511	0.132	0.252 to 0.770
		Røste et al. 2005	0.460	0.141	0.185 to 0.736
		Pooled effect	0.439	0.121	0.202 to 0.676
		Prediction Interval	N/A	N/A	N/A
	free T (nmol/L)	Duncan et al. 1999	-0.433	0.328	−1.077 to 0.210
		Isojarvi et al. 1990	-1.032	0.367	−1.751 to −0.313
		Ocek et al. 2018	-0.743	0.708	−2.131 to 0.644
		Pooled effect	-0.750	0.370	−1.475 to −0.026
		Prediction Interval	-0.750	N/A	−9.107 to 7.606
	FSH (mIU/mL)	Guo et al. 2021	-0.920	0.499	−1.899 to 0.058
		Isojarvi et al. 1990	-0.862	0.469	−1.780 to 0.057
		Nag et al. 1997	-1.069	0.439	−1.929 to −0.208
		Najafi et al. 2012	-0.977	0.478	−1.913 to −0.041
		Ocek et al. 2018	-0.965	0.497	−1.938 to 0.009
		Rattya (B) et al. 2001	-0.838	0.487	−1.792 to 0.117
		Røste et al. 2005	-0.801	0.478	−1.738 to 0.135
		Xiaotian et al. 2013	-0.383	0.244	−0.860 to 0.095
		Pooled effect	-0.849	0.422	−1.677 to −0.021
		Prediction Interval	-0.849	N/A	−3.832 to 2.134
	LH (mIU/mL)	Guo et al. 2021	-0.975	0.352	−1.666 to −0.285
		Isojarvi et al. 1990	-0.641	0.282	−1.193 to −0.089
		Nag et al. 1997	-0.855	0.353	−1.547 to −0.163
		Najafi et al. 2012	-0.960	0.349	−1.644 to −0.276
		Ocek et al. 2018	-1.015	0.327	−1.656 to −0.374
		Rattya (B) et al. 2001	-0.941	0.358	−1.642 to −0.240
		Røste et al. 2005	-0.903	0.358	−1.605 to −0.200
		Xiaotian et al. 2013	-0.628	0.257	−1.131 to −0.124
		Pooled effect	-0.863	0.309	−1.468 to −0.258
		Prediction Interval	-0.863	N/A	−2.966 to 1.240
	total T (nmol/L)	Duncan et al. 1999	-0.506	0.237	−0.970 to −0.042
		Guo et al. 2021	-0.534	0.221	−0.966 to –0.102
		Isojarvi et al. 1990	-0.502	0.218	−0.928 to –0.075
		Nag et al. 1997	-0.305	0.166	–0.629 to 0.020
		Najafi et al. 2012	-0.317	0.173	–0.655 to 0.022
		Ocek et al. 2018	-0.511	0.229	−0.961 to −0.062

		Rattya (B) et al. 2001	-0.516	0.225	−0.956 to −0.076
		Røste et al. 2005	-0.459	0.227	−0.905 to −0.013
		Xiaotian et al. 2013	-0.501	0.232	−0.956 to −0.046
		Pooled effect	-0.460	0.202	−0.856 to −0.065
		Prediction Interval	-0.460	N/A	−1.781 to 0.861
	progressive motility (%)	Guo et al. 2021	−3.808	1.682	−7.105 to −0.512
		Hamed et al. 2015	−1.267	0.397	−2.045 to −0.489
		Røste et al. 2003	−4.052	1.557	−7.104 to −1.000
		Xiaotian et al. 2013	−3.540	1.466	−6.414 to −0.667
		Pooled effect	−3.071	1.034	−5.099 to −1.044
		Prediction Interval	−3.071	N/A	−12.774 to 6.631
	sperm concentration ($\times 10^6$/ml)	Guo et al. 2021	−0.669	0.486	−1.621 to 0.283
		Hamed et al. 2015	−0.478	0.318	−1.102 to 0.146
		Ocek et al. 2018	−0.946	0.529	−1.982 to 0.090
		Røste et al. 2003	−1.002	0.500	−1.981 to −0.022
		Xiaotian 2013	−1.033	0.477	−1.967 to −0.099
		Pooled effect	−0.824	0.415	−1.637 to −0.012
		Prediction Interval	−0.824	N/A	−3.907 to 2.259
	tail abnormality (%)	Ocek et al. 2018	2.148	1.243	−0.289 to 4.584
		Røste et al. 2003	1.892	1.504	−1.056 to 4.841
		Xiaotian et al. 2013	0.678	0.261	0.166 to 1.190
		Pooled effect	1.528	0.720	0.117 to 2.939
		Prediction Interval	1.528	N/A	−16.175 to 19.231
	IIEF-5 (points)	Guo et al. 2021	−1.481	0.223	−1.918 to −1.044
		Ocek et al. 2018	−1.279	0.217	−1.705 to −0.853
		Xiaotian et al. 2013	−1.317	0.216	−1.740 to −0.893
		Pooled effect	−1.357	0.179	−1.707 to −1.007
		Prediction Interval	N/A	N/A	N/A
**Untreated patients**	LH (mIU/mL)	Geisler et al. 1997	-0.553	0.150	−0.846 to −0.259
		Guo et al. 2021	-0.652	0.121	−0.889 to −0.415
		Mohamed et al. 2019	-0.429	0.117	−0.658 to −0.201
		Nag et al. 1997	-0.483	0.169	−0.815 to −0.152
		Rattya (A) et al. 2001	-0.555	0.160	−0.869 to −0.242
		Pooled effect	−0.531	0.130	−0.786 to −0.276
		Prediction Interval	−0.531	N/A	−1.211 to 0.149

CI, confidence interval; DHEA-S, dehydroepiandrosterone sulfate; FSH, follicle-stimulating hormone; IIEF-5, International Index of Erectile Function-5; LH, luteinizing hormone; N/A, not available; SE, standard error; SMD, standardized mean difference; T, testosterone

Table S14. Sensitivity analysis of alternative correlation coefficients (Meta-analysis)

Analysis	Control	Endpoint	pooled SMD (95% CI)		
			r=0.30	r=0.80	r=0.59
Carbamazepine	H	DHEA-S	−1.246 (−1.860 to −0.631)	−1.112 (−1.627 to −0.596)	−1.194 (−1.786 to −0.602)
Carbamazepine	H	FAI	−1.518 (−2.143 to −0.892)	−1.311 (−1.727 to −0.894)	−1.453 (−2.032 to −0.873)
Carbamazepine	H	Free T	−0.809 (−1.491 to −0.127)	−0.669 (−1.088 to −0.250)	−0.740 (−1.320 to −0.161)
Carbamazepine	H	LH	0.410 (0.133 to 0.687)	0.410 (0.133 to 0.687)	0.410 (0.133 to 0.687)
Carbamazepine	H	SHBG	1.065 (0.601 to 1.529)	1.011 (0.542 to 1.481)	1.078 (0.592 to 1.563)
Valproate	H	DHEA-S	0.443 (0.178 to 0.709)	0.398 (0.193 to 0.603)	0.439 (0.202 to 0.676)
Valproate	H	FSH	−0.849 (−1.677 to −0.021)	−0.849 (−1.677 to −0.021)	−0.849 (−1.677 to −0.021)
Valproate	H	LH	−0.863 (−1.468 to −0.258)	−0.863 (−1.468 to −0.258)	−0.863 (−1.468 to −0.258)
Valproate	H	Total T	−0.472 (−0.878 to −0.065)	−0.448 (−0.830 to −0.066)	−0.460 (−0.856 to −0.065)
Valproate	H	Progressive motility	−3.071 (−5.099 to −1.044)	−3.071 (−5.099 to −1.044)	−3.071 (−5.099 to −1.044)
Valproate	H	Sperm concentration	−0.824 (−1.637 to −0.012)	−0.824 (−1.637 to −0.012)	−0.824 (−1.637 to −0.012)
Valproate	H	Tail abnormality	1.528 (0.117 to 2.939)	1.528 (0.117 to 2.939)	1.528 (0.117 to 2.939)
Valproate	H	IIEF-5	−1.357 (−1.707 to −1.007)	−1.357 (−1.707 to −1.007)	−1.357 (−1.707 to −1.007)
Valproate	U	LH	−0.548 (−0.811 to −0.285)	−0.493 (−0.749 to −0.237)	−0.531 (−0.786 to −0.276)

Analysis	Control	Endpoint	pooled SMD (95% CI)		
			**r=0.30**	**r=0.80**	**r=0.59**
Phenytoin	H	E2	1.227 (0.592 to 1.862)	1.019 (0.743 to 1.296)	1.164 (0.750 to 1.578)
Phenytoin	H	FAI	−1.664 (−2.425 to −0.903)	−1.359 (−1.671 to −1.047)	−1.569 (−2.049 to −1.089)
Phenytoin	H	SHBG	2.535 (1.160 to 3.910)	2.105 (1.529 to 2.681)	2.358 (1.229 to 3.487)
Phenytoin	H	Total T	1.765 (0.460 to 3.070)	1.396 (0.697 to 2.096)	1.607 (0.486 to 2.729)

CI, confidence interval; DHEA-S, dehydroepiandrosterone sulfate (µl/dL); E2, estradiol (pmol/L); FAI, free androgen index; FSH, follicle-stimulating hormone (mIU/mL); LH, luteinizing hormone (mIU/mL); H, healthy control; IIEF-5, the International Index of Erectile Function-5 questionnaire score (points); SHBG, sex hormone-binding globulin (nmol/L); SMD, standardized mean difference; T, testosterone (nmol/L); U, untreated patients with epilepsy

Table S15. Demographic characteristics of patients with AED-associated male reproductive disorders (FAERS analysis)

Demographic characteristics	Values (n = 47)
Age, mean (SD)	41.73 (19.29)
<18	2 (4.26)
18-29	5 (10.64)
30-39	8 (17.02)
40-49	2 (4.26)
50-64	5 (10.64)
>=65	5 (10.64)
Not reported	20 (42.55)
Reporting year, n (%)	
2019	4 (8.51)
2020	6 (12.77)
2021	14 (29.79)
2022	5 (10.64)
2023	9 (19.15)
2024	2 (4.26)
2025	6 (12.77)
2026	1 (2.13)
Reporter regions, n (%)	
Europe	28 (59.57)
North America	16 (34.04)
South America	2 (4.26)
Asia	1 (2.13)

SD, standard deviation

Table S16. The primary suspected antiseizure medications and disproportionality analysis results for male reproductive disorders (FAERS analysis)

Primary suspected drugs	Preferred terms	A	B	C	D	ROR (95% CI)	IC_{025}
Valproate	total	21	4,904	2,935	3,664,830	5.347 (3.478 to 8.221)	1.792
	testicular disorder	5	-	-	-	-	-
	testicular atrophy	5	-	-	-	-	-
	infertility	4	-	-	-	-	-
	sperm concentration decreased	3	-	-	-	-	-
	azoospermia	3	-	-	-	-	-
	spermatozoa morphology abnormal	2	-	-	-	-	-
	spermatozoa progressive motility decreased	2	-	-	-	-	-
	varicocele	1	-	-	-	-	-
	oligoasthenozoospermia	1	-	-	-	-	-
	hypogonadism	1	-	-	-	-	-
	teratospermia	1	-	-	-	-	-
	hypogonadism male	1	-	-	-	-	-
	secondary hypogonadism	1	-	-	-	-	-
	genital atrophy	1	-	-	-	-	-
	semen viscosity decreased	1	-	-	-	-	-
Gabapentin	total	9	9,230	2,947	3,660,504	1.211 (0.629 to 2.331)	−0.665
	hypogonadism male	3	-	-	-	-	-
	hypogonadism	3	-	-	-	-	-
	haematospermia	2	-	-	-	-	-
	testicular atrophy	1	-	-	-	-	-
Levetiracetam	total	8	12,083	2,948	3,657,651	0.821 (0.410 to 1.645)	−1.281

	sperm concentration abnormal	5	-	-	-	-	-
	infertility male	2	-	-	-	-	-
	oligospermia	1	-	-	-	-	-
	testicular atrophy	1	-	-	-	-	-
Lamotrigine	total	3	4,057	2,953	3,665,677	0.918 (0.296 to 2.849)	−1.754
	hypogonadism male	2	-	-	-	-	-
	oligospermia	1	-	-	-	-	-
	semen volume decreased	1	-	-	-	-	-
Oxcarbazepine	total	2	1,451	2,954	3,668,283	1.712 (0.427 to 6.854)	−1.223
	hypogonadism male	1	-	-	-	-	-
	spermatozoa progressive motility decreased	1	-	-	-	-	-
Carbamazepine	total	1	3,194	2,955	3,666,540	0.388 (0.055 to 2.760)	−4.189
	azoospermia	1	-	-	-	-	-
Phenytoin	total	1	1,999	2,955	3,667,735	0.621 (0.087 to 4.412)	−3.513
	secondary hypogonadism	1	-	-	-	-	-
Clobazam	total	1	915	2,955	3,668,819	1.357 (0.191 to 9.647)	−2.386
	hypogonadism male	1	-	-	-	-	-
Pregabalin	total	1	11,826	2,955	3,657,908	0.105 (0.015 to 0.743)	−6.078
	semen volume decreased	1	-	-	-	-	-
Brivaracetam		0	1,509	2,956	3,668,225	N/A	N/A
Clonazepam		0	3,439	2,956	3,666,295	N/A	N/A
Eslicarbazepine		0	406	2,956	3,669,328	N/A	N/A
Ethosuximide		0	106	2,956	3,669,628	N/A	N/A
Felbamate		0	18	2,956	3,669,716	N/A	N/A
Lacosamide		0	3,643	2,956	3,666,091	N/A	N/A

Perampanel	0	595	2,956	3,669,139	N/A	N/A
Phenobarbital	0	143	2,956	3,669,591	N/A	N/A
Rufinamide	0	54	2,956	3,669,680	N/A	N/A
Topiramate	0	2,042	2,956	3,667,692	N/A	N/A
Vigabatrin	0	2,272	2,956	3,667,462	N/A	N/A
Zonisamide	0	194	2,956	3,669,540	N/A	N/A

ASM, antiseizure medication; A, number of qualifying reports (SMQ 'Fertility disorders', narrow-scope male-specific/sex-neutral PTs) with the ASM as a primary suspected drug; B, number of other AE reports for the ASM; C, number of qualifying reports for all other drugs; D, number of other AE reports for all other drugs; CI, confidence interval; IC_{025}, lower bound of the 95% credibility interval for the information component; N/A, not applicable (fewer than one report, disproportionality measures not estimable); ROR, reporting odds ratio. Values correspond to Scenario S1_PRIMARY, the primary disproportionality analysis using all other drugs as the comparator.

Table S17. Comparative overview of regulatory approvals by country

AED	Drug Label		
	MFDS	FDA	EMA
Carbamazepine	4. ADVERSE REACTIONS<br>Reproductive System – Very Rare (<1/10,000)<br>Sexual dysfunction, erectile dysfunction, abnormal spermatogenesis (reduced sperm count and/or motility)<br>14) Decreased male fertility and abnormal spermatogenesis have been reported, although the causal relationship is not clearly established.	ADVERSE REACTIONS<br>Genitourinary System: There have been rare reports of impaired male fertility and/or abnormal spermatogenesis.<br>Testicular atrophy occurred in rats receiving carbamazepine orally from 4-52 weeks at dosage levels of 50-400 mg/kg/day. Additionally, rats receiving carbamazepine in the diet for 2 years at dosage levels of 25, 75, and 250 mg/kg/day had a dose-related incidence of testicular atrophy and aspermatogenesis. In dogs, it produced a brownish discoloration, presumably a metabolite, in the urinary bladder at dosage levels of 50 mg/kg/day and higher. Relevance of these findings to humans is unknown.	N/A
Phenytoin	N/A	13.1 Carcinogenesis, Mutagenesis, Impairment of Fertility<br><u>Fertility:</u> Phenytoin has not been adequately assessed for effects on male or female fertility.	N/A
Valproate	4. ADVERSE REACTIONS<br>(PO) Endocrine System – Androgen Excess/ (IV) Endocrine and	6.4 Postmarketing Experience<br><u>Endocrine:</u> hyperandrogenism, elevated testosterone level	Several tests for chromosome damage were positive. Severe chromosome damage is expected to lead to death of sperm cells /

	Reproductive Systems – Rare – Male Infertility<br>7. ADMINISTRATION TO PREGNANT AND LACTATING WOMEN<br>(PO/IV) Fertility – Administration of valproic acid may impair fertility in males.	<u>Reproductive:</u> Aspermia, azoospermia, decreased sperm count, decreased spermatozoa motility, male infertility, and abnormal spermatozoa morphology.<br>8.3 Females and Males of Reproductive Potential<br><u>Infertility</u>: There have been reports of male infertility coincident with valproate therapy [see Adverse Reactions (6.4)]. In animal studies, oral administration of valproate at clinically relevant doses resulted in adverse reproductive effects in males [see Nonclinical Toxicology (13.1)].<br>13.1 Carcinogenesis, Mutagenesis, and Impairment of Fertility<br><u>Impairment of Fertility:</u> In chronic toxicity studies in juvenile and adult rats and dogs, administration of valproate resulted in testicular atrophy and reduced spermatogenesis at oral doses of 400 mg/kg/day or greater in rats (approximately equal to or greater than the maximum recommended human dose (MRHD) on a mg/m2 basis) and 150 mg/kg/day or greater in dogs (approximately equal to or greater than the MRHD on a mg/m2	reduced fertility and not to transmission of mutations to the offspring. It is unknown whether slight chromosome damage might be transmitted to the offspring. Further investigation is recommended by PRAC.

		basis). Fertility studies in rats have shown no effect on fertility at oral doses of valproate up to 350 mg/kg/day (approximately equal to the MRHD on a mg/m^2 basis) for 60 days.	
Levetiracetam	N/A	13.1 Carcinogenesis, Mutagenesis, Impairment of Fertility<br>Impairment of Fertility: No adverse effects on male or female fertility or reproductive performance were observed in rats at oral doses up to 1800 mg/kg/day (6 times the maximum recommended human dose on a mg/m^2 or systemic exposure [AUC] basis).	4.6 Fertility, pregnancy and lactation<br>Fertility<br>No impact on fertility was detected in animal studies (see section 5.3). No clinical data are available, potential risk for human is unknown.

AED, anti-epileptic drug; EMA, European Medicines Agency; FDA, Food and Drug Administration; MFDS, Ministry of Food and Drug Safety; N/A, not available
