# Supplementary material for: Male Reproductive Disorders Associated with Antiseizure Medications: Evidence from Meta-Analysis and FAERS Pharmacovigilance Study
Source: J Clin Med. 2026 Sep 29;15(19):7566. doi: 10.3390/jcm15197566 (PMC13666318; doi:10.3390/jcm15197566)
Supplement: Supplementary file 1 [file jcm-15-07566-s001.zip › Supplementary figures_JCM_revised_final.pdf]

Figure S1. Flow diagram of pharmacovigilance assessment of male reproductive disorder-associated with ASMs (FAERS analysis)

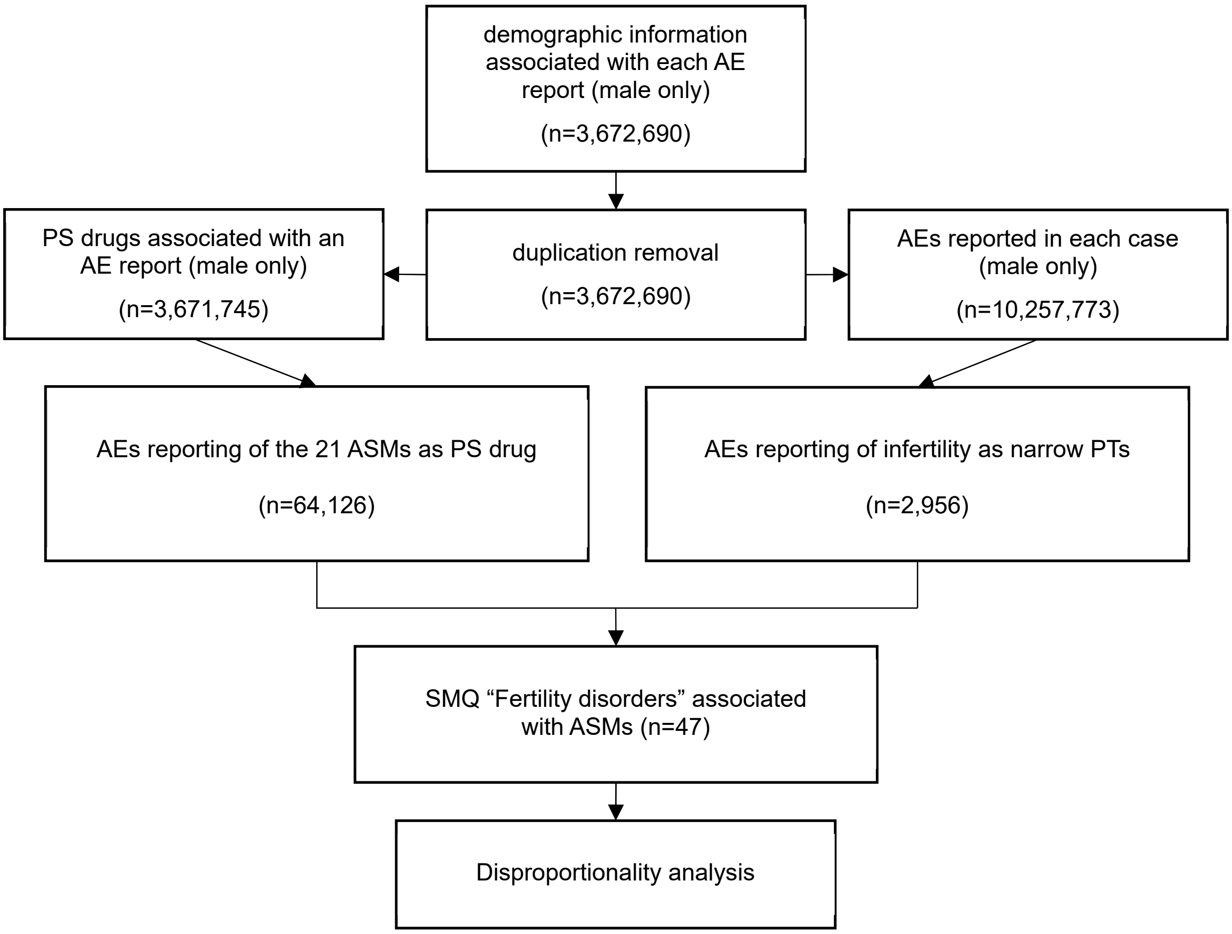

n, number of AE reports; PS, primary suspected; AEs, adverse events; ASMs, antiseizure medications; PTs, preferred terms; SMQ, Standardized MedDRA Queries
